# Study of the Addition Mechanism of 1*H*-Indazole and Its 4-, 5-, 6-, and 7-Nitro Derivatives to Formaldehyde in Aqueous Hydrochloric Acid Solutions

**DOI:** 10.1021/acs.joc.2c00154

**Published:** 2022-04-11

**Authors:** Ibon Alkorta, Rosa M. Claramunt, José Elguero, Enrique Gutiérrez-Puebla, M. Ángeles Monge, Felipe Reviriego, Christian Roussel

**Affiliations:** † Instituto de Química Médica, CSIC, Juan de la Cierva, 3, E-28006 Madrid, Spain; ‡ Departamento de Química Orgánica y Bio-Orgánica, Facultad de Ciencias, UNED, Senda del Rey 9, E-28040 Madrid, Spain; § Departamento de Nuevas Arquitecturas en Química de Materiales, Instituto de Ciencia de Materiales de Madrid (ICMM-CSIC), Sor Juana Inés de la Cruz, 3, Cantoblanco, E-28049 Madrid, Spain; ∥ Instituto de Ciencia y Tecnología de Polímeros, CSIC, Juan de la Cierva, 3, E-28006 Madrid, Spain; ⊥ Aix-Marseille Université, CNRS, Centrale Marseille, iSm2, 13397 Marseille, France

## Abstract

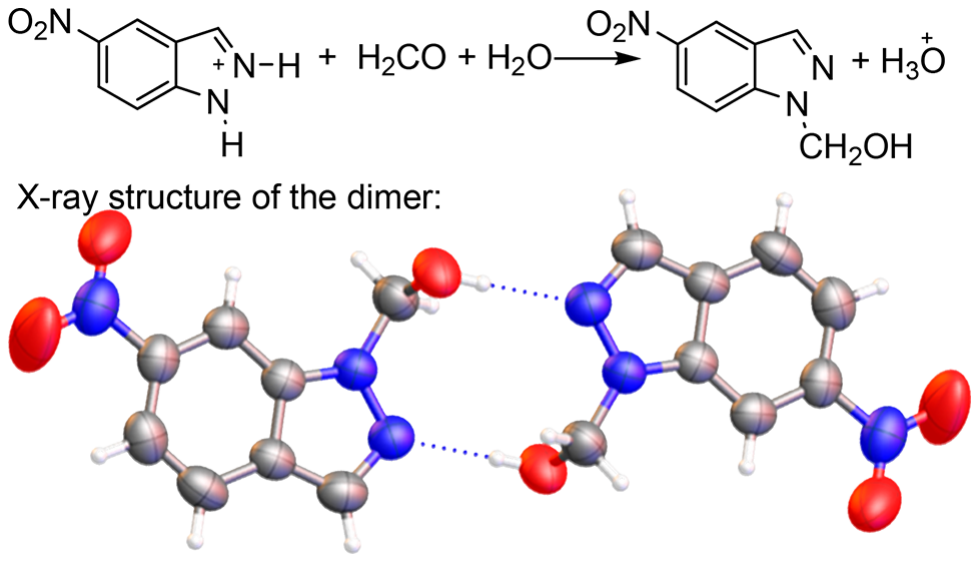

The reaction of *NH*-indazoles with formaldehyde in aqueous hydrochloric acid has been experimentally studied by solution and solid-state nuclear magnetic resonance (NMR) and crystallography. The mechanism of the formation of *N*_1_-CH_2_OH derivatives was determined. For the first time, 2-substituted derivatives have been characterized by multinuclear NMR. Theoretically, calculations with gauge-invariant atomic orbitals (GIAOs) at the Becke three-parameter (exchange) Lee–Yang–Parr B3LYP/6-311++G(d,p) level have provided a sound basis for the experimental observations. The first X-ray structures of four (1*H*-indazol-1-yl)methanol derivatives are reported.

## Introduction

**Scheme 1:**
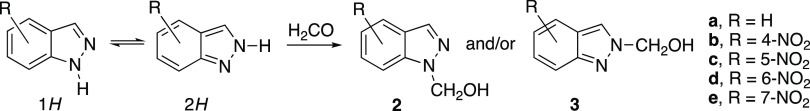
Reaction Studied in the Present Work

This work was aimed at a better understanding of a characteristic reaction of *N*-unsubstituted azoles and their reaction with formaldehyde to afford azolylmethanols. As a model of azole, indazole was selected because it was not clear what isomer would be obtained depending on the substituents in the ring. After solving this problem for 4-, 5-, 6-, and 7-nitro derivatives, the mechanism of the reaction should be established because it is common to all azoles and that azolylmethanols are the intermediates, directly and indirectly (using hydroxymethyl as a protecting group) to other compounds relevant for their applications. The present paper reports our study of the reaction of five *NH*-indazoles with formaldehyde in an aqueous acid solution, Scheme 1.

A search in different databases shows that the chemistry of indazoles is a very active field; the numbers of items are 11 723 (Scifinder),^1^ 5142 (ScienceDirect),^2^ and 4448 (Web of Science);^3^ and most of the papers and patents deal with biological applications.^4−8^ Other applications (corrosion inhibitors)^9^ and synthetic methods^10^ are less reported, and the last place is occupied by indazole reactivity.

**Figure 1:**
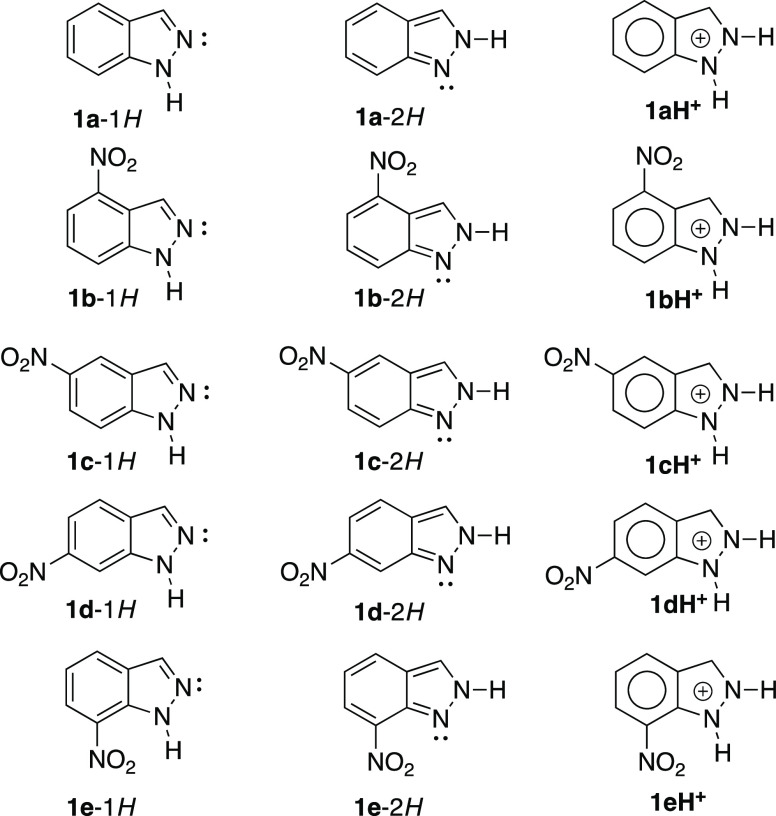
Five neutral **1a–1e** and protonated indazoles **1aH**^**+**^**–1eH**^**+**^.

Five indazoles **1a–1e**, existing in two tautomeric forms 1*H* and 2*H*, and their protonated indazolium cations **1aH**^**+**^**–1eH**^**+**^, covering all of the substituted nitro compounds in the six-membered ring, will be discussed (Figure 1).

Some nitro-1*H*-indazoles, bearing or not other C-substituents, are powerful inhibitors of nitric oxide synthase isoforms, nNOS, iNOS, and eNOS.^11^ Of the five possible C-nitro-1*H*-indazoles, 3-, 4-, 5-, 6-, and 7-, only 7-nitro-1*H*-indazoles (7-nitro, 3-bromo-7-nitro, and 3,7-dinitro) have inhibitory properties.^12−14^

**Scheme 2:**
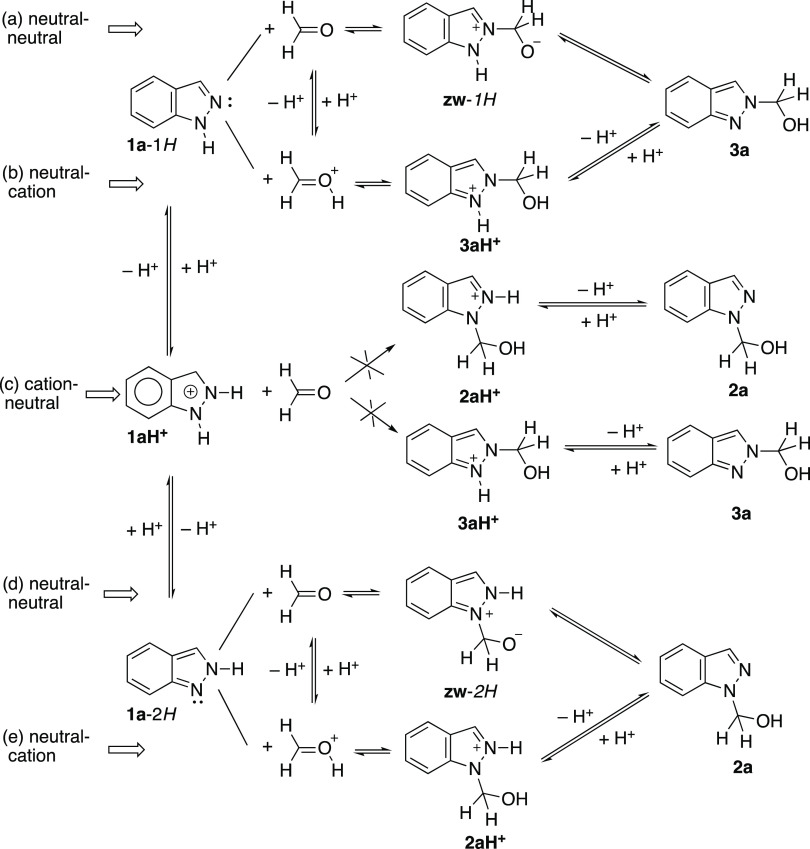
Formal Reactions between Both Tautomers of Indazole **1a** and Indazolium Cation **1aH**^**+**^ with Formaldehyde Corresponding to Neutral and to Acid Conditions

In 1969,^15^ we reported that indazoles react with formaldehyde in aqueous HCl to afford (1*H*-indazol-1-yl)methanol derivatives. Indazole itself (**1a**) and 4-nitro (**1b**), 5-nitro (**1c**), and 6-nitro-1*H*-indazoles (**1d**) react, but 7-nitro-1*H*-indazole (**1e**) does not. The isolated compounds were characterized by ^1^H NMR in DMSO-*d*_6_, proving that they were 1-substituted indazoles. In 1986, the reaction was carried out in neutral conditions (ethanol).^16^ In 2004,^17^ the reaction of indazole **1a** in acid conditions was re-examined; B3LYP/6-311++G(d,p) calculations indicated that the 1-substituted isomer (**2a**) was 20 kJ·mol^–1^ more stable than the 2-substituted isomer (**3a**) (Scheme 2), and the NMR data were extended to ^13^C and ^15^N nuclei together with GIAO calculations of absolute shieldings.

This reaction is common to all azoles (pyrazole in acid^15^ and neutral conditions,^16,18,19^ imidazole,^20,21^ triazoles,^22−24^ tetrazole,^25^ benzimidazole,^16,26^ and benzotriazole).^27−29^ In the case of indazole, previous to our works,^15−17^ Pozharskii et al. carried the reaction in 1964 in acid media.^30^ Some azoles have two different tautomers; this is the case for 1,2,3-triazole, 1,2,4-triazole, tetrazole, indazole and benzotriazole; for these azoles, tautomer and isomer structures (position of the N*H*/N*R*) often differ according to the Curtin–Hammett principle and the Winstein–Holnes equation.^31^ In the case of indazole **1a**, MP2/6-31G** calculations indicate that the 1*H*-tautomer is 15 kJ·mol^–1^ more stable than the 2*H* tautomer.^32^ Similar values were obtained by other authors in the gas phase (14.5 kJ·mol^–1^) and in water (15.9 kJ·mol^–1^).^33^

In summary, the theoretical results we have reported above concern exclusively thermodynamic aspects, differences in energy between tautomers and isomers, and NMR chemical shifts. Note that there were no theoretical studies on the reaction mechanism.

Although the reaction can occur in neutral conditions, we have carried out our calculations on indazolium cations because our experimental procedure always includes hydrochloric acid.

There are two ways to prepare compounds **2a** and **3a**, from neutral indazoles **1a**-1*H* and **1a**-2*H*, reacting with neutral formaldehyde (Scheme 2, a and d reactions) or with protonated formaldehyde (Scheme 2, b and e reactions), or from protonated indazole **1aH**^**+**^ (Scheme 2, c reaction). Obviously, the mechanism should involve protonated formaldehyde because it is a much weaker base (p*K*_a_ = −4.2)^34^ than indazoles (**1a**, 1.04; **1b**, 0.24; **1c**, −0.96; **1d**, −0.97; **1e**, −0.99).^35^ Therefore, it is impossible to have a direct reaction between the indazolium cation and neutral or protonated formaldehyde (Scheme 2c reaction). We will see afterward how the reaction could involve indazolium cations with a relayed catalysis by a water molecule. In neutral conditions, zwitterions, **zw**, are intermediates to **3a** and **2a**.

The addition of azoles to carbonyl compounds is a reversible reaction that is more complete with aldehydes (for formaldehyde, see the Introduction section; for other aldehydes, see ref (36)) than with ketones like acetone.^37,38^ The reverse reaction (elimination) is very fast in the ketone adduct and rather slow in the aldehyde adduct; the combination of these two reaction rates (addition and elimination) accounts for the position of the equilibrium to the point that has incorrectly been named irreversible for formaldehyde adducts. It depends also on the azole where electron-withdrawing groups like nitro substituents increase the sensitivity to hydrolysis, *i.e*., to an increase of the reverse reaction rate due to the increased leaving group character for nitro derivatives. The pure samples prepared in 1968^15^ contain in 2021 about 50% of free *NH*-indazole, that is, *t*_1/2_ ∼50 to 55 years in the solid state in a sealed tube (possibly formaldehyde polymerize into trioxymethylene or into paraformaldehyde). Starting from a pure adduct, crystallization in boiling water also leads to its partial decomposition.

Compounds **2aH**^**+**^ and **3aH**^**+**^ are in a formal way hemiaminals^39−41^ where the usual loss of water would lead to 1-methylene-1*H*-indazol-1-iums, a class of unknown nonaromatic cations. In most cases, the synthetic procedure we have used affords a pure compound (^1^H NMR of the crude), but crystallization in boiling water reverts the reaction and mixtures of the adduct and free indazole are obtained in proportions close to 50:50.

## Results and Discussion

After reporting the synthetic schemes, we will establish the structures of the different hydroxymethyl-indazoles we have identified in this work. Since some of them are formed in small quantities or are unstable, we have followed a logical chain (1) to determine by X-ray crystallography the structure of all possible compounds, *i.e.*, obtain crystals of all abundant and stable compounds; (2) to carry out GIAO/DFT calculations to confirm the assignment of the NMR spectra; (3) to record the solid-state NMR spectra (CPMAS) of the compounds whose X-ray structures have been determined; and (4) to register solution NMR spectra of all of the compounds and compare the NMR chemical shifts determined in solution with GIAO/DFT-calculated values to identify the structures that cannot be isolated.

### Synthesis

**Scheme 3:**
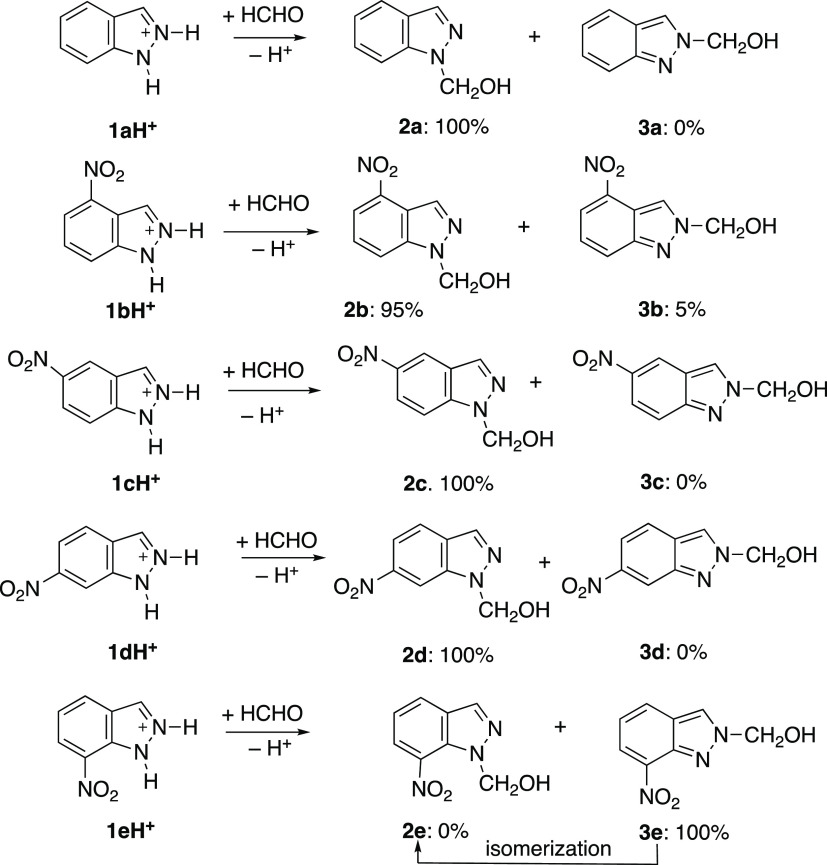
Reaction of Indazoles with Formaldehyde; Given Also the Relative Ratios and the Isomerization Case

The synthetic procedure reported in ref (15) (Scheme 3) was used with some differences. In the present work, we employ longer times and more water, particularly in the case of 7-nitro-1*H*-indazole (**1e**) that according to a previous report does not react with formaldehyde.^15^ In this last case, the effect of much longer times and microwave irradiation was also explored.

Crystallization in boiling water affords pure **2a**; however, in the case of **2b**, **2c**, and **2d**, it results in the partial hydrolysis of the *N*-substituent with formation of **1b**, **1c**, and **1d** (between 33 and 50% determined by integration of the ^1^H NMR spectrum). In Experimental Section, a detailed procedure on how to obtain suitable crystals for X-ray crystallography by avoiding decomposition is described.

In summary, according to the NMR results reported next, the reactions in HCl (aq) correspond to **1a** → **2a**, **1b** → **2b** (95%) + **3b** (5%), **1c** → **2c**, **1d** → **2d**, and **1e** → **3e**; neutral **3e** decomposes into **1e** plus a small isomerization into **2e**. Although the reactions in HCl (aq) should afford the indazolium salts, **2aH**^**+**^, **2bH**^**+**^, **3bH**^**+**^, **2cH**^**+**^, **2dH**^**+**^, and **3eH**^**+**^, the insolubility in water of the neutral indazoles makes that they precipitate by the addition of water. It is important to note that **3b** is formed in an acid medium, while **2e** is formed in a neutral solution.

### X-ray Crystallography

**Figure 2:**
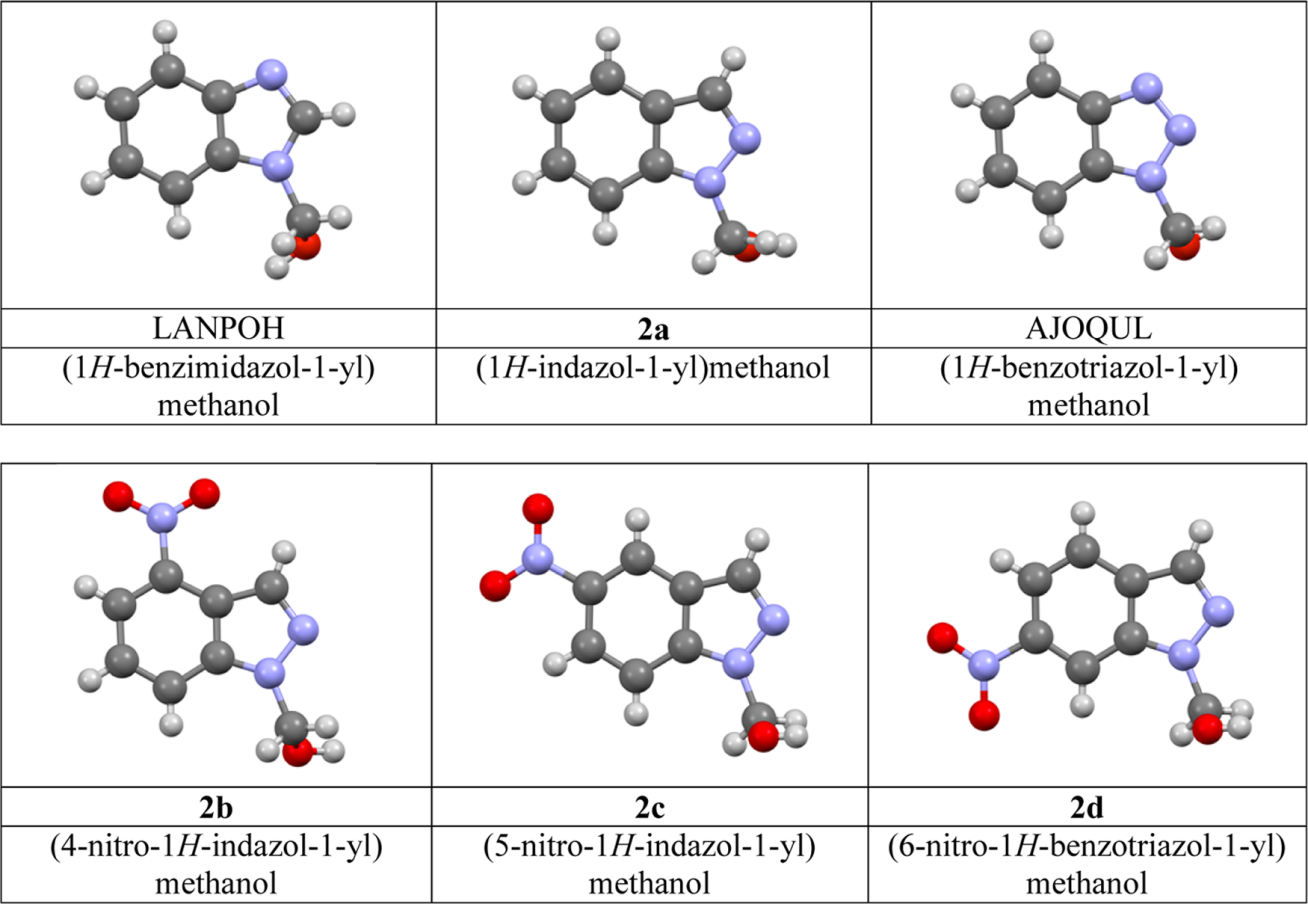
1-Methanol derivatives of the three parent benzazoles (top) and 1-methanol derivatives of three C-nitro indazoles (bottom). The structure of **2c** contains a molecule of dioxane (not represented).

No X-ray structures of *N*-methanol derivatives of indazoles are known, but those of benzimidazole and benzotriazole analogues are reported in the CSD;^42^ they correspond to the refcodes LANPOH^43^ and AJOQUL,^17^ respectively (Figure 2).

We have succeeded in obtaining crystals good enough to solve the structures of parent compound **2a** and those of the three nitro derivatives in Figure 2.

**Figure 3:**
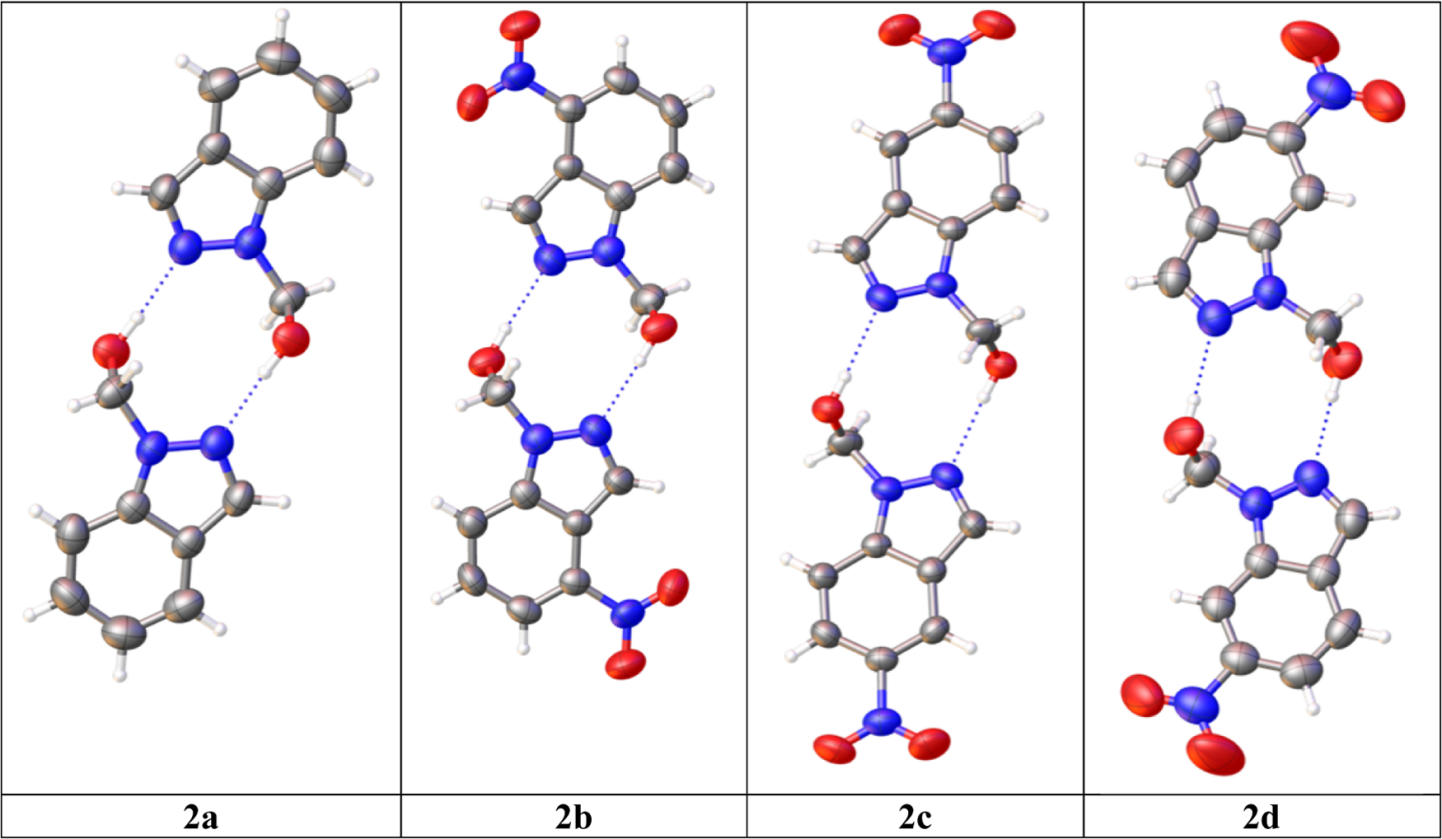
Four X-ray structures. The thermal ellipsoids are set at a 50% probability level.

The four structures very similarly form dimers through intermolecular O–H···N hydrogen bonds (HBs) (Figure 3). For compound **2c**, the crystallization molecule of dioxane is not represented.

**Figure 4:**
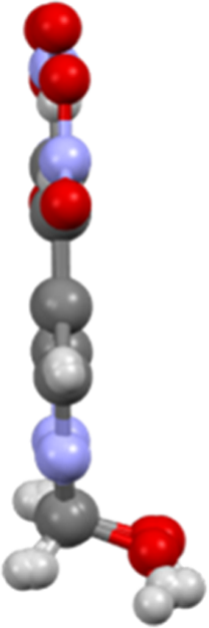
Superposition of the four structures.

The torsion angles of the 1-methanol substituent (N2–N1–C–O, Figure 4) and (N1–C–O–H) are 75.4/105.5°, 85.6/98.8°, 85.0/100.7°, and 86.4/101.3° for **2a**, **2b**, **2c**, and **2d**, respectively. The three nitro derivatives have average values of 85.7/100.3°, which differ from the unsubstituted derivative, 75.4/105.5°.

The nitro groups are almost coplanar with the benzene ring, with a mean value of 2.0° (lower and higher values of 1.75 and 2.65°, respectively). The O–H···N2 angles are 168.6, 149.3, 172.3, and 162.3° for **2a**, **2b**, **2c**, and **2d**, respectively (mean value of 163.1°). Note that an intermolecular O–H···N2 HB leading to dimers is preferred to the possible intramolecular HB of the monomer; this is due to angular strains in the HB that are much more favorable for the dimer.

### GIAO/B3LYP/6-311++G(d,p) Calculations of NMR Chemical Shifts and Some Coupling Constants of the 10 Isomers, **2*n*** to **3*n***, for *n* = a, b, c, d, e

This method has provided excellent results as long as there are no heavy atoms linked to the carbon atoms, *i.e*., HALA effects.^44,45^ Since the calculations afford absolute shieldings, σ ppm in the gas phase, it is necessary to use empirical equations to transform these data into chemical shifts, δ ppm in solution, equations that we have already established from a large set of data for ^1^H, ^13^C, and ^15^N NMR chemical shifts.^46^ The spin–spin coupling constants, SSCCs, do not need any transformation. The calculated values are reported in Table 1; the remaining coupling constants are given in the Supporting Information.

**Table 1:** GIAO/B3LYP/6-311++G(d,p)-Calculated ^1^H, ^13^C, and ^15^N NMR Chemical Shifts (δ in ppm) and Coupling Constants (*J* in Hz)

	2a	3a	2b	3b	2c	3c	2d	3d	2e	3e
nuclei	H	H	4-NO_2_	4-NO_2_	5-NO_2_	5-NO_2_	6-NO_2_	6-NO_2_	7-NO_2_	7-NO_2_
^1^H (ppm)										
H3	7.83	7.82	8.79	8.70	8.00	8.05	7.94	7.88	7.96	7.97
H4	7.65	7.62	NO_2_	NO_2_	8.70	8.78	7.64	7.61	7.87	7.92
H5	7.16	7.06	8.26	8.25	NO_2_	NO_2_	8.16	8.05	7.15	7.08
H6	7.37	7.26	7.35	7.29	8.40	8.23	NO_2_	NO_2_	8.17	8.49
H7	7.48	7.72	7.77	8.03	7.40	7.64	8.55	8.82	NO_2_	NO_2_
CH_2_	5.64	5.48	5.70	5.54	5.63	5.48	5.72	5.52	6.16	5.54
OH	1.59	2.15	1.74	2.29	1.79	2.33	1.80	2.28	1.42	2.59
^13^C (ppm)										
C3	134.0	120.8	135.4	125.3	136.7	125.8	134.1	122.0	134.7	122.9
C3a	126.6	123.7	120.3	116.7	125.5	121.6	129.4	125.8	131.2	126.7
C4	120.6	120.2	142.1	142.6	118.6	120.0	120.1	120.2	126.8	128.8
C5	120.8	122.3	119.3	121.9	144.5	145.3	117.0	117.6	119.5	120.0
C6	126.0	126.1	124.3	123.9	122.5	121.7	147.8	148.4	125.8	126.4
C7	108.4	119.0	115.7	128.0	107.8	118.6	106.3	117.6	138.8	139.8
C7a	139.7	150.8	140.7	151.2	141.2	151.3	138.0	148.7	131.4	142.6
CH_2_	72.4	76.7	73.0	77.0	72.8	77.0	72.7	77.2	76.5	77.2
^15^N (ppm)										
N1	–181.1	–93.3	–177.8	–91.4	–177.2	–91.4	–174.7	–81.9	–172.4	–87.7
N2	–60.7	–144.5	–50.1	–136.0	–52.4	–136.2	–45.4	–133.5	–48.4	–136.3
NO_2_			–15.8	–16.1	–17.3	–16.7	–16.8	–16.2	–14.1	–17.6
SSCC (Hz)										
^3^*J*_CH2OH_	–10.7	–9.3	–10.9	–9.4	–10.9	–9.5	–10.9	–9.5	–10.3	–9.3
^3^*J*_H4H5_	7.4	7.8	NO_2_	NO_2_	NO_2_	NO_2_	8.2	8.6	7.2	7.6
^3^*J*_H5H6_	6.4	6.2	7.3	7.2	NO_2_	NO_2_	NO_2_	NO_2_	7.3	7.2
^3^*J*_H6H7_	7.7	8.1	7.6	7.9	8.6	8.5	NO_2_	NO_2_	NO_2_	NO_2_
^4^*J*_H4H6_	0.4	0.5			1.5	1.5			0.3	0.5
^4^*J*_H5H7_	0.3	0.3	0.2	0.2			1.3	1.4		
^5^*J*_H3H7_	0.6	0.8	0.6	0.7	0.6	0.7	0.8	0.8	NO_2_	NO_2_

Obviously, the ^1^H and ^13^C chemical shifts of the aromatic indazole ring in Table 1 depend on the presence and position of the nitro group. In ^1^H NMR, in what concerns the methanol group, the OH proton shows some interesting variations but, since this signal is strongly dependent on the solvent, they are of little interest. Note, however, that the difference between the **2** and **3** isomers is about 0.5 ppm except in the **e** series where it reaches 1.2 ppm. The CH_2_ group appears between 5.5 and 5.6 ppm; only in compound **2e**, it resonates at 6.2 ppm due to the proximity of the 7-nitro group.

The ^13^C chemical shifts are very different in isomers **2** and **3**, a fact well known for other *N*-substituted indazoles.^47,48^ The signal of C3, a singlet or a doublet with a small coupling constant, is also a useful probe to determine the position of the CH_2_OH group: 135 ppm (**2**) and 123 ppm (**3**) in average. The ^15^N chemical shifts of N1 and N2 atoms are also very different for isomers **2** and **3**.

The SSCCs of the methanol group are slightly larger in the **2** series (−10.7 Hz) than in the **3** series (−9.4 Hz). The *ortho* SSCCs are normal and the W coupling,^49,50^^5^*J*_HH_ between protons H3 and H7,^15,17,47^ is calculated to be 0.7–0.8 Hz. The ^4^*J*_HH_ coupling constants are small (between 0.2 and 0.5 Hz) except when there is a nitro group in the central carbon [H–C–C(NO_2_)–C–H] where it attains 1.4–1.5 Hz; this effect of the nitro group has been reported for benzene derivatives.^51,52^

### Solid-State Nuclear Magnetic Resonance (SSNMR) Results (CPMAS Experiments)

The chemical shifts of the four compounds, **2a**–**2d**, whose X-ray structures have been determined in this work, are given in Table 2. As often happens in CPMAS, some signals are split, for instance, those of compound **2c**. For this compound, when comparing its chemical shifts δ_Exp_ (Table 2) with the calculated values δ_GIAO_ (Table 1), mean values have been used.

**Table 2:** ^13^C and ^15^N NMR Data of (1*H*-Indazol-1-yl)Methanol Derivatives in the Solid State (CPMAS)

nuclei	2a	2b (4-NO_2_)	2c (5-NO_2_)	2d (6-NO_2_)
C3	133.5	134.3	136.7	135.3
C3a	126.0	120.2	123.3	125.9
C4	119.4	138.8	118.9	124.0
			121.2	
C5	121.3	118.9	141.7	114.4
C6	123.0	126.3	121.2	145.6
			123.3	
C7	109.0	115.3	109.2	103.8
			109.7	
C7a	139.4	140.5	141.7	137.3
CH_2_	69.3	70.7	70.1	69.8
N1	–173.4	–171.7	–172.9	–173.4
N2	–68.2	–66.6	–63.3	–65.4
NO_2_		–6.4	–5.9	–6.6

Comparing the values in Tables 1 and 2 results in the following regression equations
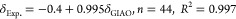
1
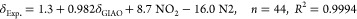
2

The largest residuals for ^15^N signals in the simple regression equation, eq 1, correspond to NO_2_ and N2. Including these effects as dummy variables, eq 2 was obtained with +9.7 and −16.0 ppm corrections for NO_2_ and N2, respectively. In any case, the ^15^N chemical shifts only can correspond to (indazol-1-yl)methanol isomers **2**.

### NMR in Solution

The experimental chemical shifts and SSCC in DMSO-*d*_6_ solution are reported in Table 3.

**Table 3:** ^1^H, ^13^C{^1^H}, and ^15^N NMR Data of (1*H*-Indazol-1-yl)Methanol Derivatives in DMSO-*d*_6_ Solution^a^

	2a^b^	2b	3b	2c	2d	1e–2*H*	2e	3e
nuclei	H	4-NO_2_	4-NO_2_	5-NO_2_	6-NO_2_	7-NO_2_	7-NO_2_	7-NO_2_
^1^H (ppm)								
H3	8.09	8.54	8.91	8.42	8.34	8.43	8.30	8.85
H4	7.72	NO_2_	NO_2_	8.83	8.03	8.33^e^	8.19	8.37
H5	7.17	8.20	8.21	NO_2_	7.99	7.37	7.38	7.28
H6	7.41	7.68	7.40	8.27	NO_2_	8.36^e^	8.28	8.48
H7	7.77	8.27	8.30	7.92	8.78	NO_2_	NO_2_	NO_2_
CH_2_	5.73	5.79	5.79	5.78	5.86		5.83	5.80
OH	6.68	6.95	7.50	6.94	6.93		6.59	7.48
^13^C (ppm)								
C3	134.2	132.1	124.8	136.4	134.0	136.2	135.5	120.4
C3a	126.0	116.6	113.9	123.3	127.4	127.6	*130.6*^g^	125.7
C4	121.6	140.6	143.0	118.9	122.2	130.4	128.8	126.7
C5	121.7	118.7	120.7	141.4	115.3	120.7	121.3	120.4
C6	127.0	126.0	123.8	121.0	145.9	124.0	124.8	125.7
C7	111.0	118.3	126.6	112.0	107.2	132.6^f^	*138.2*^g^	137.4
C7a	139.8	139.7	149.2	140.8	137.6	132.4^f^	*130.8*^g^	140.1
CH_2_	71.6	71.5	75.8	71.4	71.4		75.3	76.2
^15^N (ppm)								
N1	–180.8^b^	–173.7	–*90.2*	–174.3	–173.0		–*170.1*	–*86.5*
N2	–60.5^b^	–50.6	–*134.2*	–50.3	–45.2		–*47.7*	–*134.5*
NO_2_		–*15.6*	–*15.9*	–*17.1*	–*16.6*		–*13.9*	–*17.4*
SSCC (Hz)								
^3^*J*_CH2OH_	–7.3^c^	–7.5^c^	–8.0^c^	–7.5^c^	–7.4^c^		–7.7^c^	–7.9^c^
^3^*J*_H4H5_	8.5	NO_2_	NO_2_	NO_2_	8.8	7.9	7.9	8.2
^3^*J*_H5H6_	7.5	7.3	7.7	NO_2_	NO_2_	7.9	7.9	7.5
^3^*J*_H6H7_	8.0	7.6	8.4	9.2	NO_2_	7.9	NO_2_	NO_2_
^4^*J*_H4H6_	1.0	NO_2_	NO_2_	2.2	NO_2_	0.9	1.0	1.0
^4^*J*_H5H7_	0.9	0.9	d	NO_2_	1.5	NO_2_	NO_2_	NO_2_
^5^*J*_H3H7_	0.8^d^	0.9	0.0	1.0	0.0	NO_2_	NO_2_	NO_2_

a Italic type: predicted values.

b From ref (17): ^3^*J*_N1H3_ = 7.9, ^2^*J*_N2H3_ = 13.0, ^3^*J*_N2CH2_ = 2.7 Hz;^17^ calculated, this work, ^3^*J*_N1H3_ = 5.5, ^2^*J*_N2H3_ = 9.2, ^3^*J*_N2CH2_ = 2.0 Hz.

c The minus sign was assigned from the calculations.

d Not measured.

e Assignment based on coupling constants (Figure 5).

f From ref (47).

g Not observed.

In the ^1^H NMR spectrum of the reaction crude between protonated 7-nitroindazole (**1eH**^**+**^) and formaldehyde, we observed three triplets of the same intensity, 1:1:1, and approximately the same coupling constant, 8.0, 7.9, and 7.9 Hz for the 7.48, 7.38, and 7.28 ppm signals, respectively (Figure 5). By analogy to other compounds, these multiplets should correspond to three H5 protons coupled with H4 and H6.

**Figure 5:**
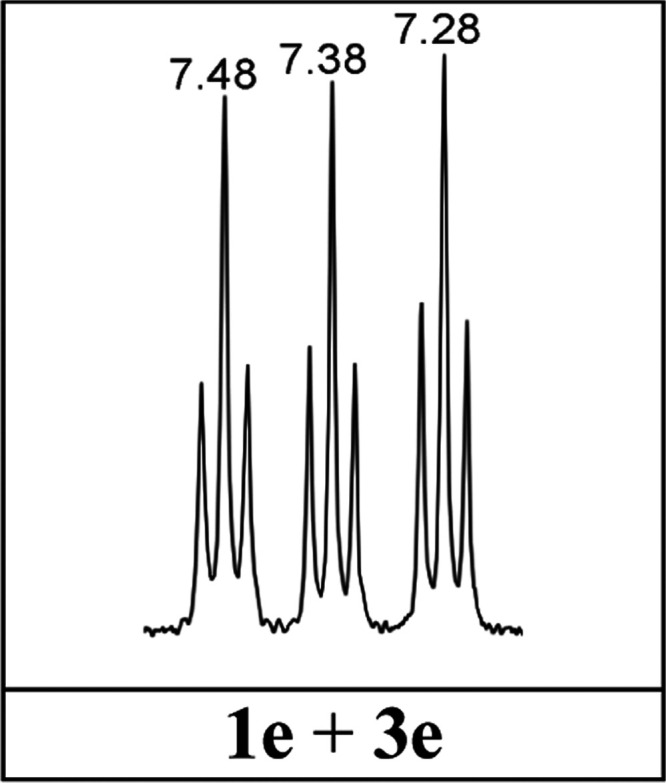
7.2–7.5 ppm region of the ^1^H NMR in DMSO-*d*_6_ at 400 MHz of the reaction product between **1eH**^**+**^ and formaldehyde.

**Figure 6:**
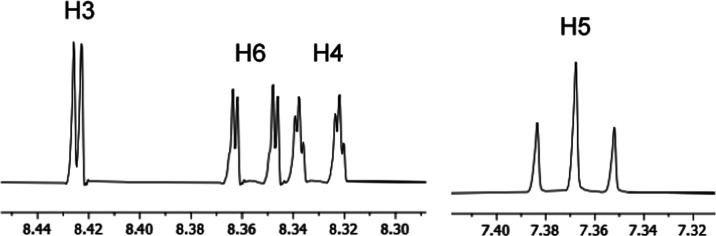
^1^H NMR spectrum (8.30–8.44 and 7.32–7.40 ppm regions) of **1e** in DMSO-*d*_6_ at 500 MHz.

**Figure 7:**
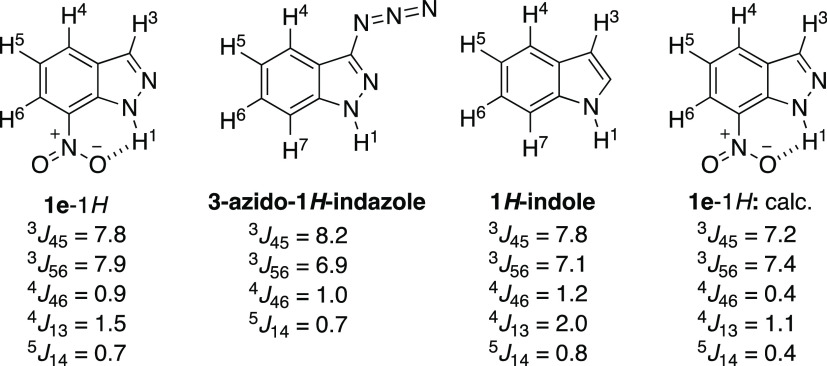
^1^H–^1^H SSCCs of some indazoles and indoles first-order analyzed.

When the spectrum of **1e** was recorded at 500 MHz in DMSO-*d*_6_, its ^1^H NMR spectrum shows some very unusual ^1^H–^1^H coupling constants (Figures 6 and 7).

Those measured in Figure 6 are reported on the left side in Figure 7. Because prototropy couplings with the NH are very rare and have been observed only on 3-azido-1*H*-indazole, we assigned this to the azido group blocking the tautomerism of indazole.^53^ Indazole tautomer 1*H* resembles 1*H*-indole where H1 is coupled, besides to H2, to H3 and H4.^54^ The calculated SSCCs of **1e**–1*H* are given on the right side in Figure 7. The strong HB between H1 and one oxygen atom of the nitro group prevents the prototropy and allows the SSCCs with H1 to be observed. Note that the ^1^H NMR spectrum of **1e**–1*H* has been described several times but these small couplings were never reported.^47,55,56^

**Figure 8:**
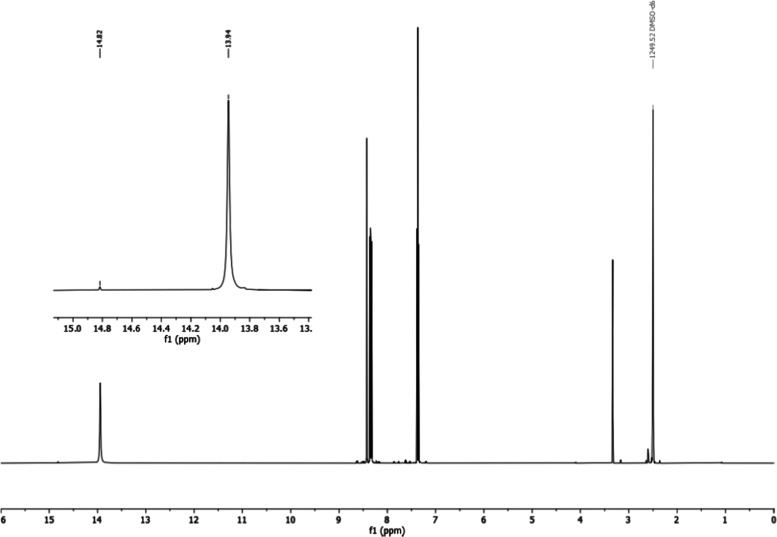
^1^H NMR complete spectrum (0–16 ppm) of indazole **1e** at 500 MHz in DMSO-*d*_6_. The inset corresponds to the zone of NH protons.

The spectrum of **1e**–1*H* in the region of the NH proton (DMSO-*d*_6_ at 500 MHz) shows two signals, a large one (13.95 ppm, 94%) and a small one (14.83, 6%), as shown in Figure 8. We assign the small signal to tautomer 1*H* by analogy with the GIAO calculations, 10.14 and 10.81 ppm. The differences are 0.88 ppm, experimental, and 0.67 ppm, calculated, and the shift produced by the solvent is about 3.9 ppm. The other signals of the minor tautomer are not observed except that of H6 that appears at 8.58 ppm (^3^*J*_56_ = 7.9, ^4^*J*_46_ = 0.9 Hz) due to the spinning side bands and the big signals of the **1e**–2*H* tautomer. An equation relies on experimental and calculated values if the effect of DMSO on NH signal is taken into account: Exp. = (0.95 ± 0.16) Calc. + (3.8 ± 0.4) NH, *n* = 7, *R*^2^ = 0.998.

**Figure 9:**
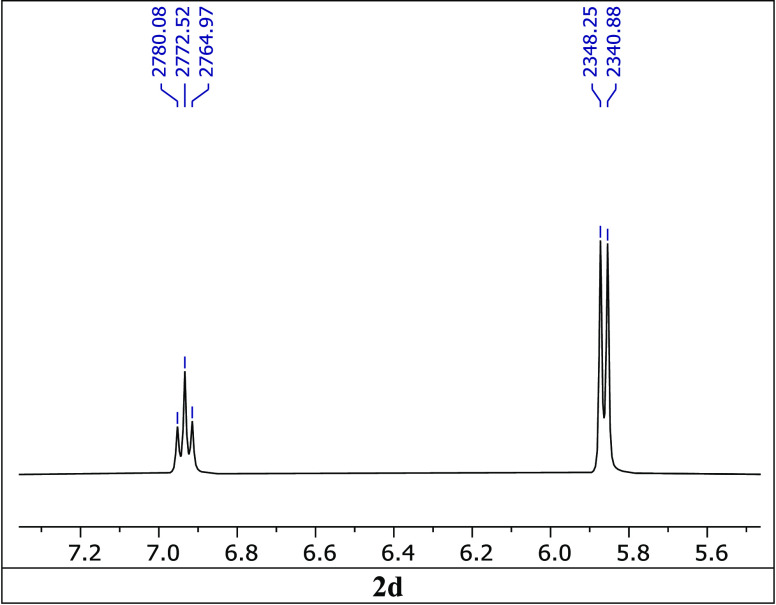
Appearance of the CH_2_OH group in ^1^H NMR spectra in DMSO-*d*_6_ at 400 MHz of the derivative of 6-nitro-1*H*-indazole **2d**; ^3^*J*_CH2OH_ = 7.5 Hz.

Actually, the triplets of Figure 5 correspond to a 1:1 mixture of **1e**–1*H* (H5 at 7.38 ppm) and **3e** (H5 at 7.28 and OH at 7.48 ppm). The A_2_X system of the methanol part appears well resolved in some ^1^H NMR spectra in DMSO-*d*_6_ (^3^*J*_HH_ ∼7.5 Hz) (Figure 9), which is not always the case for common alcohols.

Note that the ^3^*J*_H4H5_ and ^3^*J*_H5H6_ are identical for **2e** and different for **3e**: this is characteristic of 1- and 2-substituted indazoles.^47^ COSY experiments correlate OH → CH_2_ → H3 → H4 → H5 → H6 in the case of **2e** and **3e** with some exceptions when signals are under the larger signals of **1e**.

To compare the experimental values of Table 3 (DMSO-*d*_6_ solution) with the calculated chemical shifts in Table 1 (gas phase), we have used simple regressions between both values, except in two cases. First, in ^1^H NMR chemical shifts, the OH signal is systematically underestimated because our equations relating absolute shieldings in the gas phase to chemical shifts in solution correct general solvent effects and not the hydrogen bond between the OH and DMSO.^57,58^ Second, in the ^3^*J*_CH2OH_ SSCC, the same happens for the same reason. To correct these deviations, an additional variable (1 if OH was present and 0 if it was absent) was added.^59−61^ In any case, the intercept was not significant and the regressions were repeated imposing intercept = 0, but the squared correlation coefficient, *R*^2^, was that of the regression with the intercept because imposing the intercept to be 0 increased considerably the *R*^2^ value (Table 4).

**Table 4:** Results of the Slopes of the Five Regression Equations: Experimental Values = a Calculated Values + b OH Protons

eq		no. of points	a calc.	b OH	*R*^2^	RMS error
1	^1^H chemical shifts	44	(1.02 ± 0.01)	(5.1 ± 0.1)	0.947	0.23 ppm
2	^13^C chemical shifts	53	(0.996 ± 0.002)		0.994	1.7 ppm
3	^15^N chemical shifts	6	(0.986 ± 0.005)		1.000	1.7 ppm
4	^1^H, ^13^C, and ^15^N	103	(0.994 ± 0.001)	(5.1 ± 0.5)	1.000	1.2 ppm
5	^1^H–^1^H SSCC	25	(1.08 ± 0.02)	–(3.5 ± 0.3)	0.962	0.6 Hz

The slopes are close to 1.0; the experimental ^1^H chemical shifts of the OH are 5.1 ppm higher on average than the calculated ones, while the SSCCs involving the OH group, ^3^*J*_CH2OH_, are 3.5 Hz lower.

**Figure 10:**
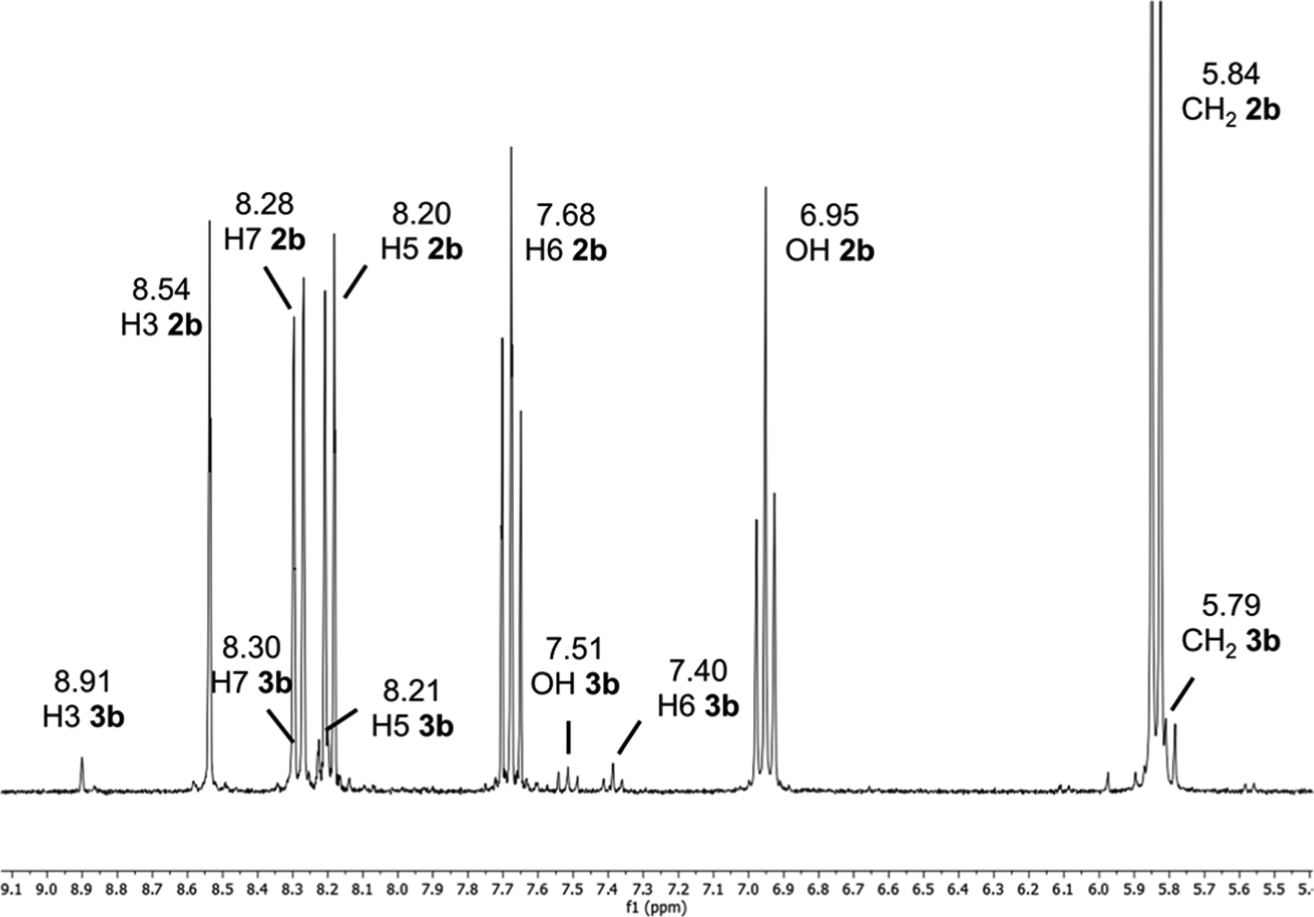
^1^H NMR spectrum of crude of **2b** in DMSO-*d*_6_ solution containing 95% of **2b** and 5% of **3b**.

**Figure 11:**
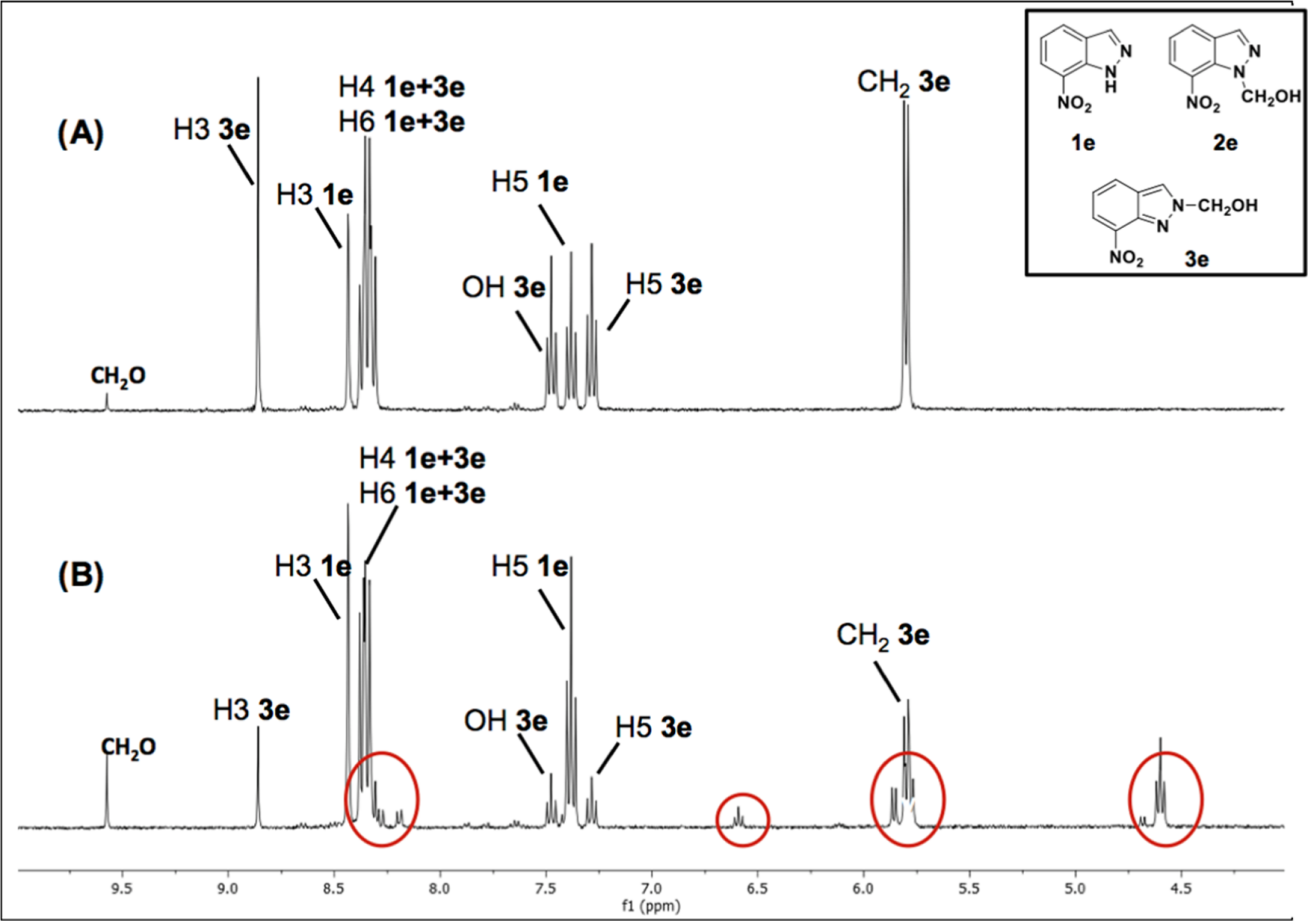
Top: ^1^H NMR spectrum of the crude of **3e** in DMSO-*d*_6_ solution freshly prepared; bottom: ^1^H NMR spectrum of the crude of **3e** in DMSO-*d*_6_ solution after a week in the NMR tube (both at 400 MHz). The signals in the 4.5–5.0 ppm region and a doublet in the 5.5–6.0 region are not indazole derivatives but most probably formaldehyde short polymers.

The most interesting ^1^H NMR spectra are those of the crude of **2b** (Figure 10, neutral solid in DMSO-*d*_6_ solution) and those of the crude of **3e** freshly prepared (Figure 11, filtered solid in DMSO-*d*_6_ solution) and after a week in the NMR tube.

After crystallization (see the Supporting Information), the 5% amount of **3b** has been eliminated.

Figure 11 (top) corresponds to a mixture of starting 7-nitro-1*H*-indazole **1e** and its 2-methanol derivative, **3e**. After a week, Figure 11 (bottom), **3e** (neutral) has decomposed into **1e** and a small quantity of another compound that we have identified as isomer **2e**.

**Scheme 4:**
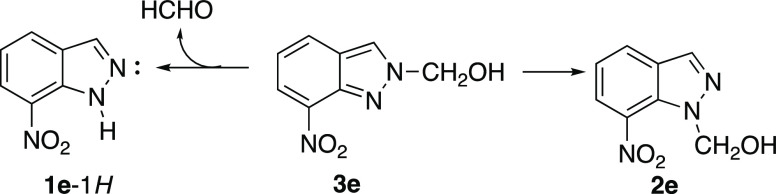
Decomposition of **3e** into **1e** (Major) and **2e** (Minor)

This behavior (Scheme 4) will be explained by the theoretical calculations in the following section.

### Reaction Mechanism: DFT Calculations

According to the conclusions of the NMR analyses, the five indazoles regroup in three cases: obtaining in HCl (aq) 1-methanol derivatives (unsubstituted **a**, 5-nitro **c**, and 6-nitro **d**), obtaining a mixture of 1- and 2-methanol derivatives (95/5 4-nitro **b**), and obtaining a 2-methanol derivative **3e** that in DMSO-*d*_6_ decomposes and partly isomerizes into 1-methanol derivative **2e** (7-nitro **e**).

**Figure 12:**
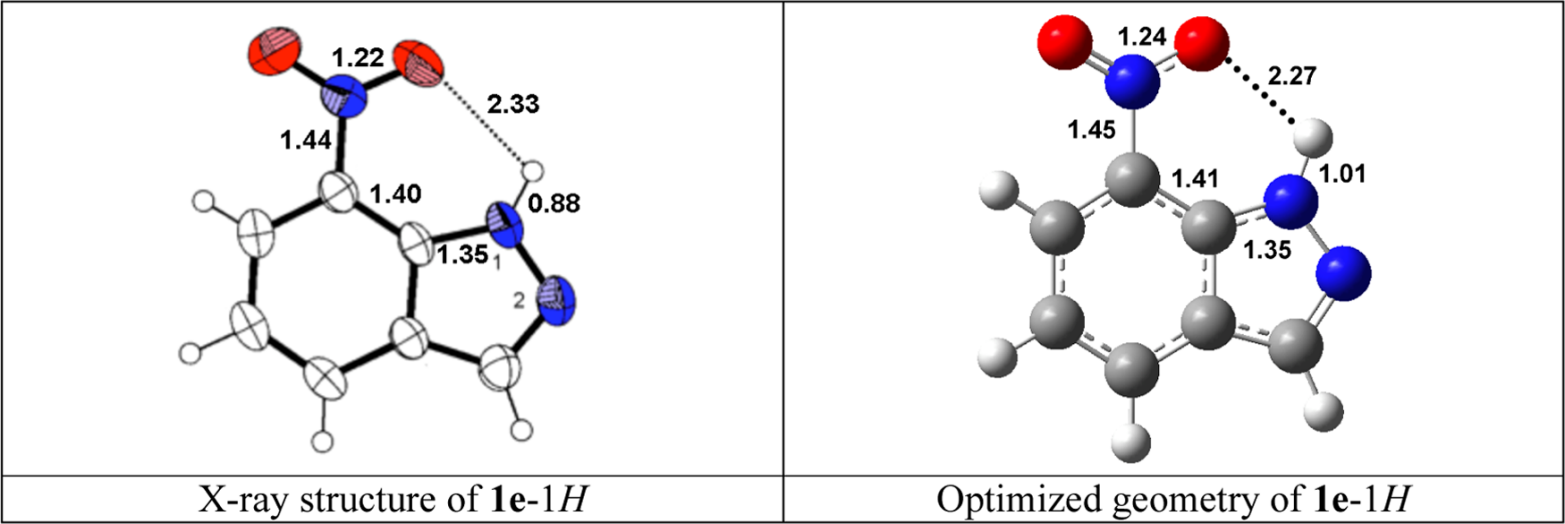
Experimental (adapted) and calculated structures of **1e**–1*H*.

In Table 5 are the energies corresponding to the equilibria between **2** and **3** isomers; in all cases, the 1-CH_2_OH isomer is more stable than the 2-CH_2_OH one, similarly to what happens for the N*H* tautomers; note that the values are similar except in the case of the **e** series where the difference is much larger, about 3.5 times. This is due to a strong hydrogen bond between the N–*H* and O=N–O^–^ bonds (Figure 12) that disappears in the N-1-substituted derivative, confirming the NMR discussion about the HB (Figures 6 and 7). An analogous HB is present in 3,7-dinitro-1*H*-indazole.^62^ Protonation on N2 must reinforce the strength of the HB, being now N1^(+)^–H···O. The X-ray distances of the atoms involved in the hydrogen bond are the mean of two very similar structures;^63,64^ the only difference between the experimental and the calculated geometry lies in the N–H distance, which is underestimated by X-ray crystallography;^65^ this in turn affects the O···H distance.

**Table 5:** Energies (kJ·mol^–1^) Corresponding to Scheme 3 Calculated at the DLPNO/CCSD(T)/def2-TZVP//B3LYP/6-311++G(d,p) Level^a^

	parent	4-NO_2_	5-NO_2_	6-NO_2_	7-NO_2_
	a	b	c	d	e
2-isomer 1-CH_2_OH (reference)	0.0	0.0	0.0	0.0	0.0
**3** isomer 2-CH_2_OH	18.6	12.6	16.6	16.9	13.3
**1**-1*H* (reference)	0.0	0.0	0.0	0.0	0.0
**1**-2*H*	18.3	10.5	16.3	16.6	42.9

a The values are given relative to reference compounds.

The differences decrease in the order **a** > **d** > **c** > **e** > **b**. The formation of **2e** from **3e** is not related to the ΔΔE value (13.7 kJ·mol^–1^) but simply that it is only in the **e** series that 2-isomer **3** is formed since in all cases the **2** isomers are more stable than the **3** isomers.

**Figure 13:**
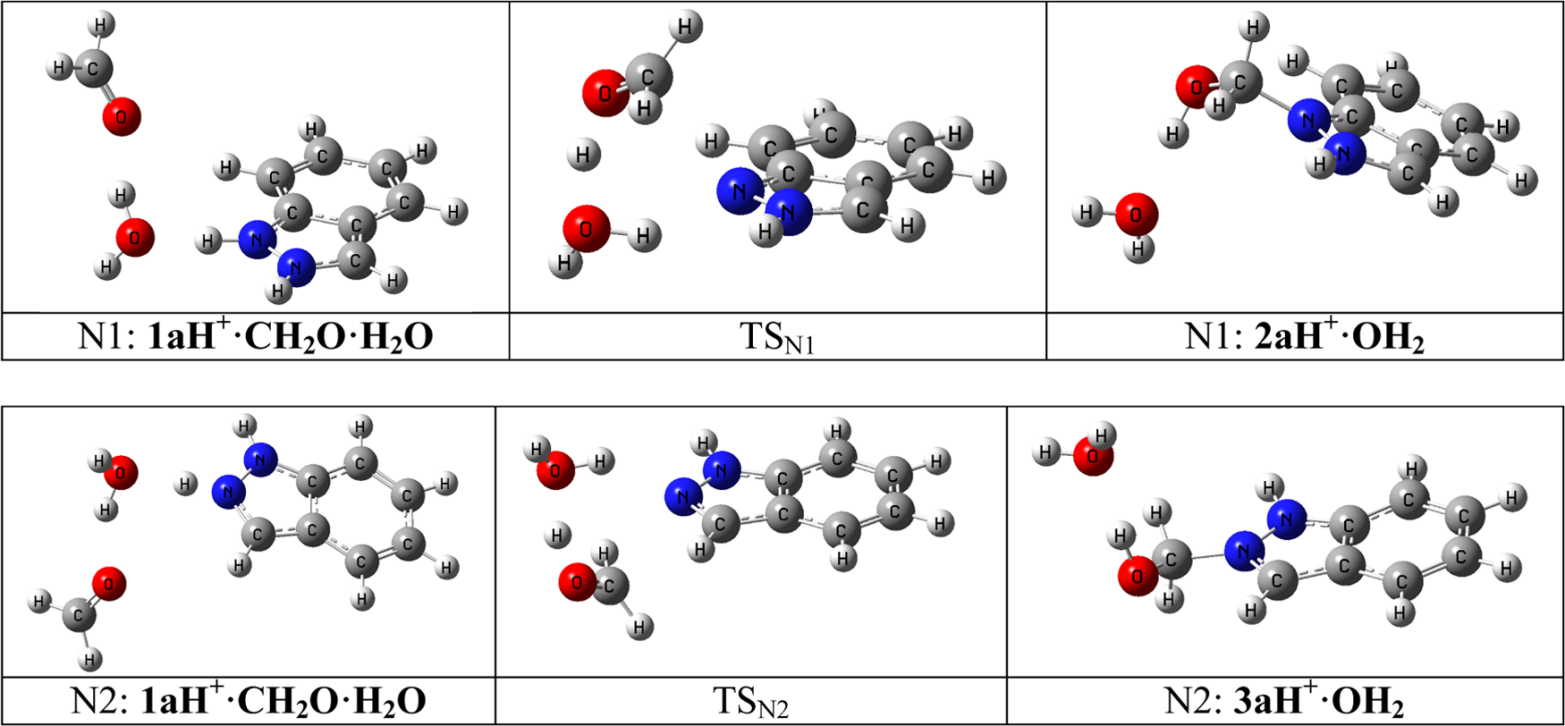
Mechanisms corresponding to Scheme 4.

**Scheme 5:**
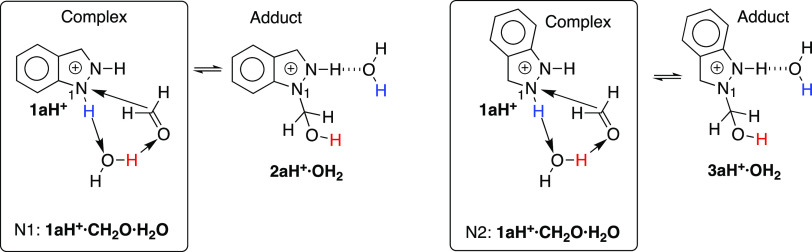
Proposed Mechanism Illustrated for **a** Series The indazolium rings are rotated in the right part of the figure to keep formaldehyde, water molecules, and the hydroxymethyl group at the same position.

The mechanism for the unsubstituted indazole, **a** series, is represented in a simplified way in Scheme 5 and in a more realistic way, including TSs and IRCs (see the Supporting Information), in Figure 13.

**Scheme 6:**
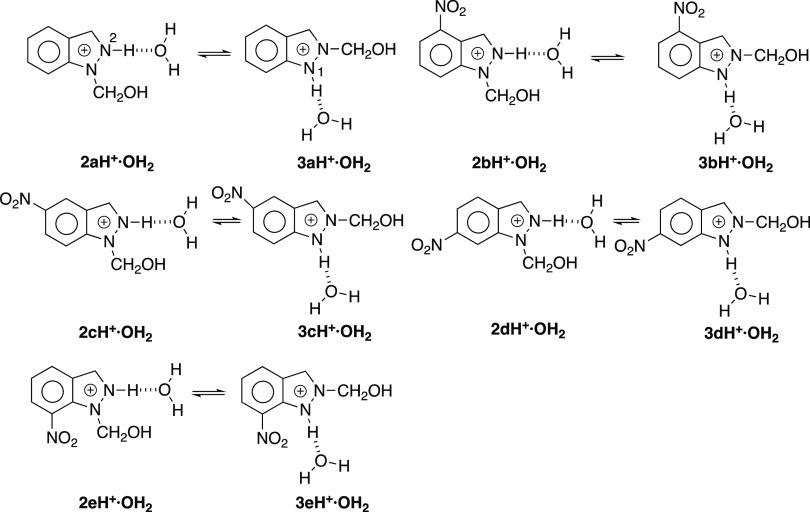
Relative Stability of Water-Solvated 1- and 2-Methanol Indazolium Salts

The differences in stability of the five pairs of indazolium salts are reported in Scheme 6 and Table 6. In this table, N1 and N2 indicate the position of the CH_2_OH group and complex, TS, and adduct corresponds to the complex, transitions state, and adduct in Scheme 5.

**Table 6:** Energies (kJ·mol^–1^) Corresponding to Scheme 6; x = **a**, **b**, **c**, **d**, **e**^a^

DLPNO + PCM						
water	parent a	4-NO_2_ b	5-NO_2_ c	6-NO_2_ d	7-NO_2_ e	mean a–d
N1–complex **1xH**^**+**^**·CH**_**2**_**O·H**_**2**_**O**	0.0	0.0	0.0	0.0	0.0^b^	0.0
N1–TS	82.2	67.9	69.7	70.1	b	72.5
N1–adduct **2xH**^**+**^**·OH**_**2**_	–54.6	–49.3	–48.9	–46.9	–29.7	–49.9
N2–complex **1xH**^**+**^**·CH**_**2**_**O·H**_**2**_**O**	–2.3	–2.7	–1.4	0.1	0.0	–1.6
N2–TS	74.6	57.8	60.2	61.0	61.8	63.4
N2–adduct **3xH**^**+**^**·OH**_**2**_	–38.1	–55.9	–54.9	–53.1	–52.3	–50.5
N2–adduct – N1–adduct	16.5	–6.6	–6.0	–6.2	–22.6	–0.6

a The differences between the N-complexes and between the N-adducts are also reported. The energies correspond to DLPNO/CCSD(T) single-point calculations including the contribution of PCM–water. The gas phase values are reported in the Supporting Information.

b See the comment below.

Although there are some differences in Table 6, the behavior of the **a**, **b**, **c**, and **d** series is similar (see mean **a**–**d**): in 1-series, a barrier of about 72 kJ·mol^–1^, the adduct being more stable than the complex by about 52 kJ·mol^–1^; in 2-series, a barrier of about 63 kJ·mol^–1^, the adduct being more stable than the complex by about 2 kJ·mol^–1^. The differences between both series, bottom of Table 6, are very small, ±1.6 kJ·mol^–1^. The **e** series is very different; when reacting by N2–H, far from the nitro group, the behavior is near identical, 52.3/50.5 and 61.8/63.4 kJ·mol^–1^, but when reacting N1–H, hydrogen-bonded to the nitro group, the complex spontaneously isomerizes to the complex formed by N2–H, which leads to **3eH**^**+**^**·OH**_**2**_ (Scheme 6). This explains why this isomer reacts differently from all of the other indazoles.

## Conclusions

We have demonstrated that the reaction of N*H*-indazoles with formaldehyde, previously reported to yield exclusively 1-CH_2_OH derivatives, gives rise in some cases to 2-CH_2_OH indazoles, as found for 4-nitro-1*H*-indazole (**1b)** and 7-nitro-1*H*-indazole (**1e**). This result is important when hydroxymethyl-indazoles are used as intermediates without isolating them.

The structure, tautomerism, and reactivity of **1e** are of interest because of its unique ability to inhibit both MAO-B and nNOS, two biologically important enzyme systems. Furthermore, its general use as an investigative drug to study the inhibition of nNOS makes the structural study of this molecule very relevant.^12,13^ This compound is the first reported indazole where both tautomers have been observed and the second in which spin–spin coupling constants with H1 have been observed observed and determined.

The X-ray structures of four 1-CH_2_OH indazoles, (1*H*-indazol-1-yl)methanol (**2a**), (4-nitro-1*H*-indazol-1-yl)methanol (**2b**), (5-nitro-1*H*-indazol-1-yl)methanol (**2c**), and (6-nitro-1*H*-indazol-1-yl)methanol (**2d**), were solved, offering a solid ground for NMR spectra in the solid state, and, in turn, these spectra were used for assigning the NMR spectra in DMSO-*d*_6_ solution.

Theoretical calculations at the B3LYP/6-311++G(d,p) level have been used to understand the reaction mechanism and, in particular, the different behavior of **1e**. Besides, GIAO calculations based on the optimized geometries proved an excellent tool to identify indazole isomers.

## Experimental Section

### General Methods

Acetonitrile, nitromethane, dioxane, heptane, hydrochloric acid, and indazoles were purchased from Merck without further purification. Melting points were determined by a capillary method in a Metler Toledo scientific melting point apparatus (MP760) at a heating rate of 1 °C/min. A PerkinElmer Spectrum Two, fitted with a diamond single-bounce ATR, was used to collect the IR spectra at 4 cm^–1^ spectral resolution with four co-adds (*i.e.*, the number of averaged replicate spectra). The compound was pressed on the diamond crystal and measured directly without any further sample. For ^1^H and ^13^C NMR spectra, see below. Reactions heated under microwave irradiation were carried out for 60 min at 80 °C in sealed reaction vessels of a Biotage Initiatior microwave oven reactor (frequency: 2045 GHz). Analytical HPLC was performed with a SunFire C18, 3.5 μm column (4.6 mm × 50 mm). Mobile phase A was CH_3_CN + 0.08% formic acid, and mobile phase B was H_2_O + 0.05% formic acid. The gradient was from 10 to 95% of acetonitrile. UV diode array detection was carried out from 190 to 440 nm.

#### General Synthesis of Indazolyl-*N*-Methanol Derivatives

All of the indazolyl-*N*-methanol derivatives were synthesized using the method reported in the literature^15^ with some differences: the reactions were stirred overnight at room temperature to ensure that all final products were obtained and no crystallization from water was used (because the starting products were obtained in this solvent). Indazoles (42 mmol) are suspended in 30 mL of 30% hydrochloric acid and then 3.85 mL of a 30% aqueous solution of formaldehyde (42 mmol) was added. After 1 h, 30 mL of water was added and the mixture was kept overnight at room temperature. The precipitate was collected by filtration to give a solid. To obtain crystals, the solid was suspended in the solvent specified for each compound and heated and the solution was filtered to remove undesirable products. By slow cooling, crystals were precipitated and removed from the solvent to give the desired compound. Crystallization solvents were specified for each compound. Compound **3b** (4-NO_2_) was obtained as a minor product and could not be isolated and was only observed by NMR. Compound **2e** and **3e** (7-NO_2_) could not be isolated due to decomposition but could be detected by NMR.

##### (1H-indazol-1-yl)methanol (**2a**)

Yield 98% (6.15 g), white crystalline solid; mp: 110.5–111.5 °C (heptane); ^1^H NMR (400 MHz, DMSO-*d*_6_): δ 8.09 (s, 1H, H3), 7.77 (d, 1H, *J* = 8.0 Hz, H7), 7.72 (d, 1H, *J* = 8.5 Hz, H4), 7.41 (t, 1H, *J* = 8.0 Hz, H6), 7.17 (t, 1H, *J* = 8.5 Hz, H5), 6.68 (t, 1H, *J* = 7.3 Hz, OH), 5.73 (d, 2H, *J* = 7.3 Hz, CH_2_) ppm. ^13^C{^1^H} NMR (100 MHz, DMSO-*d*_6_): δ 139.8 (C7a), 134.2 (C3), 127.0 (C6), 126.0 (C3a), 121.7 (C5), 121.6 (C4), 110.1 (C7), 71.6 (CH_2_) ppm. MS (ES^+^): *m/z*: calcd for [M + H]^+^ C_8_H_9_N_2_O: 149.07, found: 148.94 (3.168 min).

##### (4-nitro-1H-indazol-1-yl)methanol (**2b**)

Yield 92% (7.52 g), yellow crystalline solid; mp: 168–170 °C (acetonitrile); ^1^H NMR (400 MHz, DMSO-*d*_6_): δ 8.54 (s, 1H, H3), 8.27 (d, 1H, *J* = 7.6 Hz, H7), 8.20 (d, 1H, *J* = 7.3 Hz, H5), 7.68 (t, 1H, *J* = 7.3 Hz, H6), 6.95 (t, 1H, *J* = 7.5 Hz, OH), 5.79 (d, 2H, *J* = 7.5 Hz, CH_2_) ppm. ^13^C{^1^H} NMR (100 MHz, DMSO-*d*_6_): δ 140.6 (C4), 139.7 (C7a), 132.1 (C3), 126.0 (C6), 118.7 (C5), 118.3 (C7), 116.6 (C3a), 71.5 (CH_2_) ppm. MS (ES^+^): calcd for [M + H]^+^ C_8_H_8_N_3_O_3_: 194.06, found: 194.05 (4.162 min).

##### (4-nitro-2H-indazol-2-yl)methanol (**3b**)

(4-NO_2_, minor product, NMR tube). Mp: ^1^H NMR (400 MHz, DMSO-*d*_6_): δ 8.91 (s, 1H, H3), 8.30 (d, 1H, *J* = 8.4 Hz, H7), 8.21 (d, 1H, *J* = 7.7 Hz, H5), 7.50 (t, 1H, *J* = 8 Hz, OH), 7.40 (t, 1H, *J* = 8.4 Hz, H6), 5.79 (d, 2H, *J* = 8.0 Hz, CH_2_) ppm. ^13^C{^1^H} NMR (100 MHz, DMSO-*d*_6_): δ 149.2 (C7a), 143.0 (C4), 126.6 (C7), 124.8 (C3), 123.8 (C6), 120.7 (C5), 113.9 (C3a), 75.8 (CH_2_) ppm.

##### (5-nitro-1H-indazol-1-yl)methanol (**2c**)

Yield 96% (7.85 g), light yellow crystalline solid; mp: 155.5–156.5 (dioxane) °C; ^1^H NMR (400 MHz, DMSO-*d*_6_): δ 8.83 (s, 1H, H4), 8.42 (s, 1H, H3), 8.27 (d, 1H, *J* = 9.2, H6), 7.92 (d, 1H, *J* = 9.2 Hz, H7), 6.94 (t, 1H, *J* = 7.5 Hz, OH), 5.78 (d, 2H, *J* = 7.5 Hz, CH_2_) ppm. ^13^C{^1^H} NMR (100 MHz, DMSO-*d*_6_): δ 141.4 (C5), 140.8 (C7a), 136.4 (C3), 123.3 (C3a), 121.0 (C6), 118.9 (C4), 112.0 (C7), 71.4 (CH_2_) ppm. MS (ES^+^): calcd for [M + H]^+^ C_8_H_8_N_3_O_3_: 194.06, found: 193.95 (4.250 min).

##### (6-nitro-1H-indazol-1-yl)methanol (**2d**)

Yield 94% (7.69 g), brown crystalline solid; mp: 142.5–143.5 °C (nitromethane); ^1^H NMR (400 MHz, DMSO-*d*_6_): δ 8.78 (s, 1H, H7), 8.34 (s, 1H, H3), 8.03 (d, 1H, *J* = 8.8 Hz, H4), 7.99 (dd, 1H, *J* = 8.8, 1.5 Hz, H5), 6.93 (t, 1H, *J* = 7.4 Hz, OH), 5.86 (d, 2H, *J* = 7.4 Hz, CH_2_) ppm. ^13^C{^1^H} NMR (100 MHz, DMSO-*d*_6_): δ 145.9 (C6), 137.6 (C7a), 134.0 (C3), 127.4 (C3a), 122.2 (C4), 115.3 (C5), 107.2 (C7), 71.4 (CH_2_) ppm. MS (ES^+^): calcd for [M + H]^+^ C_8_H_8_N_3_O_3_: 194.06, found: 193.98 (4.520 min).

##### (7-nitro-1H-indazol-1-yl)methanol (**2e**)

(NMR tube). Yellow solid. ^1^H NMR (500 MHz, DMSO-*d*_6_): δ 8.30 (s, 1H, H3), 8.28 (d, 1H, H6), 8.19 (d, 1H, *J* = 7.9 Hz, H4), 7.38 (t, 1H, *J* = 7.9 Hz, H5), 6.59 (t, 1H, *J* = 7.7 Hz, OH), 5.83 (d, 2H, *J* = 7.7 Hz, CH_2_) ppm. ^13^C{^1^H} NMR (125 MHz, DMSO-*d*_*6*_): δ 138.2 (C7), 135.5 (C3), 130.8 (C7a), 130.6 (C3a), 128.8 (C4), 124.8 (C6), 121.3 (C5), 75.3 (CH_2_) ppm.

##### (7-nitro-2H-indazol-2-yl)methanol (**3e**)

(NMR tube). ^1^H NMR (500 MHz, DMSO-*d*_*6*_): δ 8.85 (s, 1H, H3), 8.48 (d, 1H, H6), 8.37 (d, 1H, *J* = 8.2 Hz, H4), 7.28 (t, 1H, *J* = 7.9, 2 Hz, H5), 7.48 (t, 1H, *J* = 7.9 Hz, OH), 5.80 (d, 2H, *J* = 7.9 Hz, CH_2_) ppm. ^13^C{^1^H} NMR (125 MHz, DMSO-*d*_*6*_): δ 140.1 (C7a), 137.4 (C7), 126.7 (C4), 125.7 (C3a), 125.7 (C6), 120.4 (C3), 120.4 (C5), 76.2 (CH_2_) ppm.

### X-ray Crystallographic Methods

Colorless parallel pipe-shaped crystals of **2a**, **2b**, **2c**, **and 2d** were selected under a polarizing optical microscope. Data were collected at 250 K on a Bruker X8 four circle kappa-diffractometer equipped with a Cu Incoatec microsource operating at 50 W power (50 kV, 1.0 mA) to generated Cu Kα radiation (λ = 1.54178 Å) and a Bruker VANTEC 500 area detector (microgap technology). Diffraction data were collected exploring over a hemisphere of the reciprocal space in a combination of φ and ω scans to reach a resolution of around 0.85 Å, using the Bruker APEX21 software suite (each exposure, depending on ω, was of 10, 30, or 60 s covering 1° in ω or φ). Unit cell dimensions were determined by a least-squares fit of reflections with *I* > 2 σ(*I*). Data were integrated and scaled using the SAINTplus program.^66^ A semiempirical absorption and scale correction based on equivalent reflection was carried out using SADABS.^67^ Space group determination was carried out using XPREP.^69^ The structure was solved by direct methods using SHELXT,^68^ showing all no-hydrogen atoms. Additional cycles of refinement and electron difference maps show the rest of hydrogen atoms. The hydrogen atoms were refined riding on the coordinates of the respectively C-bonded atoms. The OH hydrogen atoms were allowed to ride on the O atom and rotate about the C–O bond. All calculations were performed using APEX3 software for data collection and OLEX2-1.3^69^ and SHELXTL^69^ to resolve and refine the structure. Mercury^70^ was used for structural figures and supramolecular packing studies. The final structure was examined and tested using PLATON.^71^ A summary of the main crystallographic data is shown in Table S1, and ORTEP representations of the asymmetric units are shown in Figure S19a–d.

### NMR Spectroscopy

Solution spectra were recorded either on three spectrometers, a Bruker DRX-400 (9.4 Tesla, 400.13 MHz for ^1^H, 100.62 MHz for ^13^C and 40.54 MHz for ^15^N), a Bruker Avance III HD-400 (^1^H 399.86 MHz, ^13^C 100.55 MHz), and a Varian SYSTEM 500 NMR (^1^H 499.81 MHz, ^13^C 125.69 MHz) equipped with a 5 mm HCN cold probe. Chemical shifts (δ in ppm) are given from the internal solvent: DMSO**-***d*_*6*_, 2.49 for ^1^H and 39.5 for ^13^C. Nitromethane was used as an external reference for ^15^N. For ^13^C, WALTZ-16 was used for broadband proton decoupling and ^15^N NMR spectra were acquired using 2D (^1^H–^15^N) gradient-selected heteronuclear multiple bond correlation by means of standard pulse sequences and in absolute mode.

Typical parameters: for ^1^H spectra, spectral width of 5200 Hz, acquisition time of 6.3 s, digital resolution of 0.41 Hz per point, and pulse width of 7.6 μs at an attenuation level of −1 dB; for ^13^C spectra, spectral width of 20.2 kHz, acquisition time of 1.6 s, digital resolution of 1.12 Hz per point, pulse width of 14.5 μs at an attenuation level of −4 dB, and relaxation delay of 2 s; the FIDS were multiplied by an exponential weighting (lb = 1 Hz) before Fourier transformation.

Solid-state ^13^C (100.73 MHz) and ^15^N (40.60 MHz) CPMAS NMR spectra were obtained on a Bruker WB 400 spectrometer at 300 K using a 4 mm DVT probehead. Samples were carefully packed in a 4 mm diameter cylindrical zirconia rotor with Kel-F end caps. ^13^C spectra were originally referenced to a glycine sample, and then the chemical shifts were recalculated to the Me_4_Si [for carbonyl atom (glycine) δ = 176.1 ppm] and ^15^N spectra to ^15^NH_4_Cl and then converted to the nitromethane scale using the following relationship: δ^15^N (nitromethane) = δ^15^N (ammonium chloride) −338.1 ppm. Typical acquisition parameters for ^13^C CPMAS are as follows: 3.2 μs 90° ^1^H pulses and decoupling SPINAL 64^72^ sequence spectral width, 40 kHz; recycle delay, 5–120 s; acquisition time, 30 ms; contact time, 2–4 ms; and spin rate, 12 kHz. Typical acquisition parameters for ^15^N CPMAS are as follows: 3.2 μs ^1^H 90° pulses (SPINAL 64) spectral width, 40 kHz; recycle delay, 5–120 s; acquisition time, 35 ms; contact time, 7–9 ms; and spin rate, 6 kHz.

Abbreviations for multiplicity are as follows: d indicates doublet, t indicates triplet, m indicates multiplet, bs indicates broad singlet, bd indicates broad doublet, dd indicates double doublet, dt indicates double triplet. Chemical shifts are reported in ppm referenced to DMSO-*d*_*6*_ at 2.50 ppm for ^1^H NMR and at 39.5 ppm for ^13^C NMR, and coupling constants in hertz (Hz).

The assignment of the signals in solution is based on conventional 2D techniques, ^1^H–^1^H COSY, HMBC, and HSQC, and by comparisons with calculated values.

### Computational Details

All of the calculations were carried out using the Gaussian-16 package.^73^ In all cases, we used the B3LYP/6**-**311++G(d,p) method;^74,75^ frequency calculations were carried out to verify that the structures obtained correspond to energetic minima (*I* = 0) or to transition states (TS, *I* = 1). These geometries were used for the calculation of the absolute chemical shieldings with the GIAO method^76^ and the SSCC.

Equations 3–5 were used to transform absolute shieldings into chemical shifts^46^
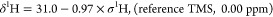
3
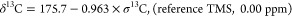
4
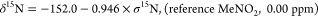
5To locate the intermediates at either sites of the TS point, we followed the vibrational mode of the imaginary frequency, forward and backward, along the intrinsic reaction coordinate (IRC)^77,78^ and relaxed the geometry for searching an energy (local) minimum. Although all of the stationary points were calculated at the B3LYP/6**-**311++G(d,p) level, they were recalculated at the 6-31G* level^79^ to calculate the IRCs.

To have a better description of the energy, domain-based local pair natural orbital coupled cluster method with single, double, and perturbative triple excitations, DLPNO-CCSD(T),^80,81^ with the def2-TZVP basis set^82^ has been carried out on the B3LYP/6**-**311++G(d,p) geometries with the Orca program (Version 5.0.1).^83^ The effect of the solvent has been taken into account by optimizing the structures using the polarizable continuum model (PCM)^84^ with the water parameters at the B3LYP/6**-**311++G(d,p) level.

## Supporting Information Available

Reproduction of the ^1^H and ^13^C NMR spectra of all novel compounds; details of computational methods and details of the crystal structure determinations of **2a** (2113708), **2b** (2113709), **2c** (2113710), and **2d** (2113711) (PDF)

## Accession Codes

CCDC 2113708–2113711 contains the supplementary crystallographic data for this paper. These data can be obtained free of charge via www.ccdc.cam.ac.uk/data_request/cif, or by emailing data_request@ccdc.cam.ac.uk, or by contacting The Cambridge Crystallographic Data Centre, 12 Union Road, Cambridge CB2 1EZ, UK; fax: +44 1223 336033.
