# Supplementary material for: Study of the Addition Mechanism of 1H-Indazole and Its 4-, 5-, 6-, and 7-Nitro Derivatives to Formaldehyde in Aqueous Hydrochloric Acid Solutions
Source: J Org Chem. 2022 Apr 11;87(9):5866–81. doi: 10.1021/acs.joc.2c00154 (PMC9087356; doi:10.1021/acs.joc.2c00154)
Supplement: Supplementary file 1 — jo2c00154_si_001.pdf [file jo2c00154_si_001.pdf]

Supporting Information for

Study of the addition mechanism of 1*H*-indazole and its 4-, 5-, 6- and 7-nitro derivatives to formaldehyde in aqueous hydrochloric acid solution

Ibon Alkorta,[a,*] Rosa M. Claramunt,[b] José Elguero,[a] Enrique Gutiérrez-Puebla,[c] M. Ángeles Monge,[c] Felipe Reviriego,[d] and Christian Roussel[e]

[a] Instituto de Química Médica, CSIC, Juan de la Cierva, 3, E-28006 Madrid, Spain
[b] Departamento de Química Orgánica y Bio-Orgánica, Facultad de Ciencias, UNED, Senda del Rey 9, E-28040 Madrid, Spain
[c] Departamento de Nuevas Arquitecturas en Química de Materiales, Instituto de Ciencia de Materiales de Madrid (ICMM-CSIC), Sor Juana Inés de la Cruz, 3, Cantoblanco, E-28049 Madrid, Spain
[d] Instituto de Ciencia y Tecnología de Polímeros, CSIC, Juan de la Cierva, 3, E-28006 Madrid, Spain
[e] Aix-Marseille Université, CNRS, Centrale Marseille, iSm2, 13397 Marseille, France

*Correspondence to: ibon@iqm.csic.es

Table of contents:

Pg. S2-3 ChemDraw of all the studied molecules.

Pg. S4-19 NMR and ATR spectra of compounds reported in the manuscript **2a**, **2b**, **2c**, **2d**, **3b**, **1e**-2*H*, **3e**, Figures **S1** to **S29**.

Pg. S20-24 Thermal ellipsoid plot for each crystal structure of full-page size, Figures **S30** to **S33**, and Table S1.

Pg. S25-71 Computational data including electronic energy and optimized geometry at B3LYP/6-311++G(d,p) computational level, GIAO, SSCC and IRC profile calculations.

Pg. S72 Table S2. Energies (kJ·mol^{-1}) corresponding to Scheme 6; x = **a**, **b**, **c**, **d**, **e** in gas phase.

---

1. ChemDraw of all the studied molecules

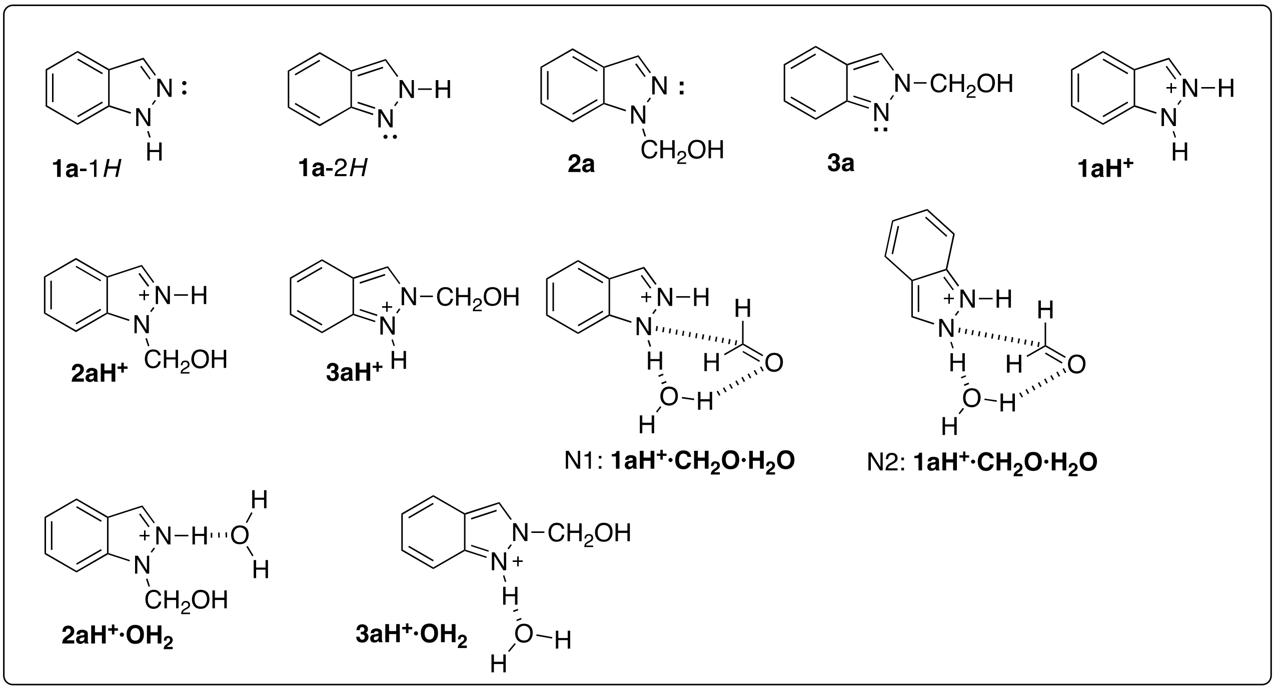

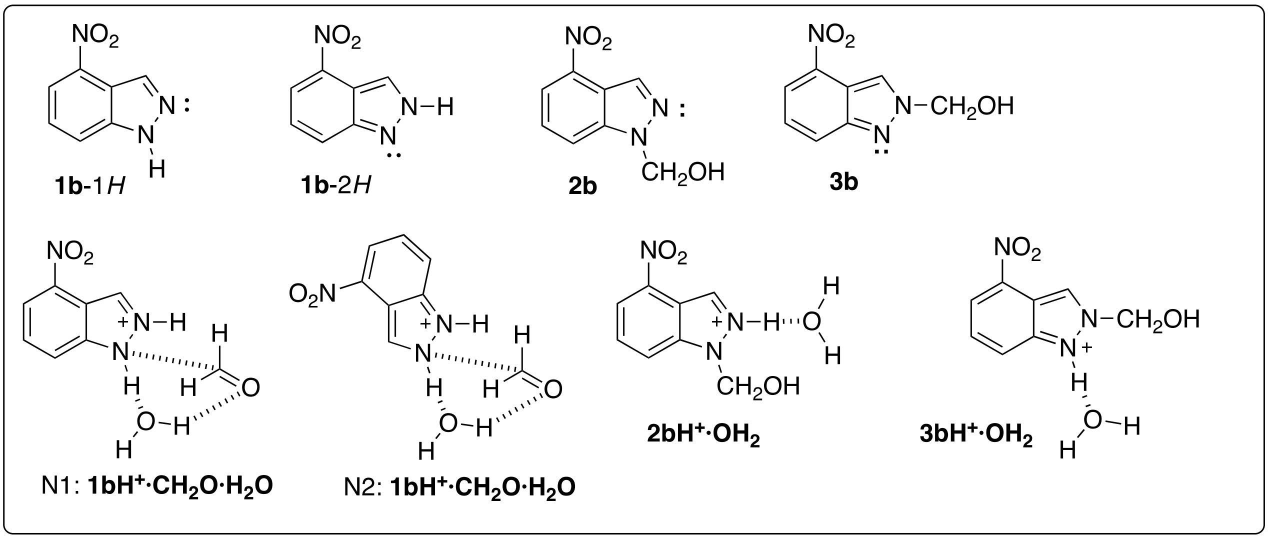

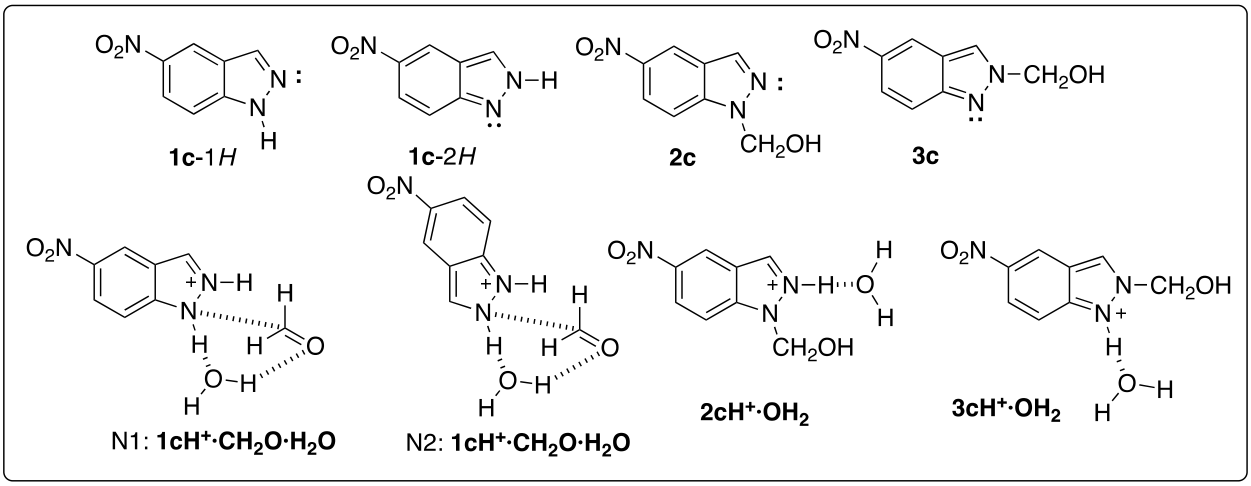
1c-1H
1c-2H
2c
3c
N1: 1cH+·CH2O·H2O
N2: 1cH+·CH2O·H2O
2cH+·OH2
3cH+·OH2

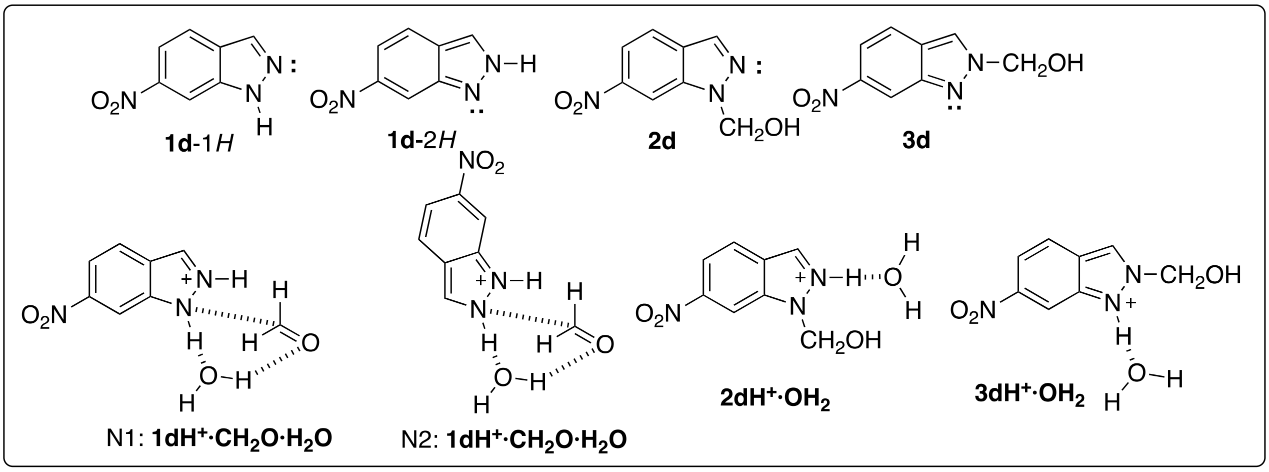
1d-1H
1d-2H
2d
3d
N1: 1dH+·CH2O·H2O
N2: 1dH+·CH2O·H2O
2dH+·OH2
3dH+·OH2

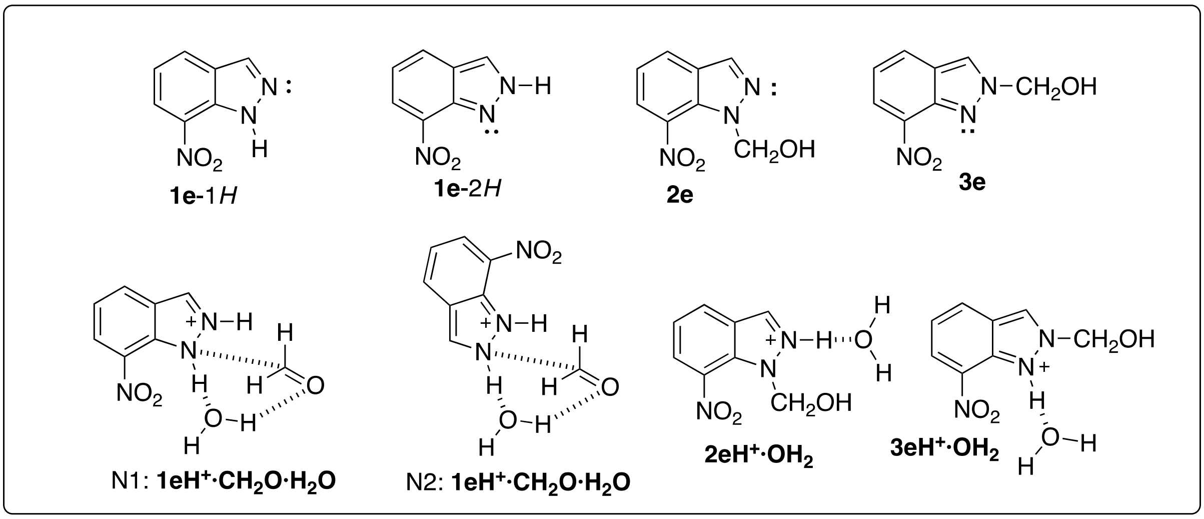
1e-1H
1e-2H
2e
3e
N1: 1eH+·CH2O·H2O
N2: 1eH+·CH2O·H2O
2eH+·OH2
3eH+·OH2

2. NMR and ATR spectra of compounds reported in the manuscript **2a**, **2b**, **2c**, **2d**, **3b**, **1e**-2*H*, **3e**.

Figure S1. ^{1}H NMR spectrum (400 MHz, DMSO-d_6) of 1-hydroxymethylindazole (**2a**)

Figure S2. ^{13}C{^{1}H} NMR spectrum (100 MHz, DMSO-d_6) of 1-hydroxymethylindazole (**2a**)

Figure S3. ATR spectrum of 1-hydroxymethylindazole (**2a**)

Figure S4. ^{1}H NMR spectrum (400 MHz, DMSO-d_6) of 1-hydroxymethyl-4-nitroindazole (**2b**) and 2-hydroxymethyl-4-nitroindazole (**3b**)

Figure S5. ^{1}H NMR spectrum (400 MHz, DMSO-d_6) of 1-hydroxymethyl-4-nitroindazole (**2b**)

Figure S6. ^{13}C{^{1}H} NMR spectrum (100 MHz, DMSO-d_6) of 1-hydroxymethyl-4-nitroindazole (**2b**)

Figure S7. ATR spectrum of 1-hydroxymethyl-4-nitroindazole (**2b**)

Figure S8. ^{1}H NMR spectrum (400 MHz, DMSO-d_6) of 1-hydroxymethyl-5-nitroindazole (**2c**)

Figure S9. ^{13}C{^{1}H} NMR spectrum (100 MHz, DMSO-d_6) of 1-hydroxymethyl-5-nitroindazole (**2c**)

Figure S10. ATR spectrum of 1-hydroxymethyl-5-nitroindazole (**2c**)

Figure S11. ^{1}H NMR spectrum (400 MHz, DMSO-d_6) of 1-hydroxymethyl-6-nitroindazole (**2d**)

Figure S12. ^{13}C{^{1}H} NMR spectrum (100 MHz, DMSO-d_6) of 1-hydroxymethyl-6-nitroindazole (**2d**)

Figure S13. ATR spectrum of 1-hydroxymethyl-6-nitroindazole (**2d**)

Figure S14. ^{1}H NMR spectrum (500 MHz, DMSO-d_6) of 7-nitro-1*H*-indazole (**1e**)

Figure S15. ^{13}C{^{1}H} NMR spectrum (125 MHz, DMSO-d_6) of 7-nitro-1*H*-indazole (**1e**)

Figure S16. ^{1}H NMR spectrum (500 MHz, DMSO-d_6) of 2-hydroxymethyl-7-nitroindazole (**3e**) and 7-nitro-1*H*-indazole (**1e**)

Figure S17. ^{1}H NMR spectrum (500 MHz, DMSO-d_6) of 1-hydroxymethyl-7-nitroindazole (**2e**), 2-hydroxymethyl-7-nitroindazole (**3e**) and 7-nitro-1*H*-indazole (**1e**)

Figure S18. ^{13}C{^{1}H} NMR spectrum (125 MHz, DMSO-d_6) of 1-hydroxymethyl-7-nitroindazole (**2e**), 2-hydroxymethyl-7-nitroindazole (**3e**) and 7-nitro-1*H*-indazole (**1e**)

Figure S19. ^{13}C NMR spectrum (100 MHz, CPMAS) of 1-hydroxymethylindazole (**2a**)

Figure S20. ^{13}C NMR spectrum (100 MHz, CPMAS) of 1-hydroxymethyl-4-nitroindazole (**2b**)

Figure S21. ^{13}C NMR spectrum (100 MHz, CPMAS) of 1-hydroxymethyl-5-nitroindazole (**2c**)

Figure S22. ^{13}C NMR spectrum (100 MHz, CPMAS) of 1-hydroxymethyl-6-nitroindazole (**2d**)

Figure S23. ^{15}N NMR spectrum (40.5 MHz, CPMAS) of 1-hydroxymethylindazole (**2a**)

Figure S24. ^{15}N NMR spectrum (40.5 MHz, CPMAS) of 1-hydroxymethyl-4-nitroindazole (**2b**)

Figure S25. ^{15}N NMR spectrum (40.5 MHz, CPMAS) of 1-hydroxymethyl-5-nitroindazole (**2c**)

Figure S26. ^{15}N NMR spectrum (40.5 MHz, CPMAS) of 1-hydroxymethyl-6-nitroindazole (**2d**)

Figure S27. 2D(^{15}N-1H) HMBC NMR spectrum (40.5 MHz, DMSO-d_6) of 1-hydroxymethyl-4-nitroindazole (**2b**)

Figure S28. ^{15}N NMR spectrum (40.5 MHz, DMSO-d_6) of 1-hydroxymethyl-5-nitroindazole (**2c**)

Figure S29. ^{15}N NMR spectrum (40.5 MHz, DMSO-d_6) of 1-hydroxymethyl-6-nitroindazole (**2d**)

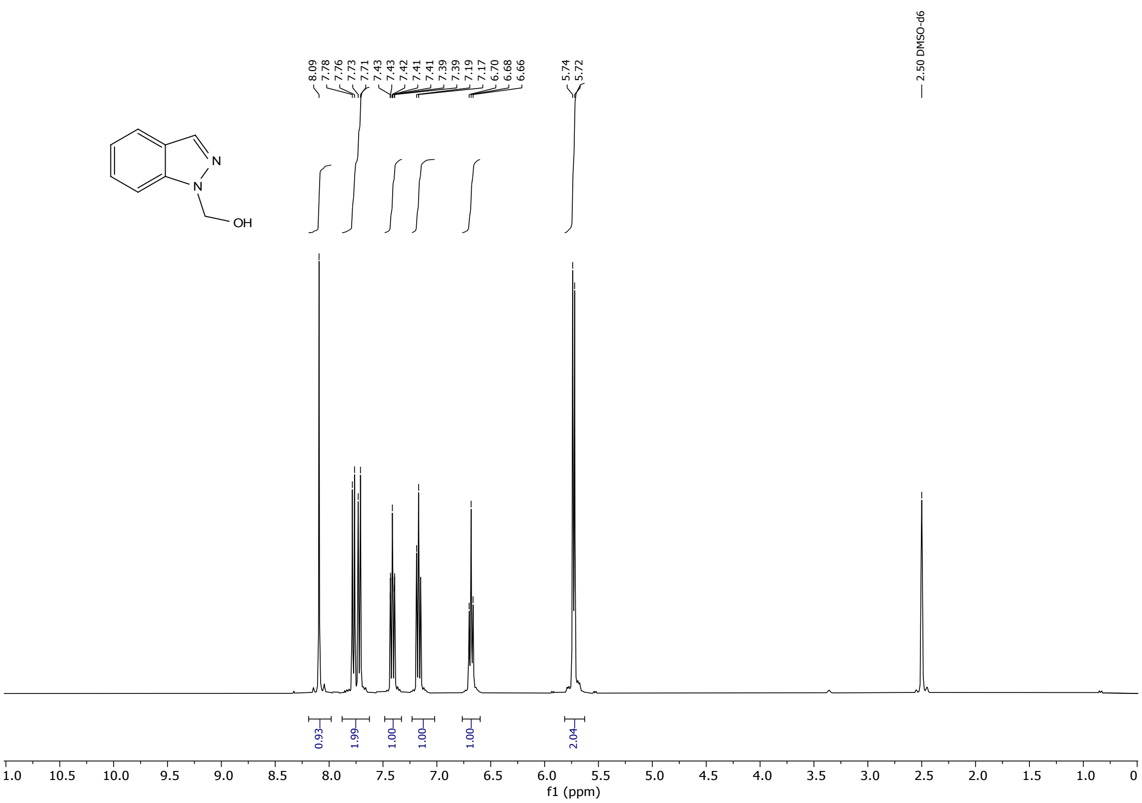

Figure S1. ^{1}H NMR spectrum (400 MHz, DMSO-d_6) of 1-hydroxymethylindazole (**2a**)

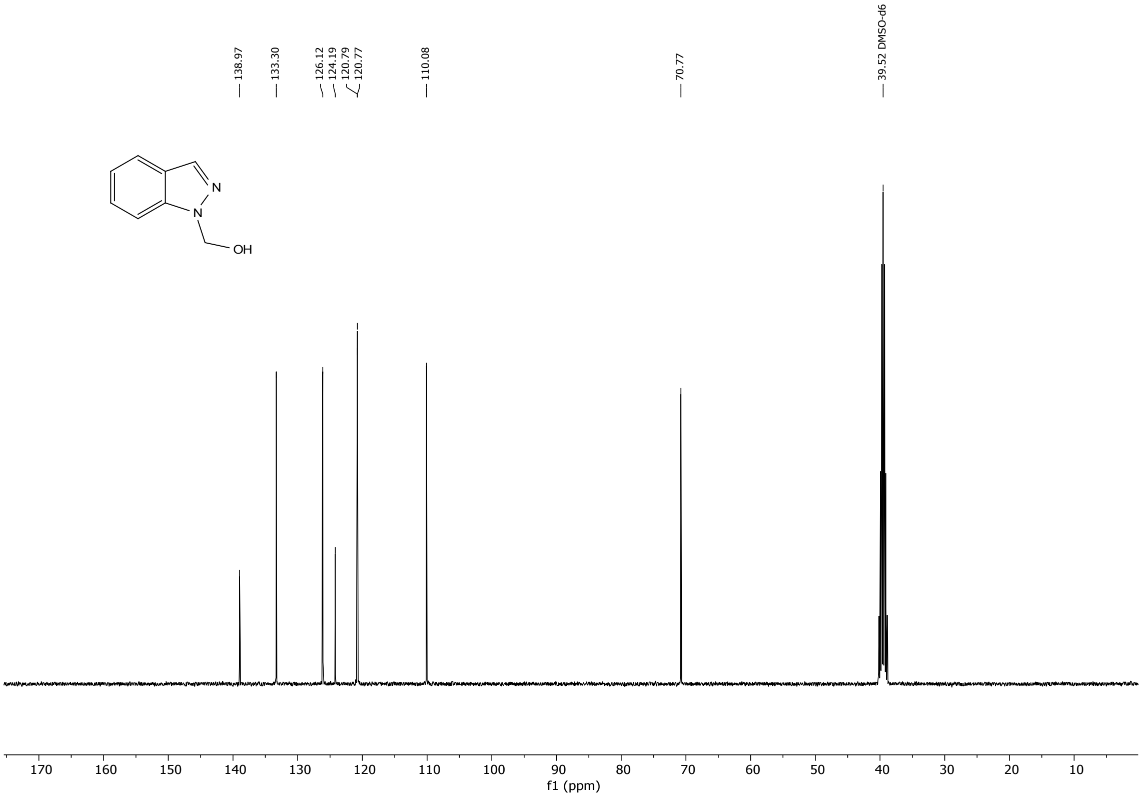

Figure S2. ^{13}C{^{1}H} NMR spectrum (100 MHz, DMSO-d_6) of 1-hydroxymethylindazole (**2a**)

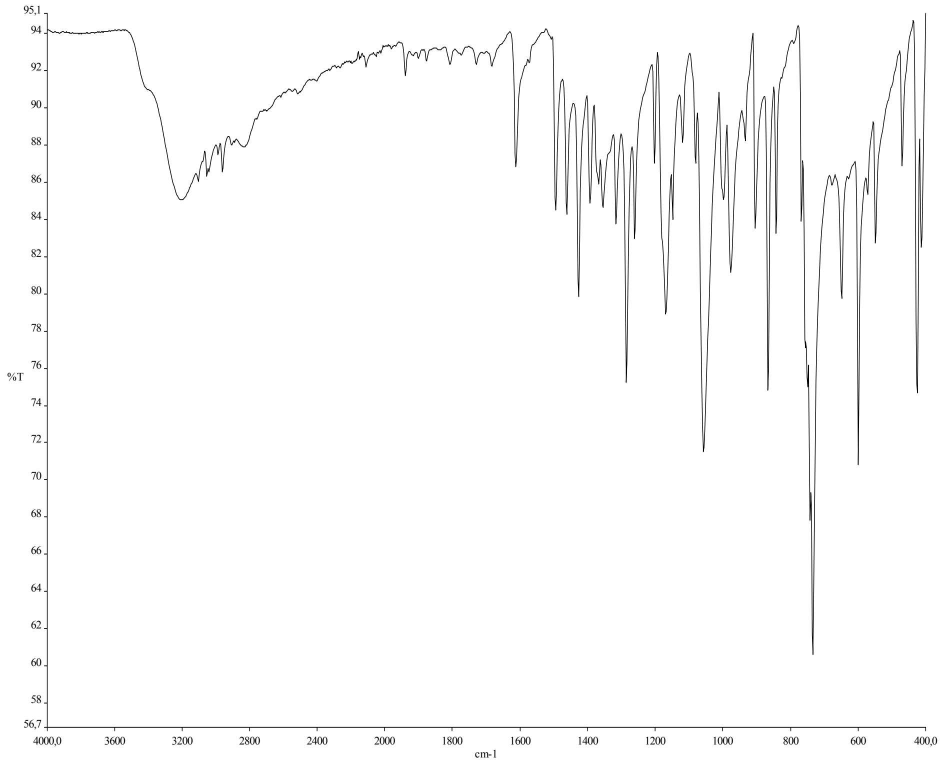

Figure S3. ATR spectrum of 1-hydroxymethylindazole (**2a**)

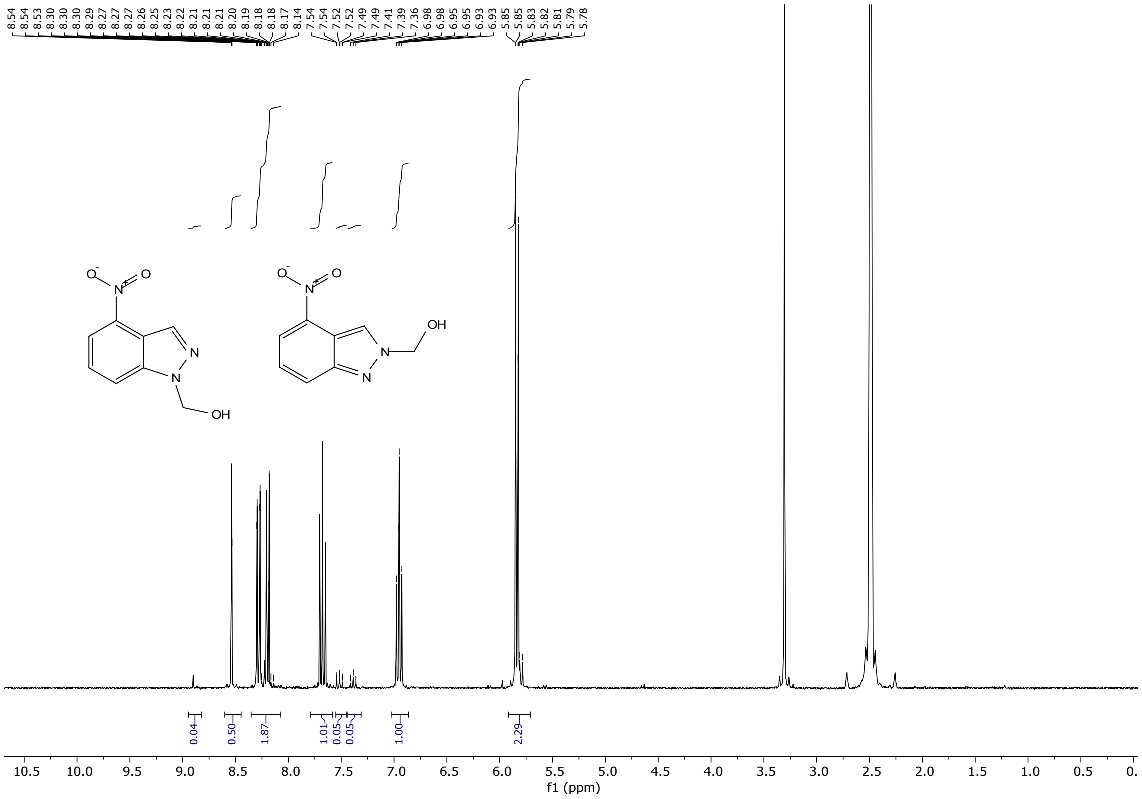

Figure S4. ^{1}H NMR spectrum (400 MHz, DMSO-d_6) of 1-hydroxymethyl-4-nitroindazole (**2b**) and 2-hydroxymethyl-4-nitroindazole (**3b**)

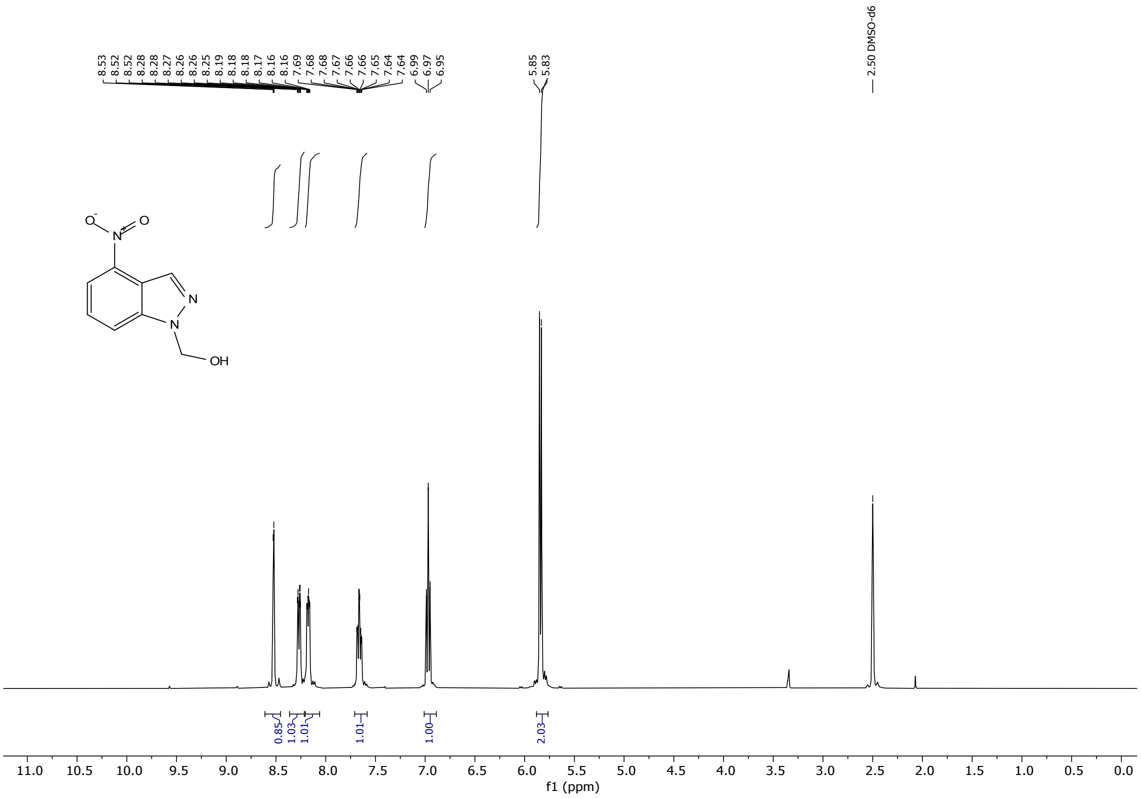

Figure S5. ^{1}H NMR spectrum (400 MHz, DMSO-d_6) of 1-hydroxymethyl-4-nitroindazole (**2b**)

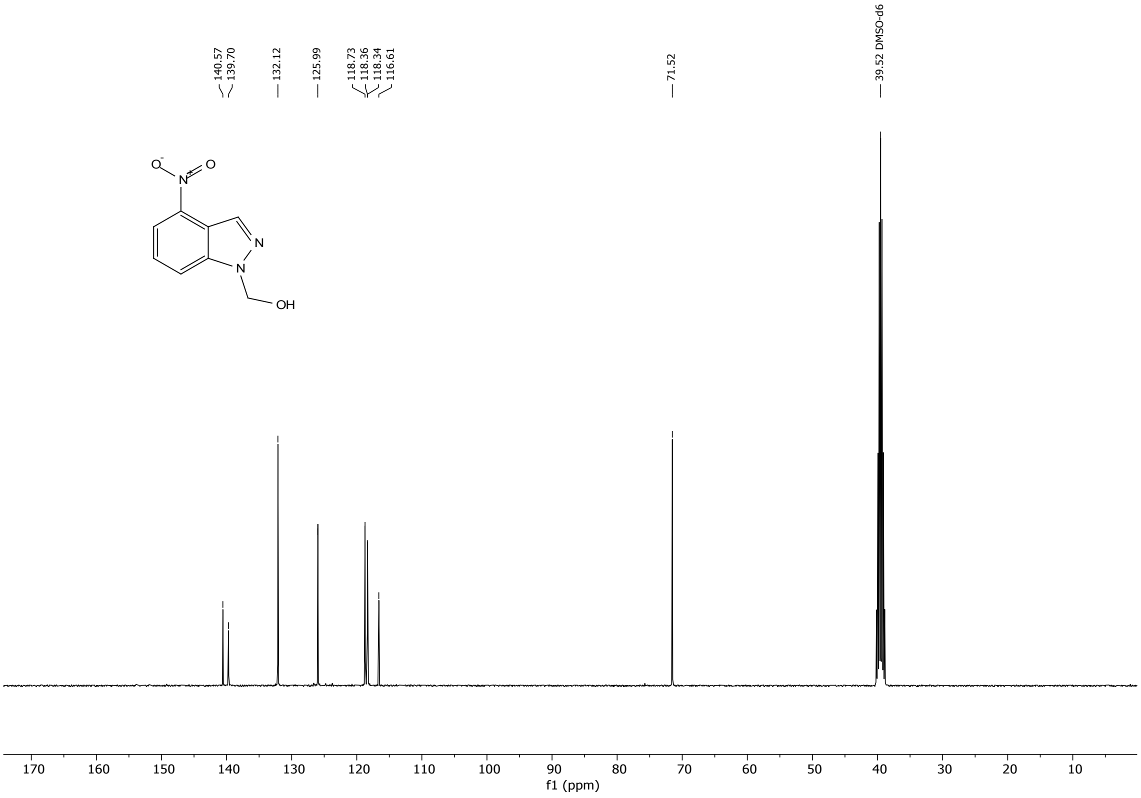

Figure S6. $^{13}C\{^{1}H\}$ NMR spectrum (100 MHz, DMSO-d_6) of 1-hydroxymethyl-4-nitroindazole (**2b**)

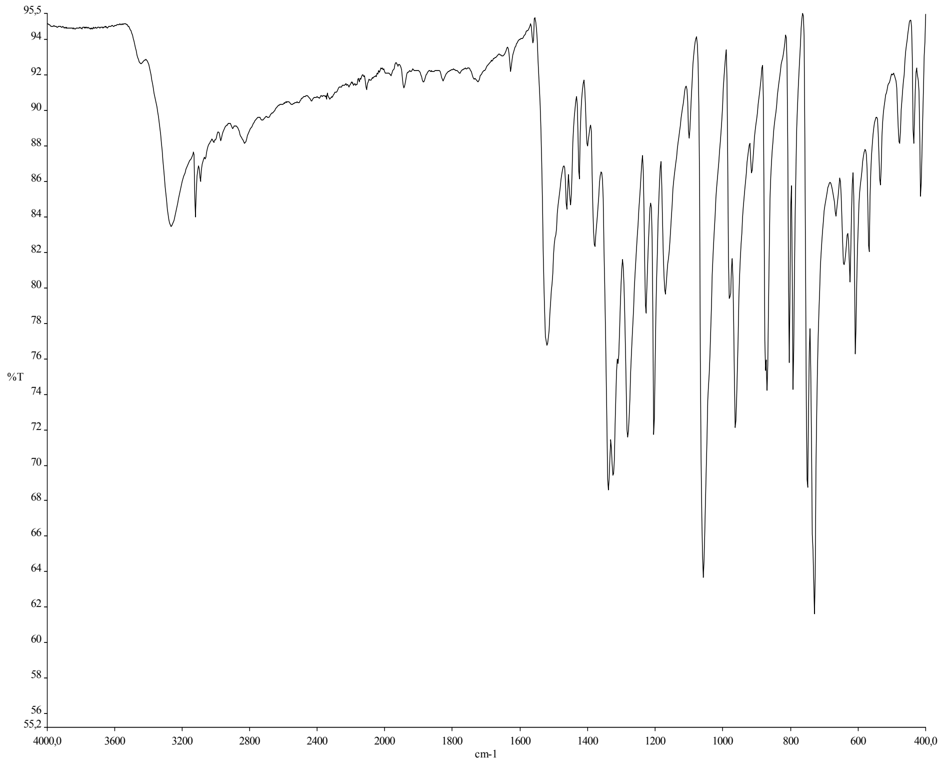

Figure S7. ATR spectrum of 1-hydroxymethyl-4-nitroindazole (**2b**)

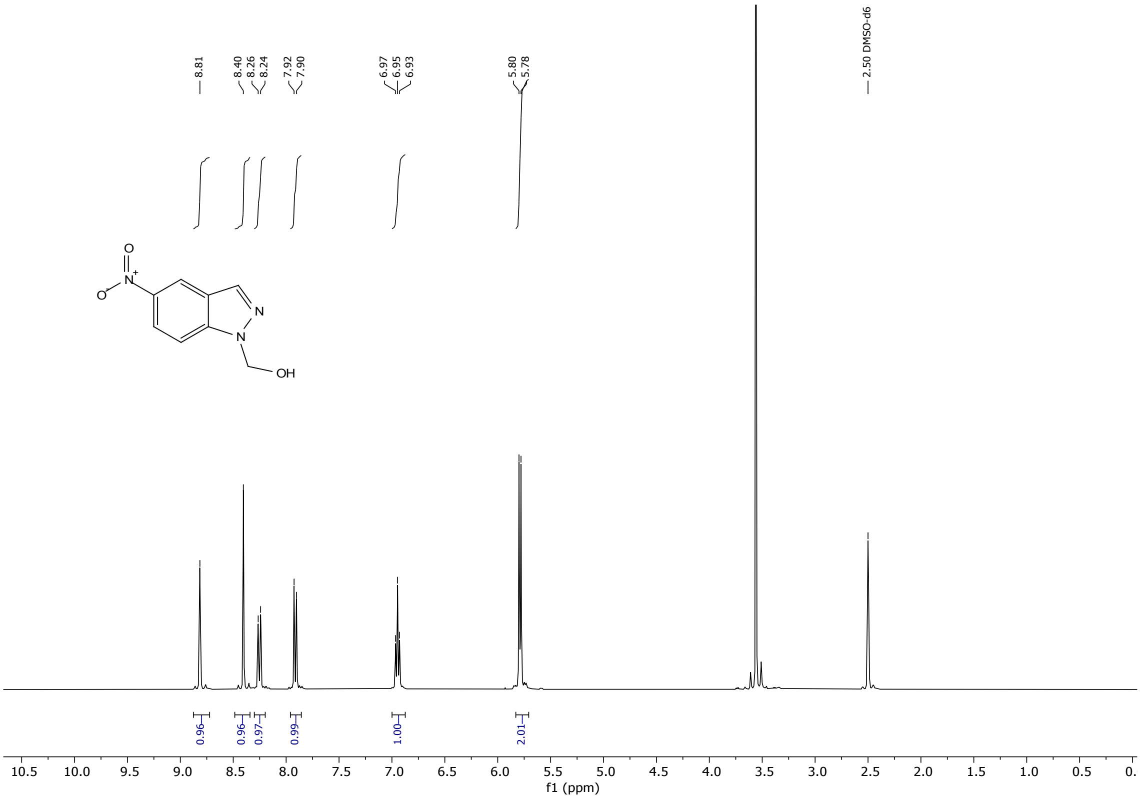

Figure S8. ^{1}H NMR spectrum (400 MHz, DMSO-d_6) of 1-hydroxymethyl-5-nitroindazole (**2c**)

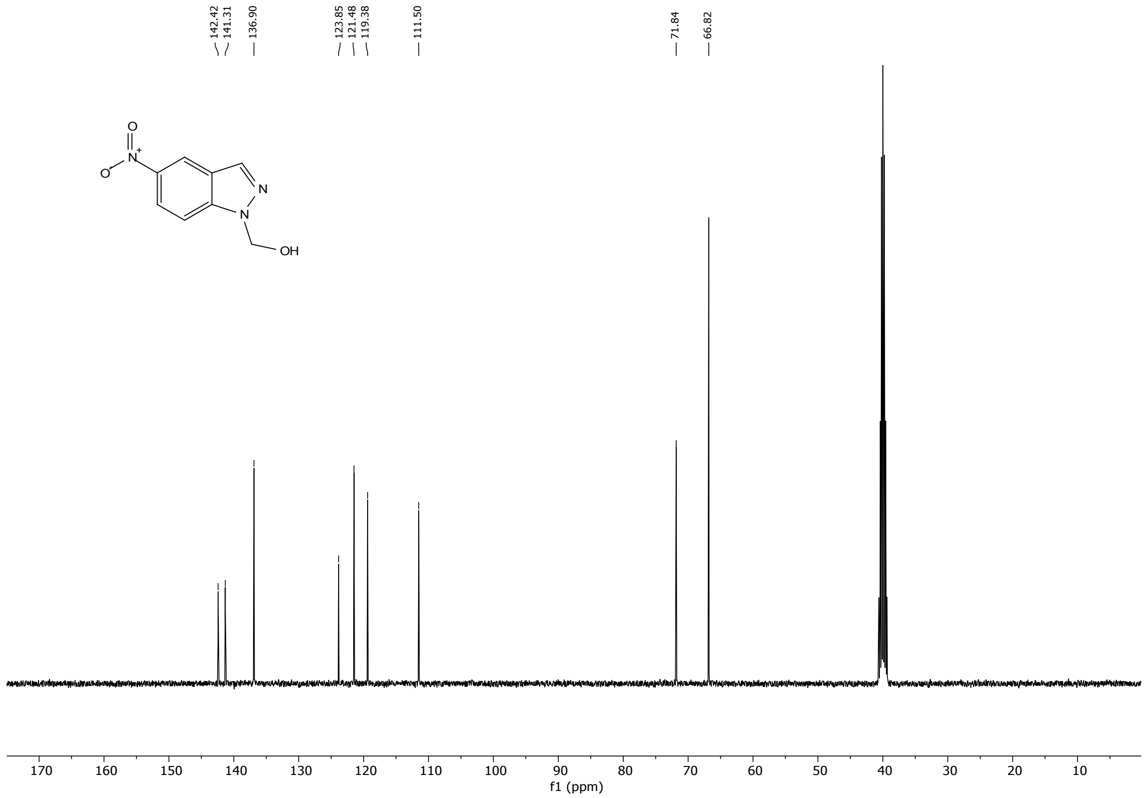

Figure S9. $^{13}C\{^{1}H\}$ NMR spectrum (100 MHz, DMSO-d_6) of 1-hydroxymethyl-5-nitroindazole (**2c**)

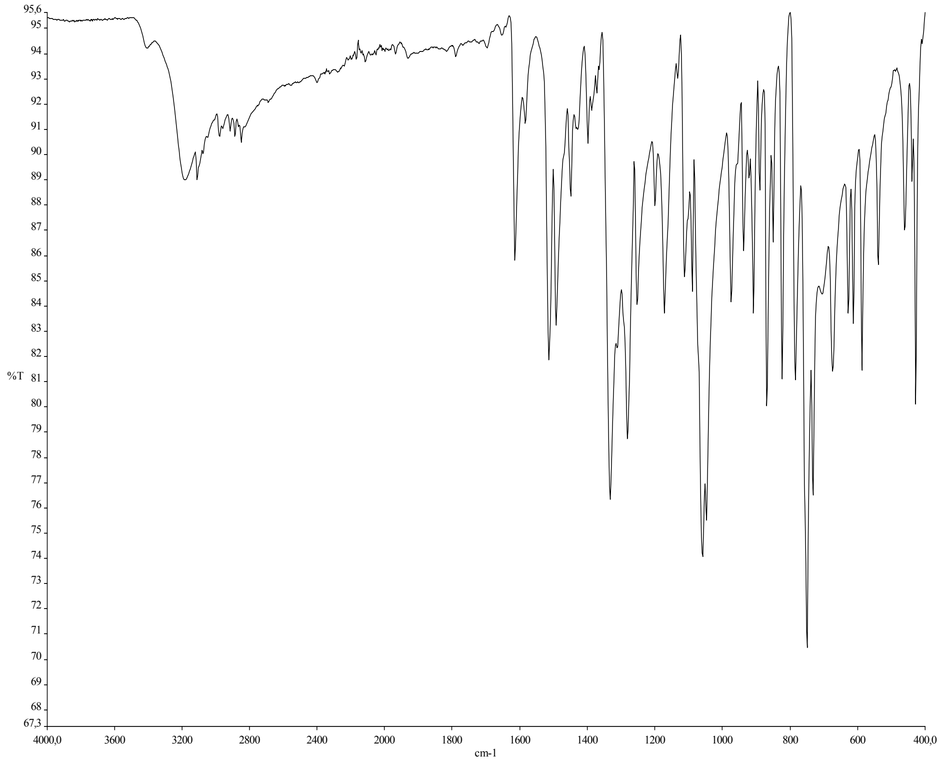

Figure S10. ATR spectrum of 1-hydroxymethyl-5-nitroindazole (**2c**)

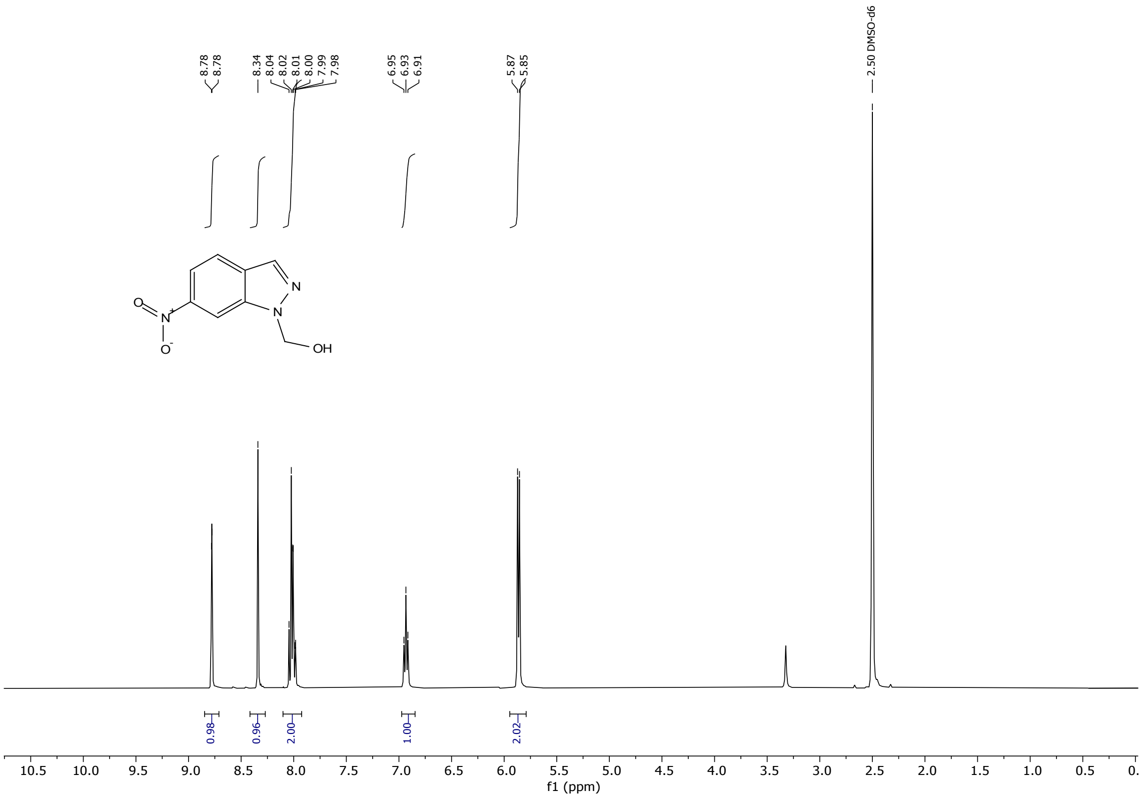

Figure S11. ^{1}H NMR spectrum (400 MHz, DMSO-d_6) of 1-hydroxymethyl-6-nitroindazole (**2d**)

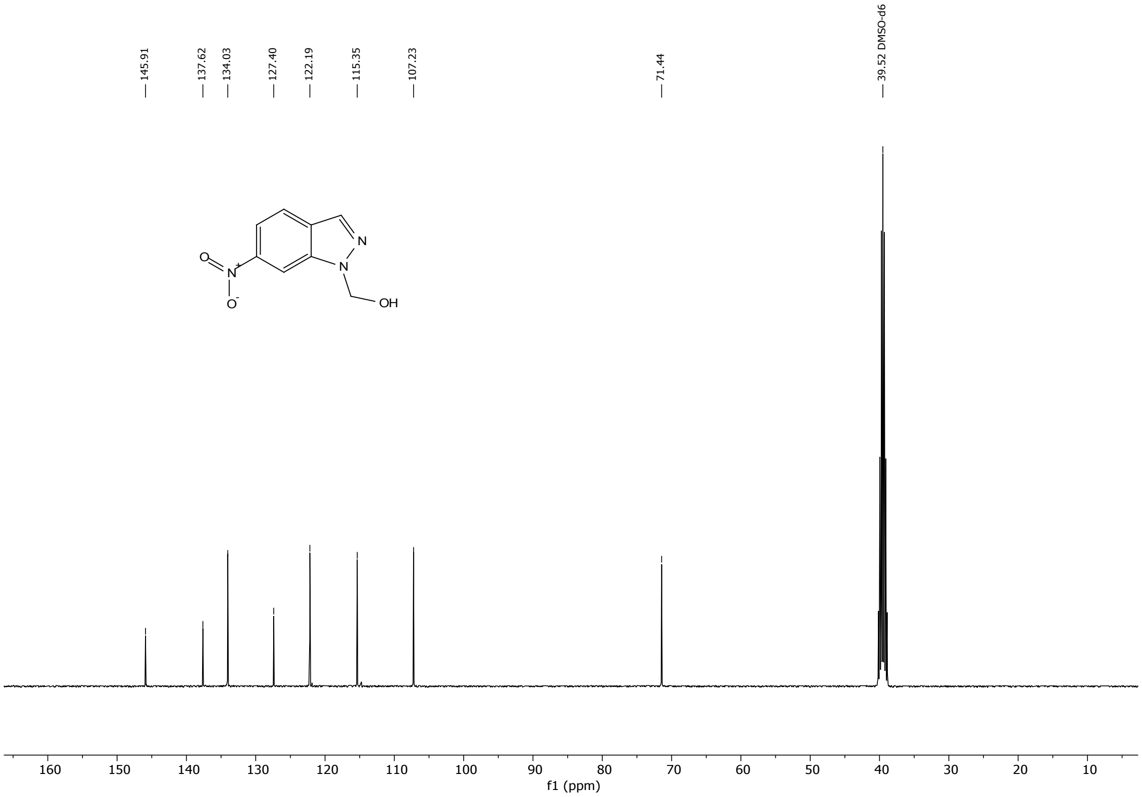

Figure S12. ^{13}C{^{1}H} NMR spectrum (100 MHz, DMSO-d_6) of 1-hydroxymethyl-6-nitroindazole (**2d**)

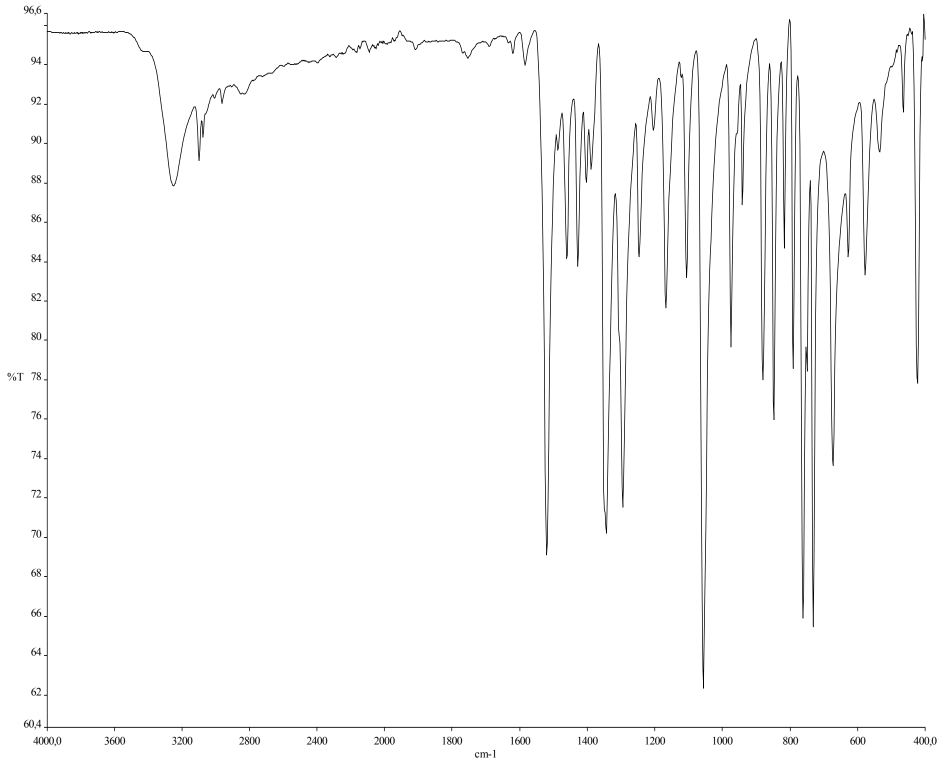

Figure S13. ATR spectrum of 1-hydroxymethyl-6-nitroindazole (**2d**)

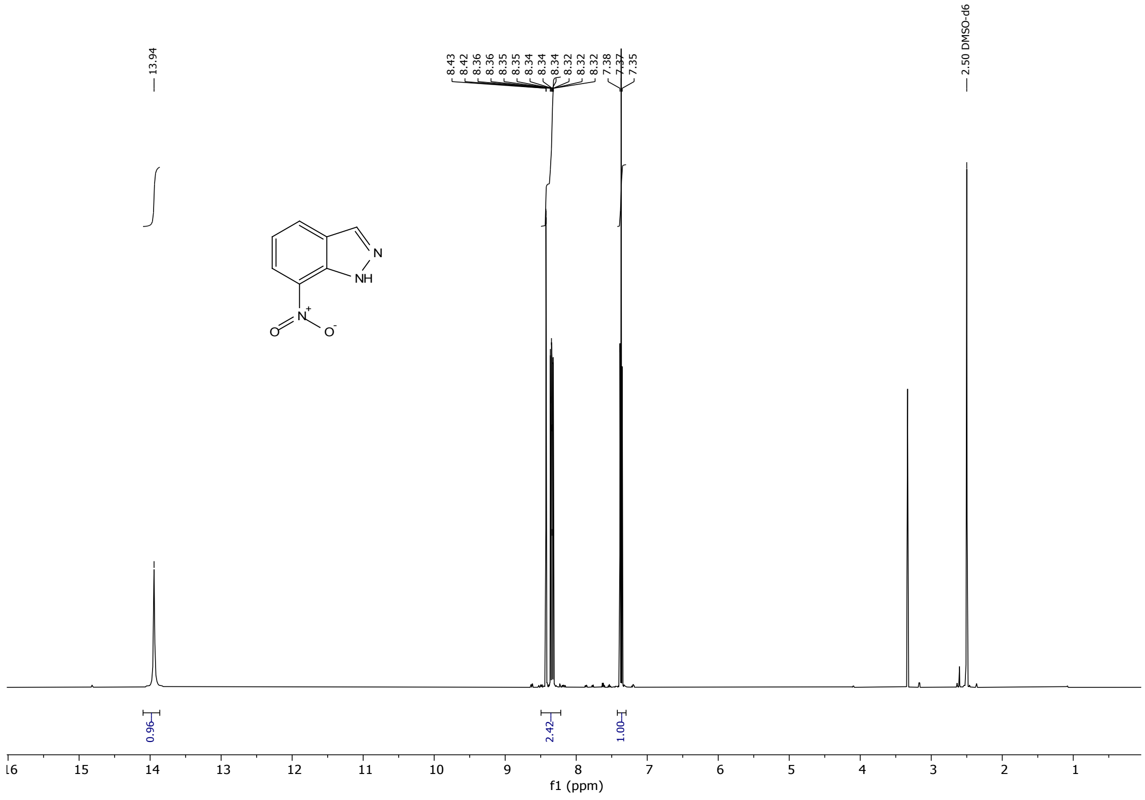

Figure S14. ^{1}H NMR spectrum (500 MHz, DMSO-d_6) of 7-nitro-1H-indazole (**1e**)

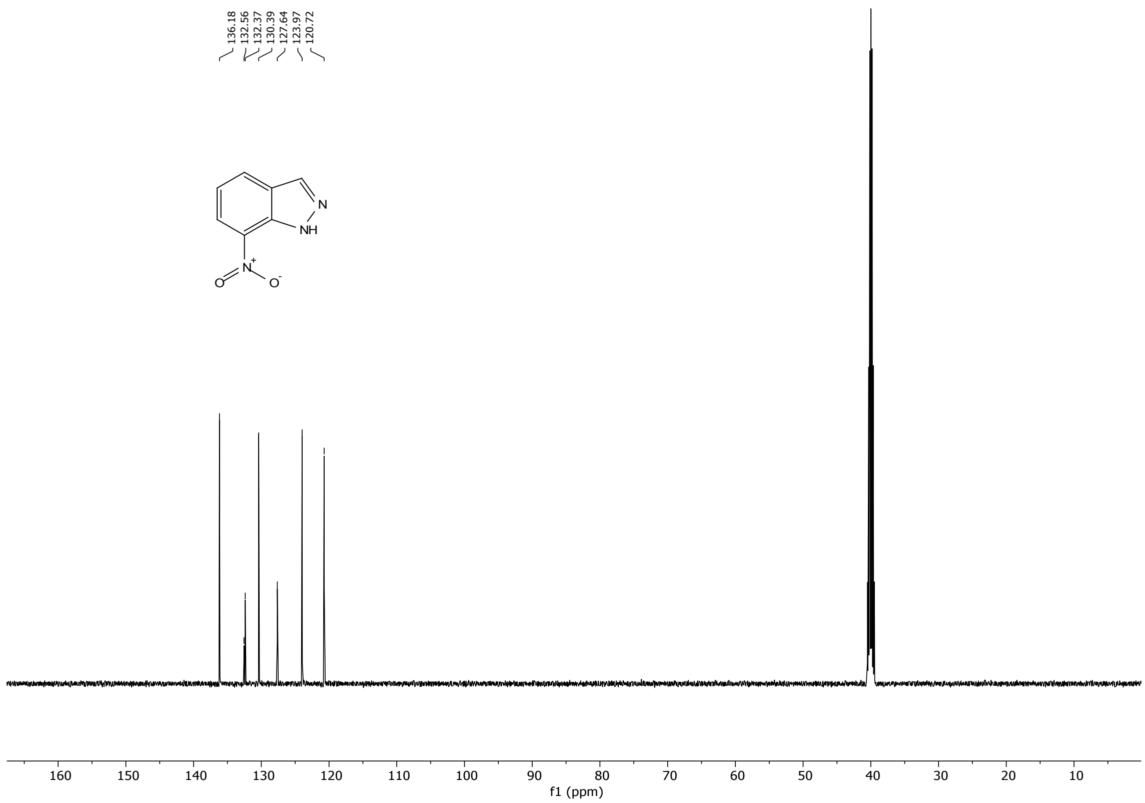

Figure S15. $^{13}C\{^1H\}$ NMR spectrum (125 MHz, DMSO-d_6) of 7-nitro-1*H*-indazole (**1e**)

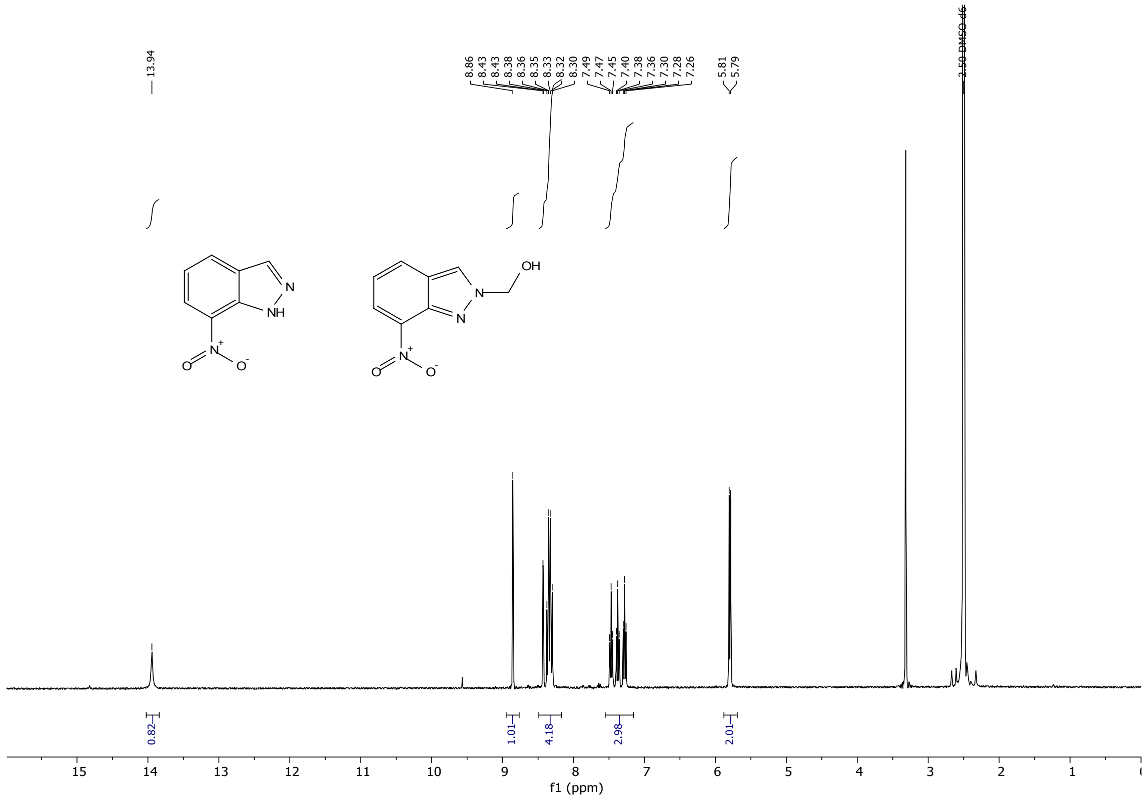

Figure S16. ^1H NMR spectrum (500 MHz, DMSO-d_6) of 2-hydroxymethyl-7-nitroindazole (**3e**) and 7-nitro-1*H*-indazole (**1e**)

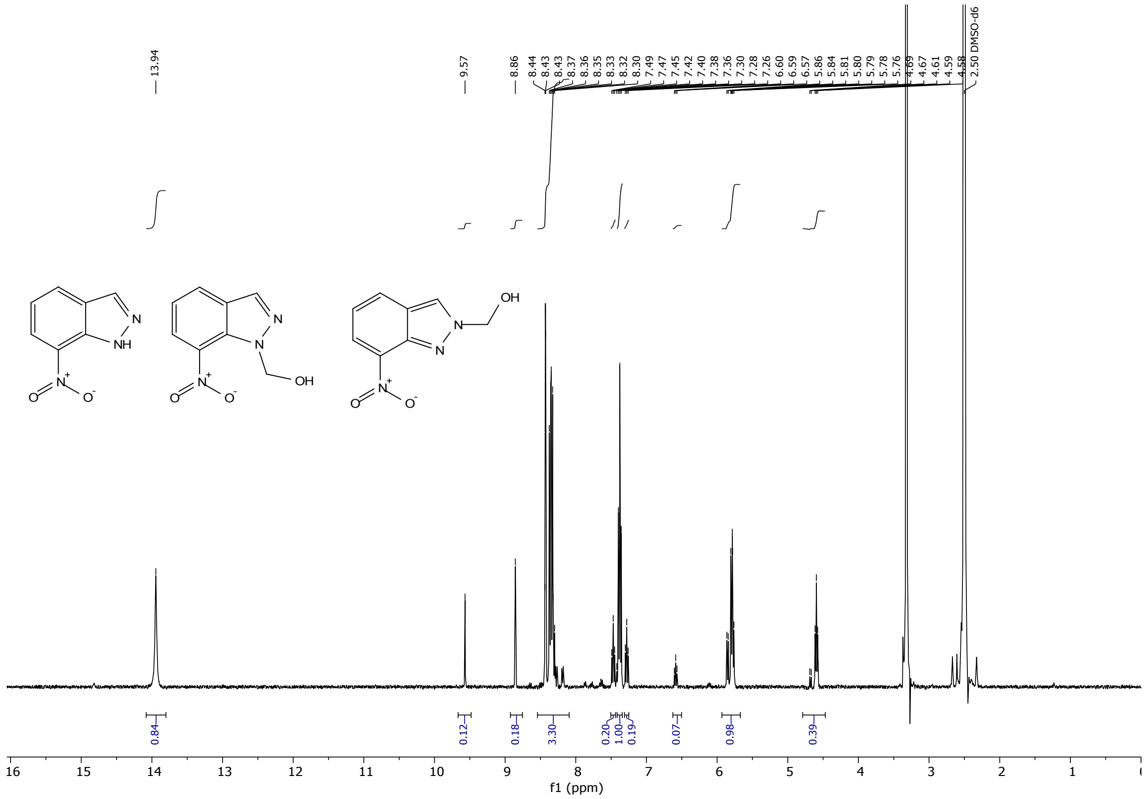

Figure S17. ^{1}H NMR spectrum (500 MHz, DMSO-d_6) of 1-hydroxymethyl-7-nitroindazole (**2e**), 2-hydroxymethyl-7-nitroindazole (**3e**) and 7-nitro-1*H*-indazole (**1e**)

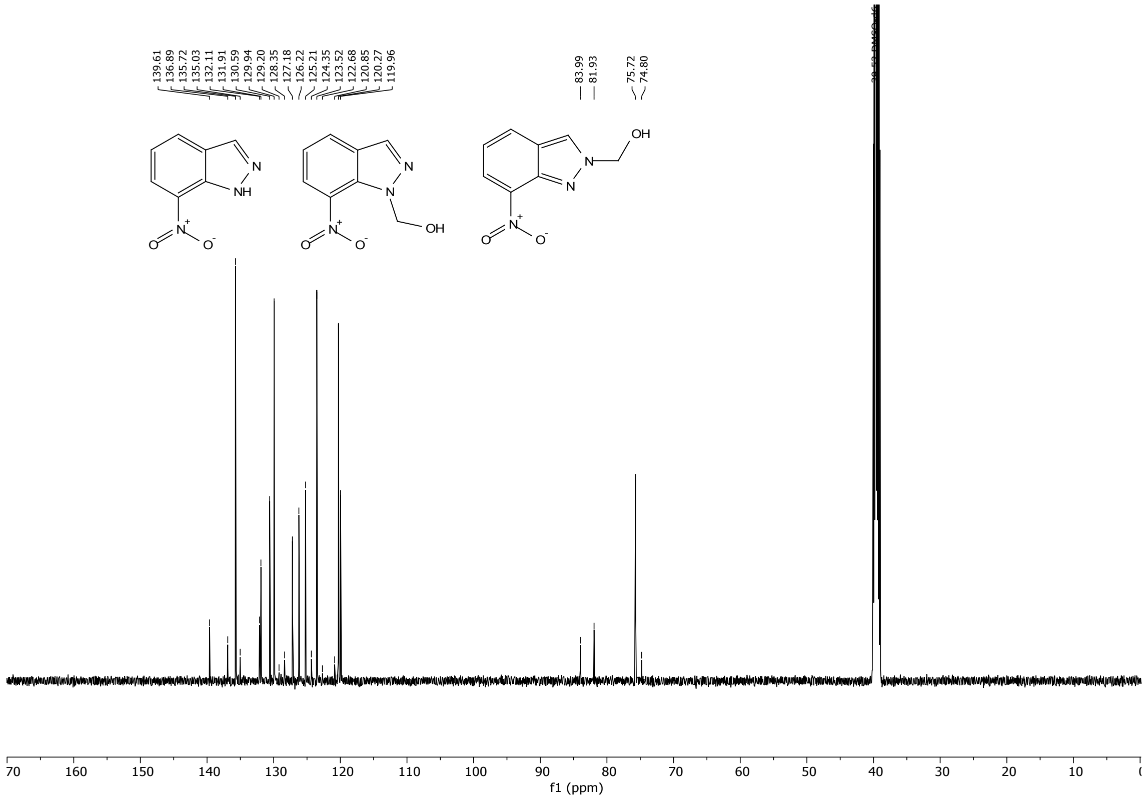

Figure S18. $^{13}C\{^{1}H\}$ NMR spectrum (125 MHz, DMSO-d_6) of 1-hydroxymethyl-7-nitroindazole (**2e**), 2-hydroxymethyl-7-nitroindazole (**3e**) and 7-nitro-1*H*-indazole (**1e**)

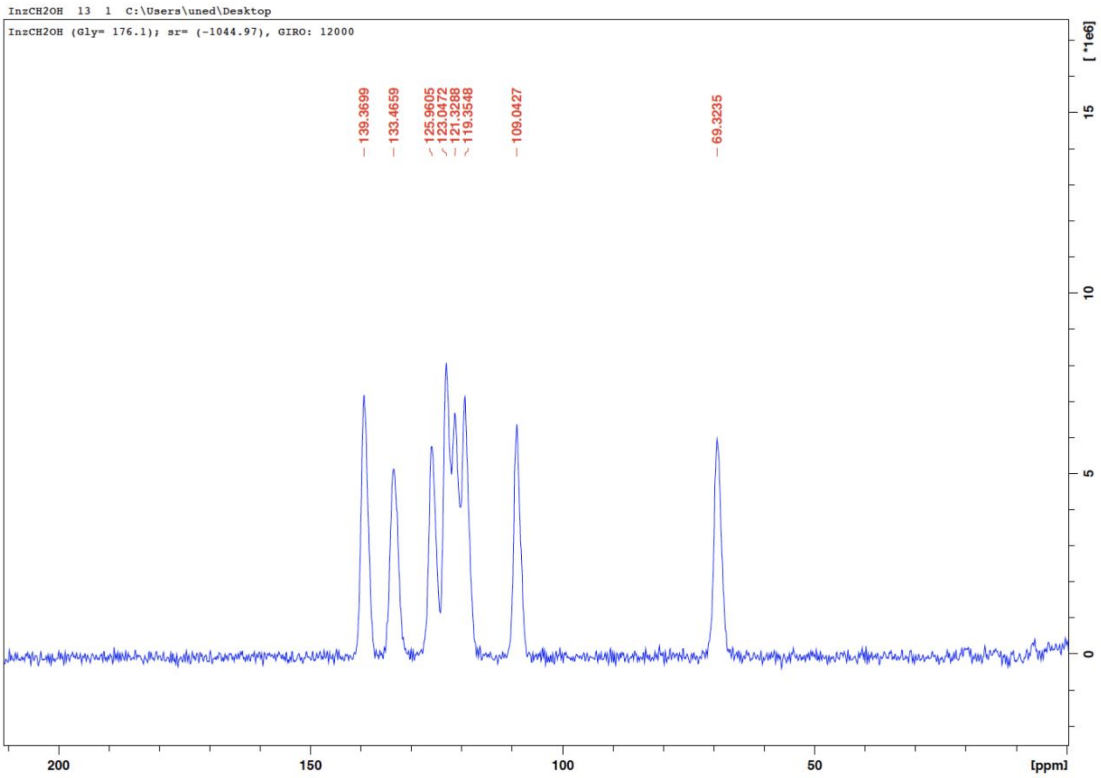

Figure S19. ^{13}C NMR spectrum (100 MHz, CPMAS) of 1-hydroxymethylindazole (**2a**)

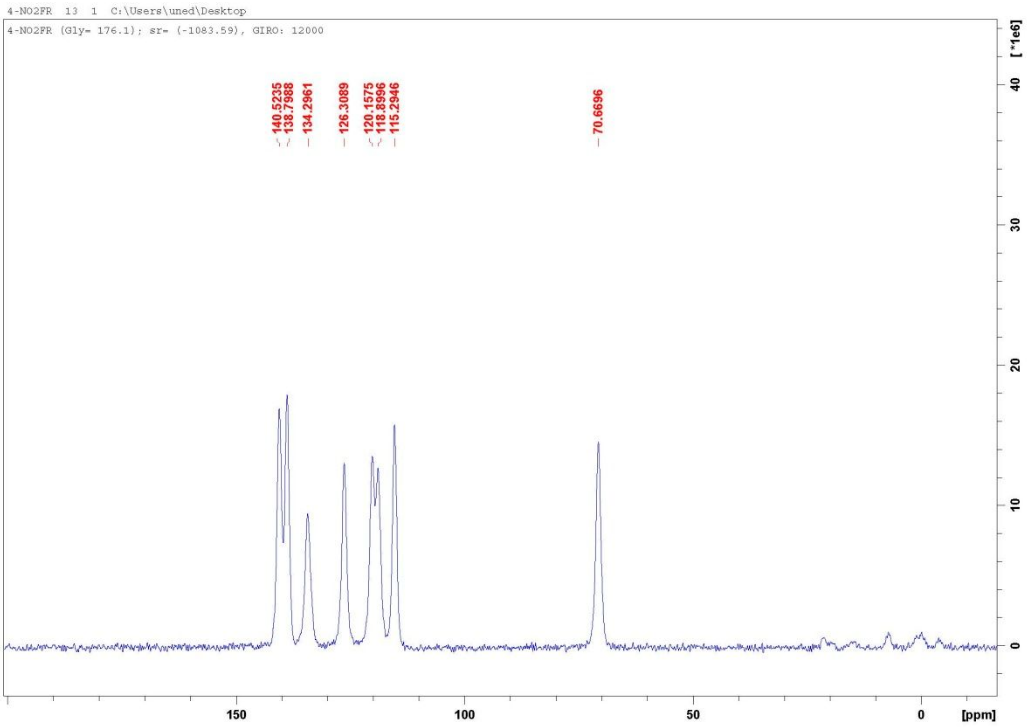

Figure S20. ^{13}C NMR spectrum (100 MHz, CPMAS) of 1-hydroxymethyl-4-nitroindazole (**2b**)

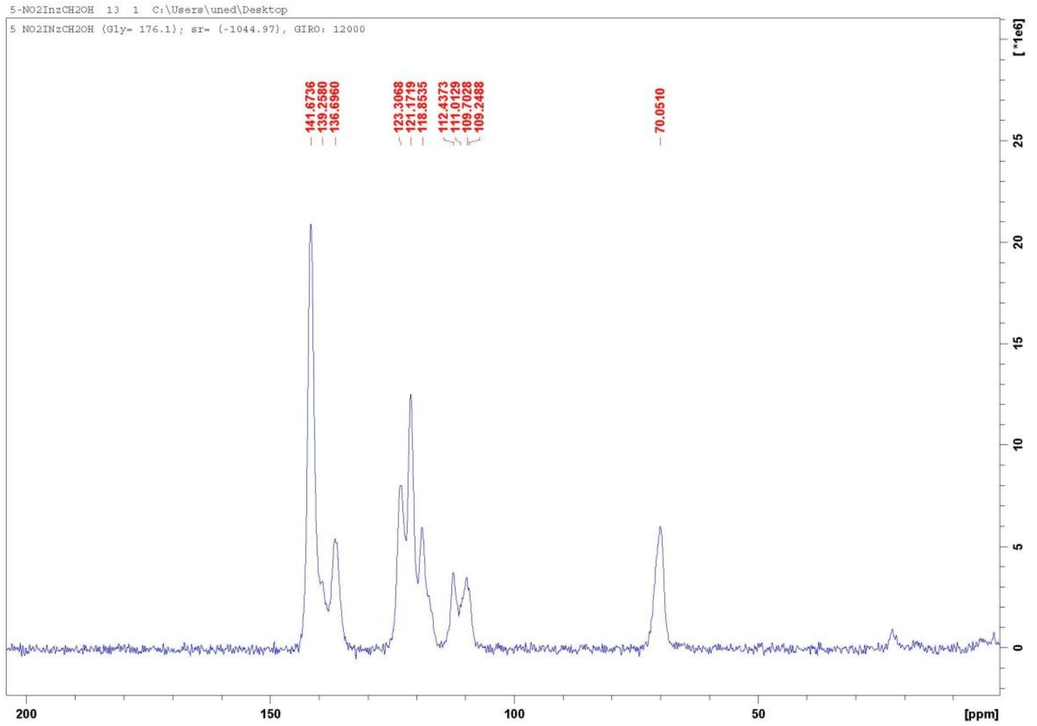

Figure S21. ^{13}C NMR spectrum (100 MHz, CPMAS) of 1-hydroxymethyl-5-nitroindazole (**2c**)

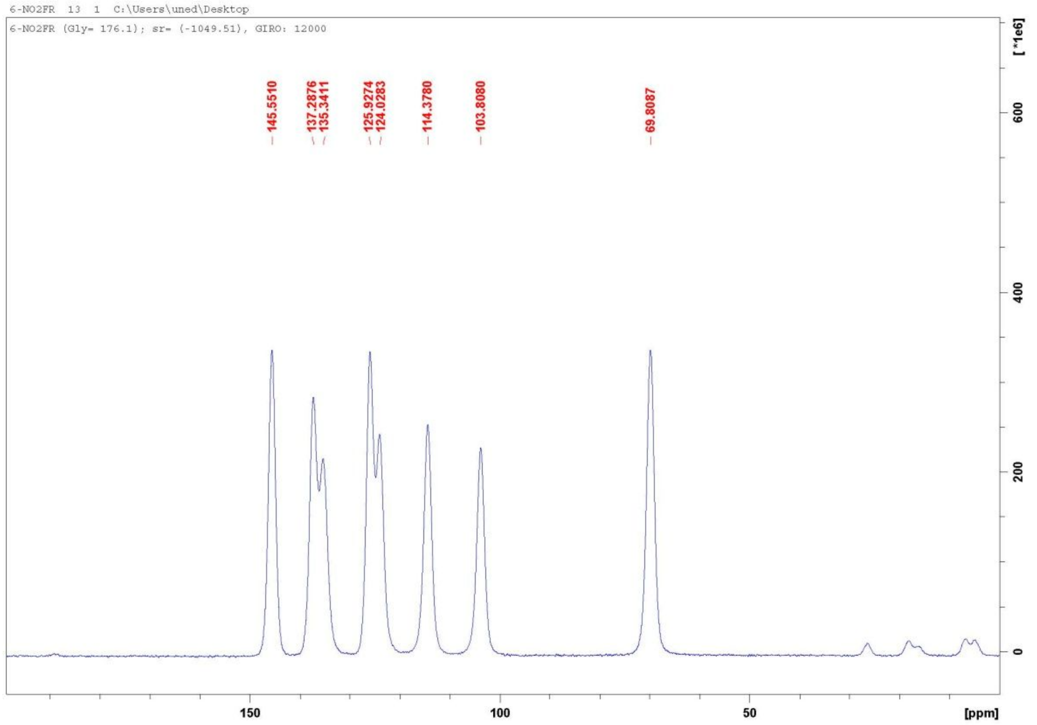

Figure S22. ^{13}C NMR spectrum (100 MHz, CPMAS) of 1-hydroxymethyl-6-nitroindazole (**2d**)

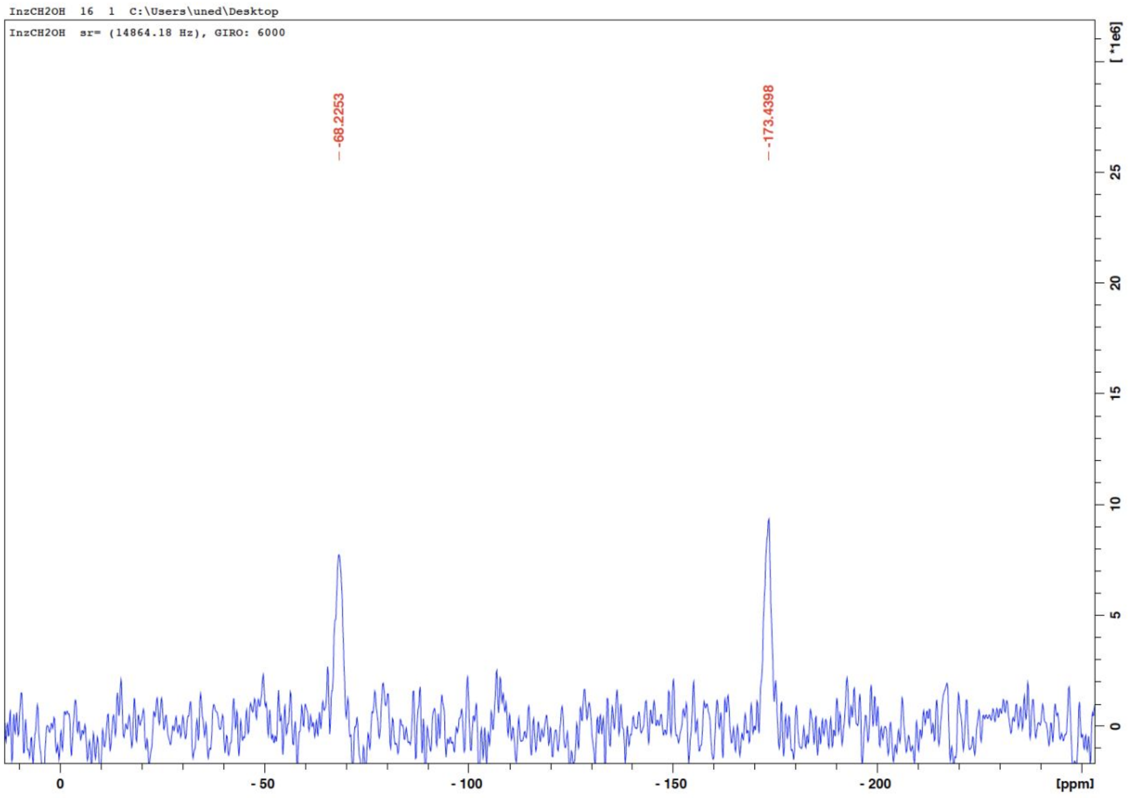

Figure S23. ^{15}N NMR spectrum (40.5 MHz, CPMAS) of 1-hydroxymethylindazole (**2a**)

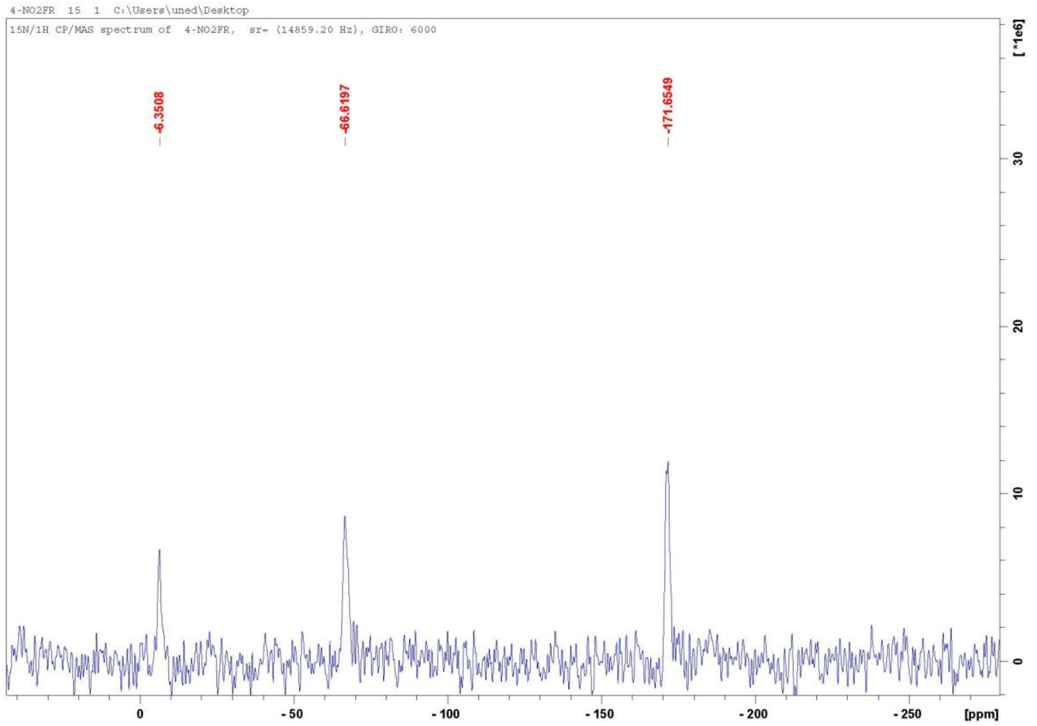

Figure S24. ^{15}N NMR spectrum (40.5 MHz, CPMAS) of 1-hydroxymethyl-4-nitroindazole (**2b**)

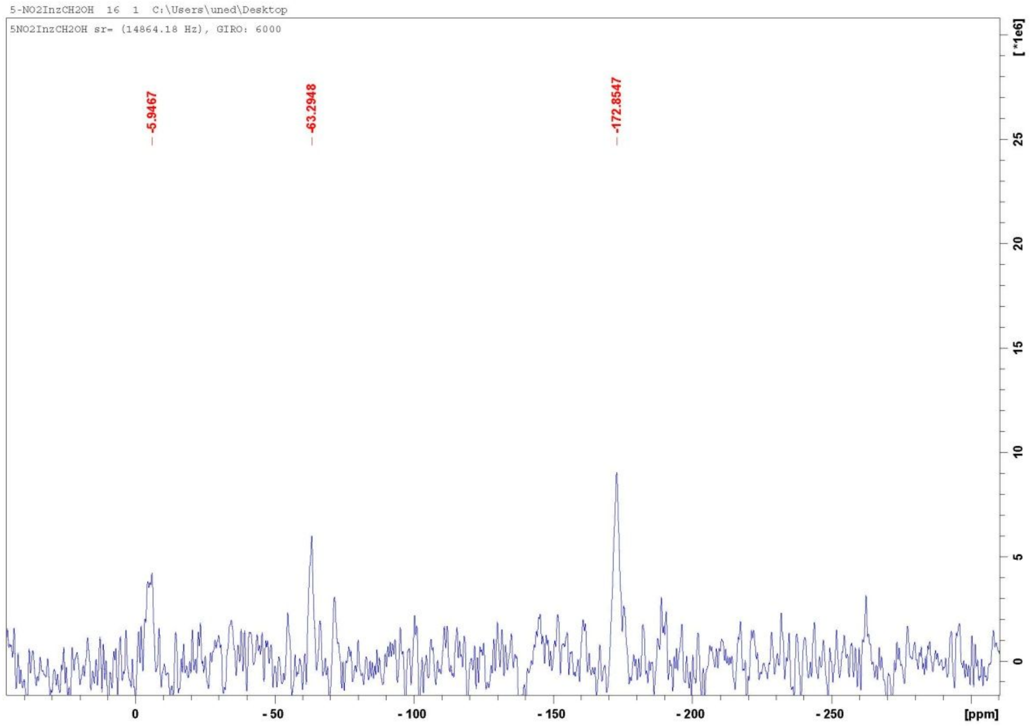

Figure S25. ^{15}N NMR spectrum (40.5 MHz, CPMAS) of 1-hydroxymethyl-5-nitroindazole (**2c**)

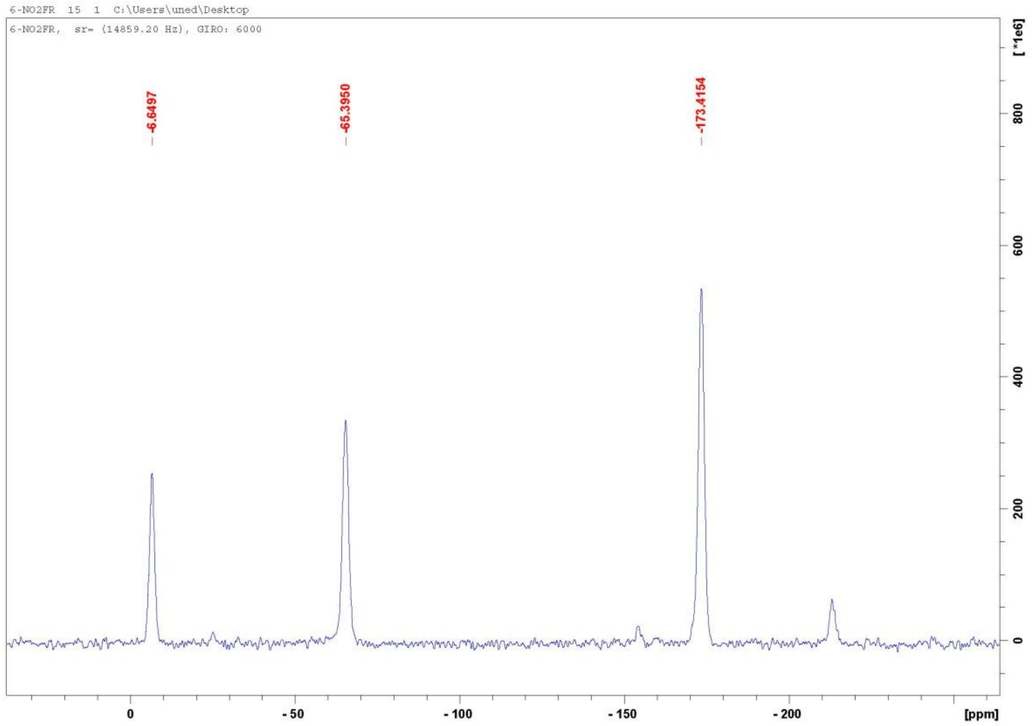

Figure S26. ^{15}N NMR spectrum (40.5 MHz, CPMAS) of 1-hydroxymethyl-6-nitroindazole (**2d**)

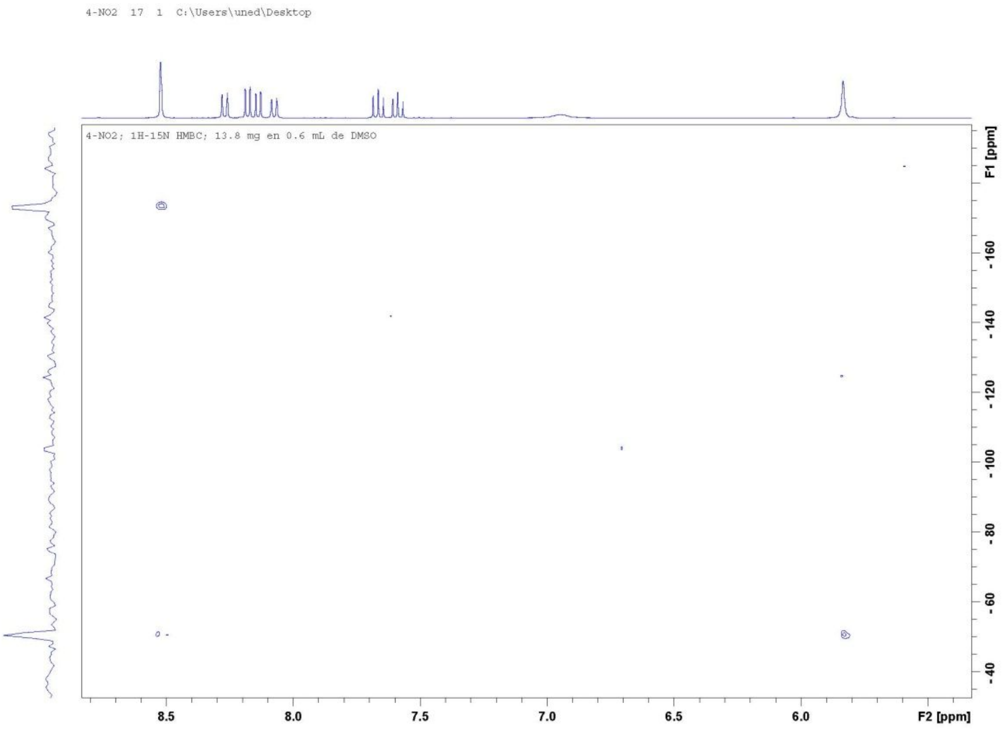

Figure S27. 2D(^{15}N-1H) HMBC NMR spectrum (40.5 MHz, DMSO-d_6) of 1-hydroxymethyl-4-nitroindazole (**2b**)

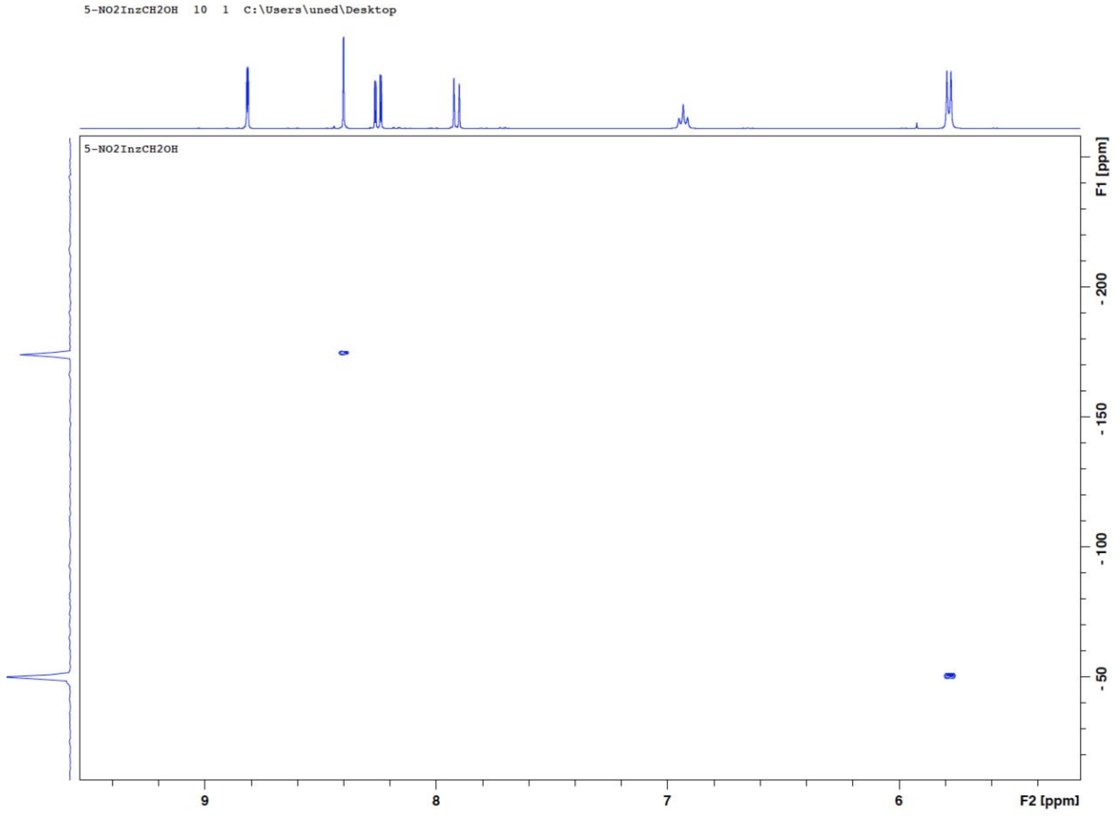

Figure S28. ^{15}N NMR spectrum (40.5 MHz, DMSO-d_6) of 1-hydroxymethyl-5-nitroindazole (**2c**)

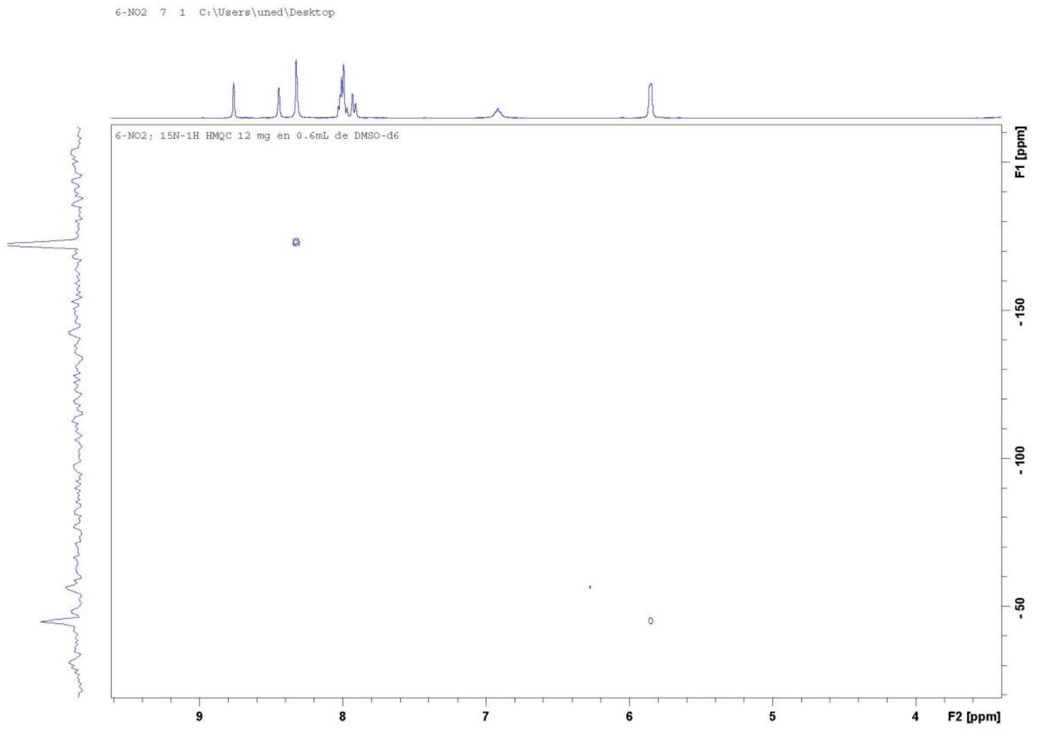

Figure S29. ^{15}N NMR spectrum (40.5 MHz, DMSO-d_6) of 1-hydroxymethyl-6-nitroindazole (**2d**)

3. Thermal ellipsoid plot for each crystal structure of full-page size.

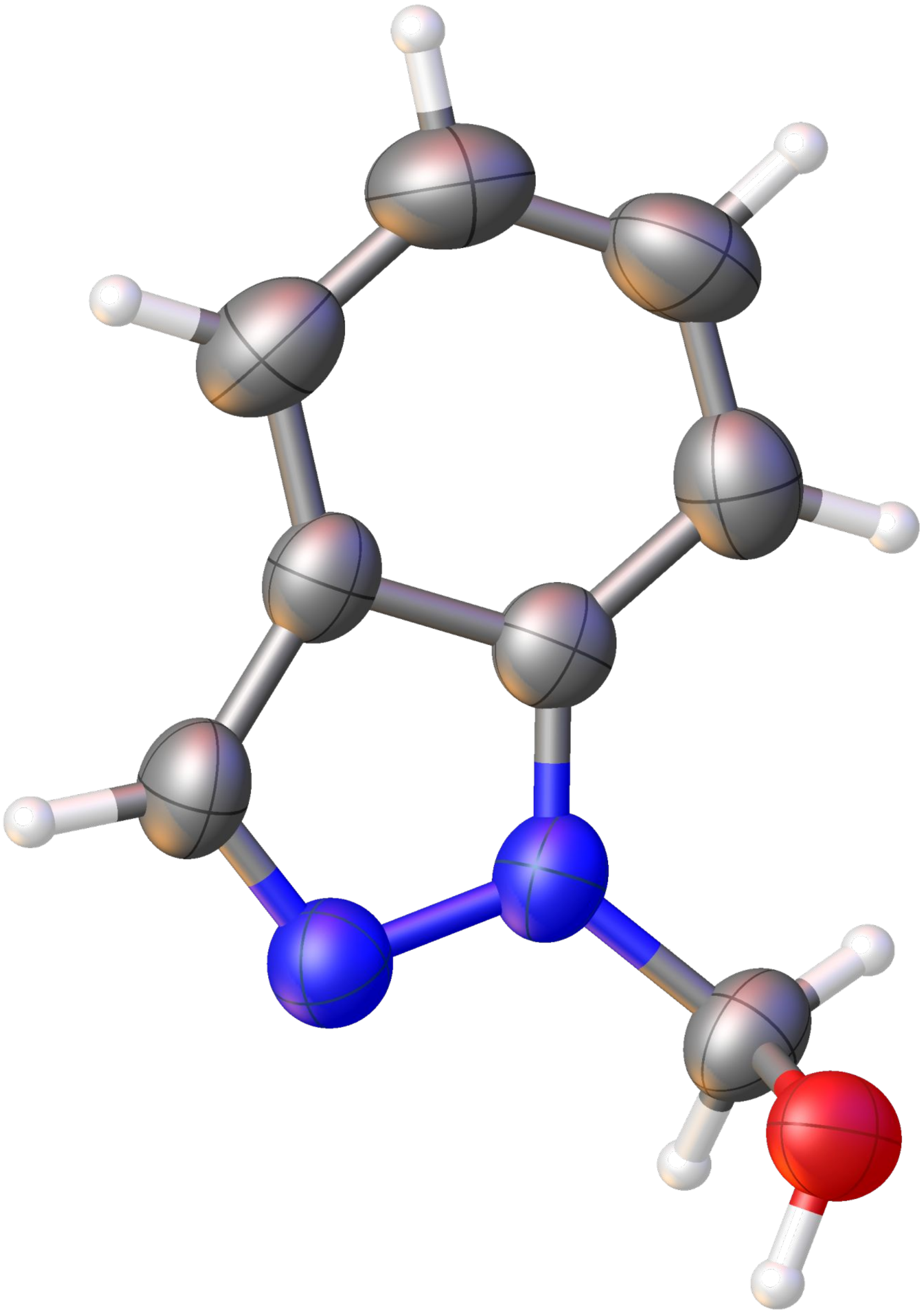

Figure S30. Structure representation of **2a**. No-hydrogen atoms are represented by their thermal ellipsoids with a contour probability of 50%.

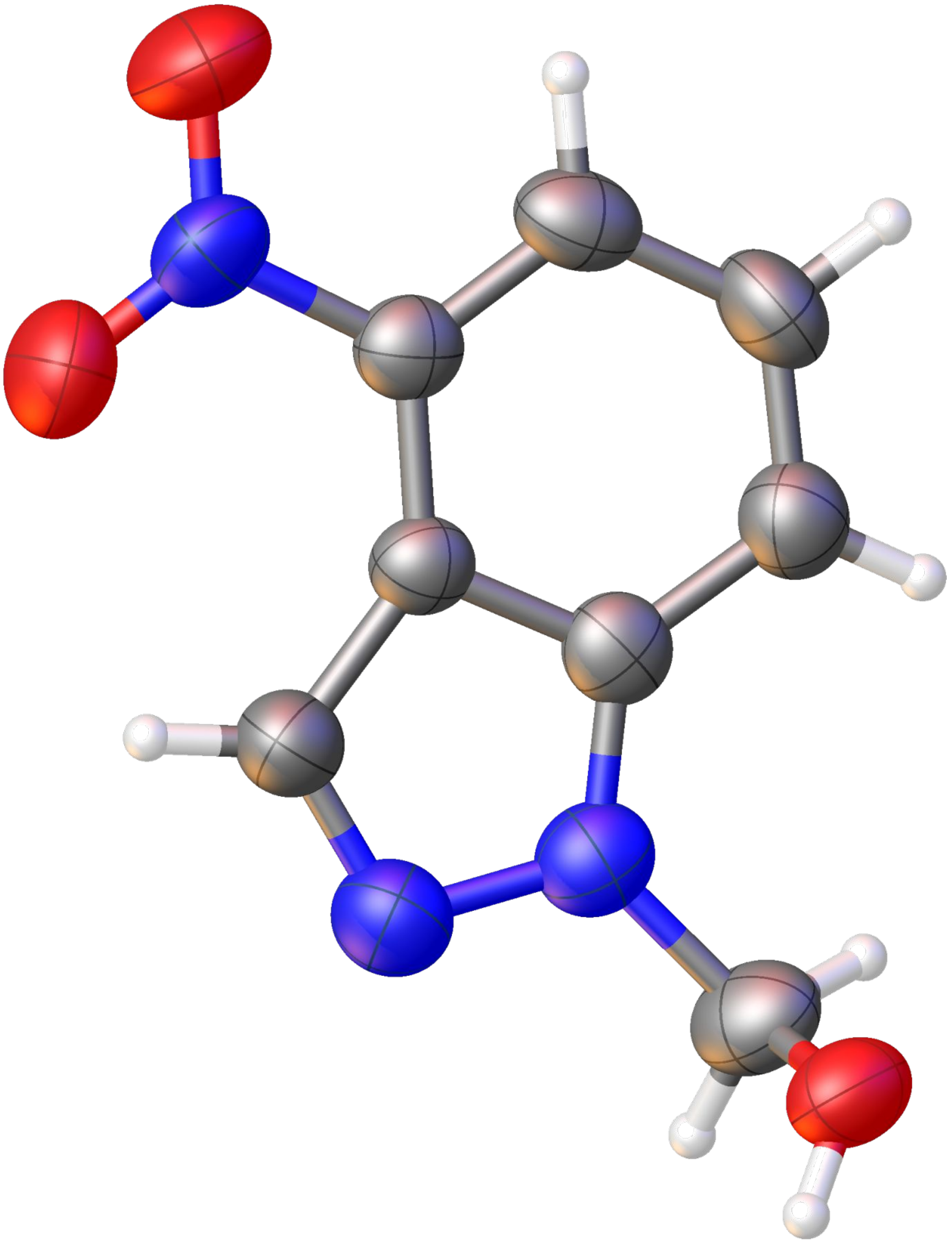

Figure S31. Structure representation of **2b**. No-hydrogen atoms are represented by their thermal ellipsoids with a contour probability of 50%.

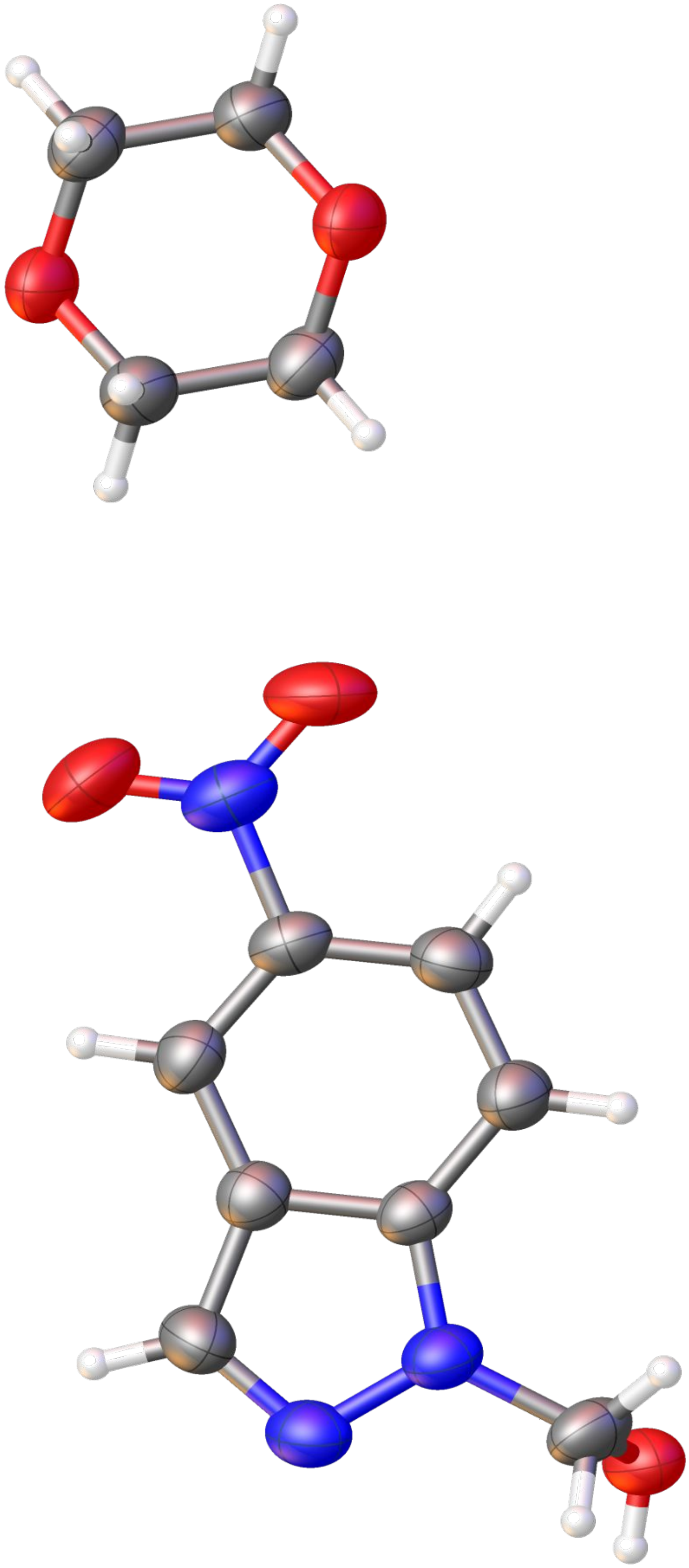

Figure S32. Structure representation of **2c** and a dioxane crystallization molecule. No-hydrogen atoms are represented by their thermal ellipsoids with a contour probability of 50%.

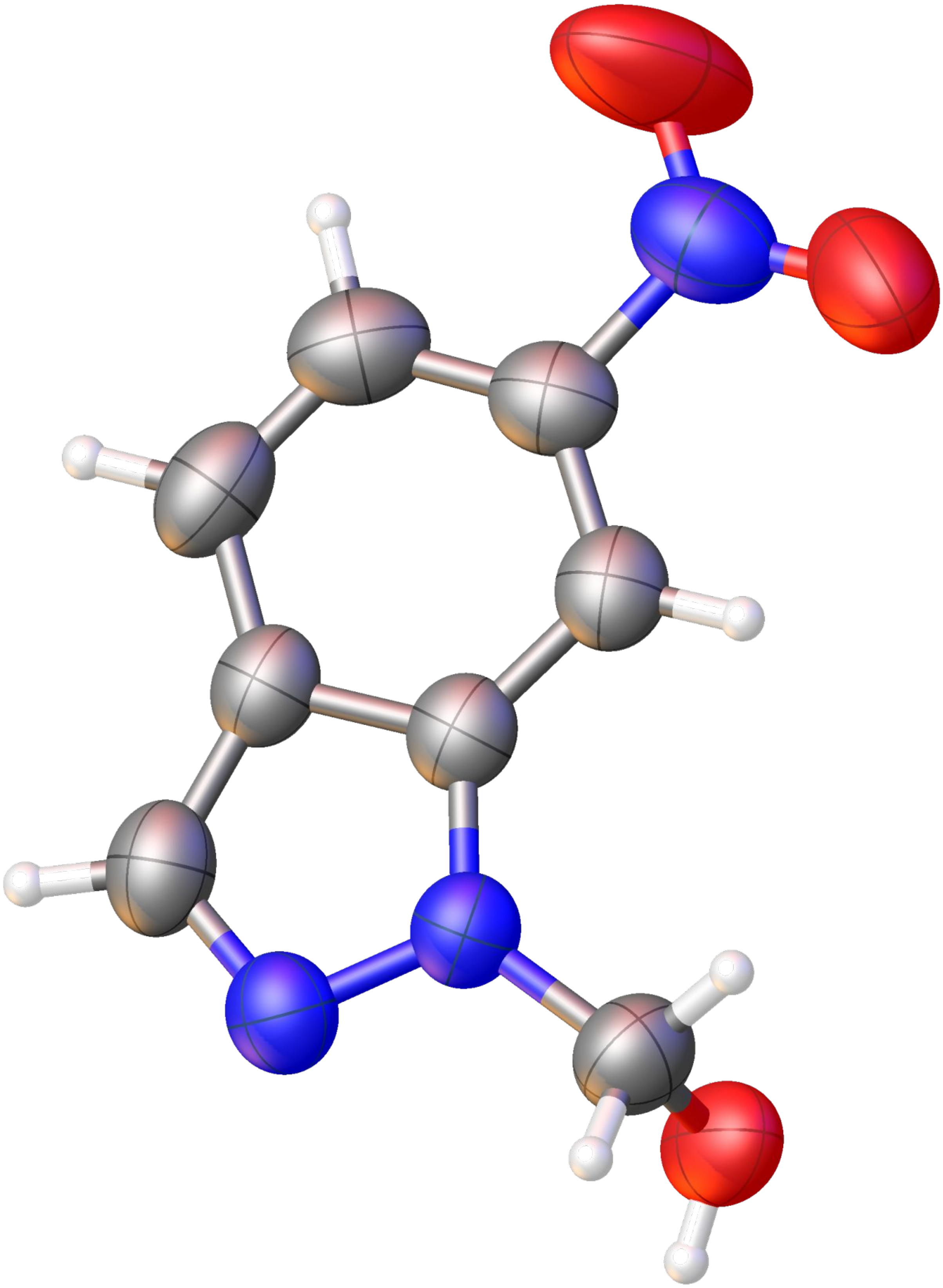

Figure S33. Structure representation of **2d**. No-hydrogen atoms are represented by their thermal ellipsoids, with a contour probability of 50%.

Table S1. Crystallographic data.

	**2a**	**2b**	**2c**	**2d**
Formula	$C_8H_8N_2O_1$	$C_8H_7N_3O_3$	$C_8H_7N_3O_3 \cdot 1/2\ C_4H_8O_2$	$C_8H_7N_3O_3$
Formula weight	148.16	193.17	237.2	193.17
Temperature (K)	250(2)	250(2)	250(2)	250(2)
Wavelength (Å)	1.54178	1.54178	1.54178	1.54178
Crystal system	Monoclinic	Monoclinic	Triclinic	Monoclinic
Space group	$P\ 2_1/c$	*P* 2(1)/*n*	P-1	$P\ 2_1/c$
a/Å	15.5674(13)	4.0291(4)	7.6799(4)	7.248(3)
b/Å	5.6287(5)	7.3233(8)	8.7337(4)	13.700(4)
c/Å	8.5729(6)	29.109(3)	9.0758(4)	9.233(4)
α/ º	90.00	90.00	109.272(2)	90.00
β/ º	100.514(7)	91.482(7)	107.976(2)	108.076(18)
γ/ º	90.00	90.00	93.100(2)	90.00
Volume /$Å^3$	738.58(11)	858.61(15)	538.45(5)	871.5(5)
Z	4	4	2	4
Calc. density / g cm^{-3}	1.332	1.494	1.463	1.472
μ/mm-1	0.744	1.004	0.981	0.989
Dimensions (mm)	0.3 x 0.2 x 0.06	0.1 x 0.1x 0.05	0.2 x 0.12 x 0.08	0.12 x 0.1 x0.06
Limiting indices *h*	-17< *h* < 17	-4< *h* < 3	-6 < *h* < 6	-8 < *h* < 8
k	-6< *k* < 6	-7< *k* < 8	-11<*k*< 12	16 <*k*< 16
l	-9< *l* < 8	-32< *l* < 22	-17< *l* < 16	-10< *l* < 10
F(000)	312	400	468	400
Reflections collected/unique with I > 2σ(I)	1150/818	4313/1279	5095/2565	1517/1189
Refined parameters	116	132	243	128
Goodness-of-fit on F^2	1.016	0.997	1.015	1.012
R_1	0.0729	0.0966	0.0614	0.0882
wR_2	0.2237	0.2719	0.1049	0.2328

4. Computational data including GIAO calculations, IRC profiles, and gas phase calculations, pages S26 to S72.

Electronic energy (Hartree), number of imaginary frequencies and optimized cartesian coordinates (Å) calculated at B3LYP/6-311++G(d,p) computational level in gas phase.

1a_1H
Total Energy= -379.943981804 Hartree
NIMAG= 0
C,-1.5820510944,0.409966527,0.
C,-0.4011046563,-0.3715627857,0.
C,0.8523357556,0.2651099099,0.
C,0.8887749606,1.647939086,0.
C,-0.3014666202,2.4106975446,0.
C,-1.5495993152,1.8117615195,0.
H,1.8426311009,2.1627126452,0.
H,-0.2330060558,3.4926409516,0.
H,-2.4578799396,2.4030767153,0.
N,-2.6151112243,-0.4862679245,0.
N,-2.1872183525,-1.7748502034,0.
C,-0.8703946101,-1.7214733951,0.
H,-0.297497301,-2.6369478818,0.
H,-3.6044552941,-0.3029933447,0.
H,1.7689572763,-0.3141012037,0.

1a_2H
Total Energy= -379.936161231 Hartree
NIMAG= 0
C,-0.2620332127,0.6878162547,0.
C,-0.2724620948,-0.7498144982,0.
C,0.9472008738,-1.4726890065,0.
C,2.1204142883,-0.7599778148,0.
C,2.1271040338,0.6680833026,0.
C,0.9649269684,1.3978128946,0.
H,3.0686794075,-1.2853773236,0.
H,3.0817329936,1.182351078,0.
H,0.9681694486,2.481092384,0.
N,-1.5110107699,1.1915184602,0.
N,-2.2898156999,0.0969560973,0.
C,-1.6279578995,-1.0825524144,0.
H,-2.1459254871,-2.0278382294,0.
H,-3.2905016141,0.2242654113,0.
H,0.952635234,-2.5568570159,0.

1b_1H
Total Energy= -584.505867850 Hartree
NIMAG= 0

C,-1.5700157524,0.4021398536,0.
C,-0.3955952978,-0.3936431083,0.
C,0.8400969927,0.2817182247,0.
C,0.8894118165,1.6632501024,0.
C,-0.2999303099,2.415289499,0.
C,-1.5416039623,1.8020758097,0.
H,1.8537538652,2.1518105966,0.
H,-0.2358877209,3.4964864451,0.
H,-2.4541598557,2.3863988086,0.
N,-2.6105549074,-0.4825417813,0.
N,-2.1942678201,-1.7687289248,0.
C,-0.8755031569,-1.7389196667,0.
H,-0.3049237599,-2.6513484921,0.
N,2.102175621,-0.474065693,0.
O,2.0224536488,-1.6981888535,0.
O,3.1535766047,0.1546575494,0.
H,-3.5984402255,-0.2878558792,0.

1b_2H
Total Energy= -584.501885362 Hartree
NIMAG= 0
C,-1.6003561784,0.3141889493,0.
C,-0.3538381066,-0.4038993017,0.
C,0.8491560681,0.3461087355,0.
C,0.8067182073,1.7197326386,0.
C,-0.4352080826,2.4060336397,0.
C,-1.6316171501,1.7281230371,0.
H,1.7382130431,2.2692388534,0.
H,-0.4269956503,3.4891379763,0.
H,-2.5818238048,2.2479835985,0.
N,-2.6511410608,-0.5272118721,0.
N,-2.0791520901,-1.7376057809,0.
C,-0.7263285742,-1.7460159943,0.
H,-0.1476617767,-2.6521037129,0.
N,2.1465224546,-0.3342666324,0.
O,2.1318858827,-1.56420832,0.
O,3.166336243,0.3454656334,0.
H,-2.6795277743,-2.5495072774,0.

1c_1H
Total Energy= -584.508366392 Hartree
NIMAG= 0
C,-1.5163634072,0.6867394503,0.
C,-1.1076299919,-0.6721166005,0.
C,0.2543863192,-0.9907026531,0.
C,1.1482074226,0.0647671461,0.
C,0.7457450467,1.4179115967,0.
C,-0.5940795757,1.7458863322,0.

H,1.5081622237,2.1840550153,0.
H,-0.912475384,2.7810884581,0.
N,-2.8769628861,0.6572836926,0.
N,-3.3735670133,-0.6097487426,0.
C,-2.3318442919,-1.412394168,0.
H,-2.4746083274,-2.4825025468,0.
H,-3.5219569165,1.4305783304,0.
H,0.615809573,-2.0099014573,0.
N,2.5921337833,-0.2356108749,0.
O,3.3713725292,0.7108996662,0.
O,2.9317473263,-1.4133895746,0.

1c_2H
Total Energy= -584.501307023 Hartree
NIMAG= 0
C,-1.5341060026,0.6955520826,0.
C,-1.0947285023,-0.6745814985,0.
C,0.2790552176,-0.9892203002,0.
C,1.1546307218,0.0688220355,0.
C,0.7415231318,1.4330823833,0.
C,-0.5891425942,1.7540261513,0.
H,1.5050593251,2.1978587105,0.
H,-0.922183594,2.784140321,0.
N,-2.873656315,0.7805429607,0.
N,-3.2710688868,-0.5057660628,0.
C,-2.2788421689,-1.4177007126,0.
H,-2.4720760668,-2.4779665554,0.
H,-4.2623940325,-0.6971624699,0.
H,0.6500285715,-2.0049047231,0.
N,2.6007153404,-0.2220008083,0.
O,3.3724154584,0.7306907258,0.
O,2.9520523563,-1.3973237199,0.

1d_1H
Total Energy= -584.506944649 Hartree
NIMAG= 0
C,1.1045736746,-0.6014276299,0.
C,1.5289011559,0.7517693601,0.
C,0.5707024397,1.781181473,0.
C,-0.7679266163,1.4439282389,0.
C,-1.1458096145,0.0852826173,0.
C,-0.2440834116,-0.9636598687,0.
H,0.872548382,2.8215738275,0.
H,-1.5417104996,2.1979947215,0.
H,-0.5909567506,-1.9875745531,0.
N,2.2499516003,-1.3477508571,0.
N,3.3638433033,-0.5800933094,0.
C,2.9538672324,0.6735791335,0.

H,3.6763715152,1.4755924033,0.
N,-2.5886250324,-0.243979745,0.
O,-2.9039239967,-1.4283955414,0.
O,-3.3844952672,0.6863894508,0.
H,2.3473589355,-2.3500915211,0.

1d_2H
Total Energy= -584.500094255 Hartree
NIMAG= 0
C,1.1312963409,-0.6161771281,0.
C,1.5428513757,0.7610817117,0.
C,0.5727196452,1.7957141278,0.
C,-0.7524788236,1.4489160196,0.
C,-1.1311408177,0.0754692463,0.
C,-0.2368630679,-0.9645069181,0.
H,0.8672836749,2.8382771132,0.
H,-1.5324669384,2.1964862616,0.
H,-0.568226297,-1.9932786653,0.
N,2.1890206012,-1.4509326549,0.
N,3.2378708457,-0.6198778256,0.
C,2.9357417075,0.6997042279,0.
H,3.6988901079,1.4605625769,0.
N,-2.5759460276,-0.2458404132,0.
O,-2.9022682651,-1.4262802186,0.
O,-3.3648065317,0.6924001691,0.
H,4.1638338901,-1.0227591504,0.

1e_1H
Total Energy= -584.510755036 Hartree
NIMAG= 0
C,-0.3521838959,-0.4199623078,0.
C,0.3824217447,-1.6351025115,0.
C,1.7824403117,-1.6102199469,0.
C,2.4350664504,-0.3836168764,0.
C,1.7132067426,0.8195360108,0.
C,0.3262525446,0.8117816538,0.
H,3.5171706071,-0.3460807568,0.
H,2.2219556837,1.7743013931,0.
N,-1.6599364273,-0.7723001578,0.
N,-1.8325818058,-2.1198838026,0.
C,-0.628154046,-2.6481753611,0.
H,-0.5074373762,-3.7210104831,0.
H,-2.4488700197,-0.1447454548,0.
H,2.3496016041,-2.533976523,0.
N,-0.4183281486,2.0615839836,0.
O,0.198154425,3.1179596475,0.
O,-1.6503954643,1.9735871428,0.

1e_2H
Total Energy= -584.492550050 Hartree
NIMAG= 0
C,0.0013149494,0.5872425495,-0.0089274812
C,1.4086724149,0.9083406999,0.0051207667
C,2.3977014508,-0.0985371902,0.0465590547
C,1.9930548698,-1.4123058554,0.0648635846
C,0.6177241459,-1.7526944186,0.0424301105
C,-0.3637886454,-0.790807368,0.0112973248
H,2.7258379179,-2.2090303029,0.0968995798
H,0.3126026362,-2.7903946789,0.0506964333
N,-0.7516110541,1.6969270184,-0.0566483901
N,0.1540785,2.6904443428,-0.0726331492
C,1.4431884281,2.3037739239,-0.0365548537
H,2.2590571903,3.007936042,-0.04339137
H,-0.191321498,3.6385321492,-0.1066161493
H,3.4502814602,0.1600839218,0.0620899786
N,-1.7681340487,-1.2267931481,0.0034625356
O,-1.9939983867,-2.4230196221,-0.1695893984
O,-2.6265023306,-0.3744220633,0.1759414234

2a
Total Energy= -494.510956924 Hartree
NIMAG= 0
C,-0.7836412304,0.5896167808,0.1618945665
C,0.3113454686,-0.3060412629,0.1873590217
C,1.613922967,0.1761751896,-0.0270344579
C,1.7827847891,1.5277820775,-0.2676777521
C,0.6761443811,2.4061630081,-0.3006797529
C,-0.6171603645,1.9585819158,-0.0907901607
H,2.7763095421,1.9254069581,-0.4387878945
H,0.8452227001,3.4580541185,-0.5013839792
H,-1.4591519738,2.6383567405,-0.1341959568
N,-1.9017028645,-0.1645653193,0.4273345223
N,-1.5868148488,-1.4840129998,0.5827147147
C,-0.2806619626,-1.5791707259,0.4528909384
H,0.2012452226,-2.5404473932,0.5560237405
C,-3.2906577116,0.2268285265,0.392559461
H,-3.8225451297,-0.4608656372,1.05375836
H,-3.379462649,1.2454234587,0.7690015608
O,-3.8457186664,0.2419602675,-0.9074606003
H,-3.859641555,-0.6621009319,-1.2421407652
H,2.4645561956,-0.4956426016,-0.0067958765

2b
Total Energy= -699.072901139 Hartree
NIMAG= 0

C,-0.7741778067,0.5700885766,0.1658521092
C,0.3178907618,-0.3327729672,0.1989158886
C,1.6039045801,0.1948170663,-0.0248858918
C,1.777503038,1.5427612883,-0.2774526792
C,0.6661377837,2.404503591,-0.3146595672
C,-0.6193727912,1.9367935652,-0.0978489413
H,2.7766478983,1.9183528703,-0.4487304147
H,0.8251384853,3.4553484519,-0.523288657
H,-1.4704656529,2.6046973879,-0.1436570581
N,-1.8940812499,-0.1765525336,0.4325143699
N,-1.5841827452,-1.4894885444,0.5965899369
C,-0.277352894,-1.6014772463,0.4723580367
H,0.2069619178,-2.5566406817,0.5807723265
C,-3.2845698366,0.227663725,0.394928713
H,-3.8242896951,-0.4848616015,1.0217760814
H,-3.371413945,1.2294558627,0.81458979
O,-3.8139371512,0.3019547803,-0.9095202139
H,-3.8706677676,-0.5895833766,-1.2724275844
N,2.7845312529,-0.6819991225,0.0006477864
O,2.593470404,-1.8736607231,0.2221082975
O,3.8836104133,-0.1792733688,-0.1985833284

2c
Total Energy= -699.075093422 Hartree
NIMAG= 0
C,-0.7816712701,0.5874458937,0.1581576303
C,0.3177669805,-0.307111266,0.1892093414
C,1.6159702309,0.1676108823,-0.0224755375
C,1.7577811324,1.5218255884,-0.2647618847
C,0.6662020721,2.4153847568,-0.3088808111
C,-0.6187138739,1.9583864065,-0.0999161229
H,0.8601508594,3.4587740373,-0.5138905015
H,-1.461253517,2.636056064,-0.1482830719
N,-1.8919446373,-0.1660411154,0.4204690944
N,-1.5743145808,-1.4864523019,0.5829223294
C,-0.2702401247,-1.5827340151,0.4580031179
H,0.2149805511,-2.5415765537,0.5646246323
C,-3.2867155443,0.2264147889,0.3922528873
H,-3.8142813788,-0.4742810059,1.0425255425
H,-3.3728463806,1.2371121617,0.7897584935
O,-3.8328642826,0.2657172928,-0.9060255077
H,-3.8934816407,-0.6348781681,-1.2449466934
H,2.4844037216,-0.4765353636,-0.0042418975
N,3.1123134127,2.0580676669,-0.4939470608
O,3.2211269444,3.2604930934,-0.7065966839
O,4.0526423456,1.272297607,-0.457996086

2d

Total Energy= -699.073550878 Hartree
NIMAG= 0
C,0.6475510668,0.1445196334,-0.2158675198
C,0.5684396282,1.5591192304,-0.2032760367
C,-0.6754014293,2.1898649928,-0.0247840542
C,-1.7986162117,1.4055170636,0.1435613494
C,-1.6758312812,0.0009469032,0.1328958829
C,-0.4764874647,-0.6659807113,-0.0401434058
H,-0.7554457988,3.2702180693,-0.0165164436
H,-2.7771184519,1.840596135,0.2869293167
H,-0.4425370032,-1.7459908362,-0.0254307252
N,1.9680578873,-0.1659642733,-0.4300485536
N,2.72883595,0.9575304979,-0.5189478209
C,1.9155715033,1.986833154,-0.397005161
H,2.3054699406,2.9921377257,-0.4545229521
C,2.6167408005,-1.4619279138,-0.3968154564
H,1.9417999635,-2.2001801804,-0.8283228682
H,3.5122346682,-1.3714384831,-1.0150713736
O,2.9104132023,-1.9091427829,0.9062830411
H,3.6057772338,-1.3530040208,1.2760190191
N,-2.8983925227,-0.812409029,0.3211242109
O,-2.7824539002,-2.0318526498,0.2961055
O,-3.9558337807,-0.2181085245,0.488824051

2e
Total Energy= -699.065765693 Hartree
NIMAG= 0
C,-0.8466088555,0.6198759037,0.2281344289
C,0.2690704549,-0.2639744088,0.2514597555
C,1.5690280861,0.181493396,-0.0163725844
C,1.7638836083,1.511883456,-0.3442224894
C,0.6765388279,2.3960165674,-0.3850226122
C,-0.6106937416,1.9773351396,-0.0837599078
H,2.7563483774,1.8844353913,-0.5643635422
H,0.8242837949,3.4380982574,-0.6351502001
N,-1.9487080676,-0.1553545898,0.5002651753
N,-1.5942341421,-1.467428369,0.6687938764
C,-0.2924314712,-1.541193248,0.5456677417
H,0.2131801045,-2.4890291884,0.6573906002
C,-3.3770039882,0.1067111367,0.3347226292
H,-3.874036828,-0.7183420647,0.8466469397
H,-3.6248829385,1.0561257446,0.7933860338
O,-3.7516203778,0.2032447691,-1.0204420484
H,-3.6873191715,-0.6693907353,-1.4250703155
H,2.4027557745,-0.5100460621,0.0225516024
N,-1.6483162238,3.013213799,-0.0461628687
O,-1.5306818523,3.9709433493,-0.7975991527
O,-2.5518315804,2.8782082061,0.7751010984

3a
Total Energy= -494.503307241 Hartree
NIMAG= 0
C,-0.6746011476,1.1259518971,0.8132268764
C,-0.0140687115,0.4690053285,-0.2808901918
C,1.2494460926,0.926849889,-0.7320175278
C,1.8094201636,2.0072625296,-0.0964437536
C,1.1503219753,2.6575062248,0.9905565887
C,-0.071851773,2.2377916986,1.4533828678
H,2.7737660319,2.3817578608,-0.4203582184
H,1.6364328896,3.5062015978,1.4588150578
H,-0.5709648699,2.7301477457,2.2792807336
N,-1.852924697,0.5407412257,1.0968746096
N,-1.9412349763,-0.4692275349,0.2071265258
C,-0.8885810574,-0.5566880166,-0.6403338607
H,-0.843477112,-1.31077172,-1.4094182885
C,-3.1712731666,-1.2488741991,0.1825346818
H,-2.9780713373,-2.1757827547,-0.3551022867
H,-3.4296533304,-1.4607299841,1.2228645592
O,-4.2118752605,-0.5914819681,-0.4945620979
H,-4.4281506535,0.2110232505,-0.004541599
H,1.7571899698,0.4390175496,-1.5563793061

3b
Total Energy= -699.068489258 Hartree
NIMAG= 0
C,-0.7168345687,1.1971481379,0.7679206115
C,0.1066419206,0.3324208754,-0.0326215312
C,1.4454080406,0.7255573704,-0.2811086663
C,1.9259724307,1.9060582805,0.2333222158
C,1.0939700657,2.7434789701,1.0202689279
C,-0.211471694,2.4084491167,1.2947661951
H,2.9504161577,2.1852540484,0.0280703808
H,1.5086590935,3.6655543952,1.4089942457
H,-0.8478920389,3.0454062002,1.8969219546
N,-1.9517415217,0.6861145965,0.922275835
N,-1.9137599418,-0.4707080214,0.2403135834
C,-0.7265752579,-0.7361657227,-0.3548327003
H,-0.5582454821,-1.6210172758,-0.9420667929
C,-3.1494565983,-1.2468670399,0.1376561237
H,-2.8892104886,-2.2483453055,-0.200031703
H,-3.5798852066,-1.2834441301,1.1412678783
O,-4.0379359896,-0.7095932975,-0.8022053538
H,-4.3435963697,0.1467583362,-0.4799212472
N,2.3286227655,-0.1206020926,-1.0881632039
O,1.8536812691,-1.1683566367,-1.5226310117
O,3.4790482945,0.2527629848,-1.2854270817

3c
Total Energy= -699.068336030 Hartree
NIMAG= 0
C,-0.6742398657,1.1342233386,0.8112766331
C,-0.011661787,0.4756333273,-0.2820046418
C,1.2460882374,0.920783204,-0.7347696182
C,1.789338363,2.0021145361,-0.084296239
C,1.1501658737,2.6696263862,1.0005100377
C,-0.0709111737,2.2466053278,1.4529056239
H,1.6558134403,3.5120703784,1.4506537866
H,-0.5696401661,2.7411168575,2.2768983405
N,-1.8497899824,0.5507055324,1.0890467844
N,-1.93633981,-0.4609570417,0.1969213649
C,-0.8870467414,-0.5516021389,-0.6456814461
H,-0.8374064512,-1.3046145762,-1.4153978554
C,-3.1680195064,-1.2488020901,0.1770561106
H,-2.9693927564,-2.1717349449,-0.3652107439
H,-3.4148841814,-1.4657397837,1.2188546045
O,-4.2086086684,-0.5920443271,-0.4916854974
H,-4.4611740125,0.1850129973,0.0214039362
H,1.7765771456,0.4525672293,-1.5524993991
N,3.1040318673,2.4983273244,-0.5309574338
O,3.5772091425,3.4644815783,0.0573143228
O,3.6520044929,1.9194938652,-1.4632471603

3d
Total Energy= -699.067065528 Hartree
NIMAG= 0
C,0.2132647575,-0.3004294157,-0.5465313895
C,0.2465140157,0.7365602183,0.4471724774
C,-0.9568886557,1.3458285854,0.8844254049
C,-2.1402073735,0.9207632091,0.339757214
C,-2.1428889241,-0.1090524729,-0.6440425505
C,-1.0098791816,-0.7307313246,-1.1047736928
H,-0.9470508679,2.1287715412,1.633151434
H,-3.0861334992,1.3491922505,0.6378453161
H,-1.0595627213,-1.5074572204,-1.8547059673
N,1.4507879025,-0.7460160387,-0.8351019324
N,2.2526680007,-0.0097417438,-0.0481979299
C,1.6016152608,0.8798551985,0.74029962
H,2.1305965432,1.5196954645,1.4276076986
C,3.6828053974,-0.316733719,-0.0476113631
H,3.9636089313,-0.4831584831,-1.0898434565
H,4.2125432113,0.5449724965,0.3549997062
O,3.9932494717,-1.4104724791,0.7707979752
H,3.6169119239,-2.2066999705,0.3769712967
N,-3.446865585,-0.531816323,-1.2024182338
O,-3.4499948947,-1.4185056617,-2.0474663525

O,-4.4516535531,0.0323810583,-0.7849463648

3e
Total Energy= -699.060538122 Hartree
NIMAG= 0
C,-0.7215320868,1.1262298234,0.822177711
C,-0.0315342116,0.4824101699,-0.2688341217
C,1.2293299176,0.9324549277,-0.7150679029
C,1.8034179217,2.0085954574,-0.0785570165
C,1.1489773825,2.651615195,1.0001409176
C,-0.0836245413,2.2362925198,1.447727157
H,2.769219613,2.3801675402,-0.3976496451
H,1.6128228757,3.4939298512,1.4956102745
N,-1.8879222477,0.5182820062,1.0816702435
N,-1.9443599064,-0.4865233565,0.1831154726
C,-0.8863581577,-0.5552947808,-0.6478788218
H,-0.8130430624,-1.3012902262,-1.4224589521
C,-3.1691764889,-1.2863922105,0.1532902934
H,-2.9725063506,-2.1826588652,-0.4329229271
H,-3.3936480874,-1.5501740007,1.1894205423
O,-4.2262786353,-0.6079055231,-0.4634745126
H,-4.4637849032,0.1487947223,0.0870289925
H,1.7320825663,0.4409143331,-1.540081983
N,-0.7072795143,2.9674267554,2.5587465116
O,-1.8830488234,2.733998024,2.8011690689
O,-0.0137421998,3.7746875573,3.1738746678

1aH⁺
Total Energy= -380.300739450 Hartree
NIMAG= 0
C,-0.6775264216,-0.2161325844,0.
C,0.7480996083,-0.2096819059,0.
C,1.4500390539,1.018630332,0.
C,0.7081739962,2.1745953311,0.
C,-0.7129368898,2.1437255533,0.
C,-1.43092396,0.9652881253,0.
H,1.2082113841,3.134884455,0.
H,-1.2518602676,3.0837493637,0.
H,-2.5132153879,0.9577092188,0.
N,-1.0717058752,-1.521919416,0.
N,0.0370580217,-2.3058914989,0.
C,1.140784424,-1.556285691,0.
H,2.1177139612,-2.0150242759,0.
H,-1.9976013144,-1.926717049,0.
H,2.532426535,1.0395144645,0.
H,-0.0405807481,-3.3154761827,0.

2aH⁺
Total Energy= -494.870217649 Hartree
NIMAG= 0
C,-0.8026508146,0.5874815423,0.0746682853
C,0.300451913,-0.3057660583,0.2122268173
C,1.621500212,0.1656573816,0.0360045623
C,1.7903377052,1.4971888732,-0.2581306908
C,0.6791583445,2.3736726793,-0.3801192232
C,-0.6235339491,1.9487071715,-0.2162419329
H,2.7869484833,1.8957039927,-0.4000247598
H,0.8635483786,3.4161214691,-0.6111355761
H,-1.4545311383,2.6350739369,-0.3118050206
N,-1.9426983383,-0.1373856968,0.2761204464
N,-1.5791423359,-1.4126179436,0.5714472655
C,-0.2560014125,-1.5586205686,0.5144724065
H,0.2005121393,-2.5240503851,0.6693909333
C,-3.3344878327,0.286367837,0.4596811262
H,-3.4830196863,0.629292,1.490297285
H,-3.5083730016,1.1076399065,-0.2413626774
O,-4.0974869739,-0.8543377164,0.1691243092
H,-4.9953599396,-0.7539997793,0.5064967103
H,2.4665125749,-0.5044858044,0.1316180206
H,-2.3143578283,-2.1081465077,0.6547931228

3aH⁺
Total Energy= -494.872386075 Hartree
NIMAG= 0
C,-0.7518709644,1.4203864672,0.5149759895

C,0.043358623,0.432592157,-0.1311794079
C,1.4247076178,0.6545065722,-0.3311667428
C,1.9510541901,1.8393846069,0.12630789
C,1.1409103542,2.8071740414,0.7776726816
C,-0.2116312773,2.6238294651,0.9860065399
H,3.0050593116,2.0475267538,-0.0079746686
H,1.6037064171,3.7247353471,1.1211369638
H,-0.8186488263,3.3720019765,1.4792225868
N,-2.0334166437,0.9467803879,0.5450678664
N,-2.049365174,-0.3059551089,0.0141688137
C,-0.8315364684,-0.6291956867,-0.4231849068
H,-0.6561599848,-1.5715238905,-0.9197704838
C,-3.3596454054,-0.9998587912,-0.1561762957
H,-3.8815952109,-0.5212707663,-0.9857855458
H,-3.1236195636,-2.0367497976,-0.4020114295
O,-4.1304824148,-0.8307112176,0.9824667472
H,-3.9637595858,-1.5277227006,1.6292434981
H,2.0411745517,-0.0847362313,-0.8268544968
H,-2.8266791061,1.2436924857,1.1012371505

1N: $1aH^{+}\cdot CH_2O\cdot H_2O$

Electronic Energy= -571.346431143 Hartree
NIMAG= 0
C,0.59925534,0.1658587963,0.9041101974
C,0.1526597188,1.0208321757,1.9557961011
C,1.0931847473,1.7140125812,2.7544191834
C,2.4246763904,1.5290762152,2.4755441614
C,2.8509754785,0.6724779385,1.4221024132
C,1.9647357988,-0.0175225241,0.6249590427
H,3.1743519744,2.0421548414,3.0647545017
H,3.9139333338,0.5615574656,1.2433273467
H,2.2998287357,-0.6658873167,-0.1740371484
N,-0.4980032884,-0.3662604852,0.3084362975
N,-1.5893063829,0.1212256451,0.9416054142
C,-1.2479141865,0.9518887205,1.9301875455
H,-1.9995447601,1.4300289338,2.5383812247
C,-1.6168038258,-1.034852381,-5.366462704
H,-1.7046949205,-2.042394689,-5.8077979765
H,-1.6858568225,-0.1778959084,-6.0559677748
O,-1.4519318701,-0.8683462594,-4.1795348756
H,-1.2764390742,-1.714392691,-2.6123109254
H,0.7681341574,2.3656364317,3.5556587823
H,-2.5135859438,-0.1581267568,0.6382311327
O,-1.175514852,-2.0128833686,-1.6847944777
H,-0.6249154289,-1.0300970018,-0.4913753808
H,-1.1446753196,-2.974978363,-1.6915450817

$2aH^{+}\cdot H_2O$

Electronic Energy= -571.352944936 Hartree
NIMAG= 0
C,0.6876051026,0.4324662486,0.0441055747
C,0.2357225092,0.5105699624,1.393801378
C,1.0336057833,1.1394266532,2.3775811128
C,2.2415374056,1.6601231609,1.982691231
C,2.6771384068,1.5717437075,0.6328688386
C,1.9236991861,0.969063812,-0.3522121037
H,2.8816252219,2.1481821141,2.7068131756
H,3.6398553353,1.9936755663,0.3697262258
H,2.2790821098,0.9111451345,-1.3723999088
N,-0.2771068181,-0.2115976362,-0.6785305913
N,-1.2860154701,-0.535269086,0.1738974973
C,-1.0131655682,-0.1285441744,1.4131800259
H,-1.7041799295,-0.3145859698,2.2207544206
C,-0.3377702409,-0.5960639092,-2.1064228888
H,-1.2364849958,-0.1410735095,-2.5331863871
H,0.541941924,-0.1476611416,-2.5639018106
O,-0.2899223172,-1.9693532868,-2.2883447814
H,-1.1859563833,-2.3315099703,-2.2531737681

H,0.6983117645,1.2037421759,3.4051307674
H,-2.0653668967,-1.1140548427,-0.165547216
O,-3.058131074,-2.3352531424,-1.1585071518
H,-3.1780918584,-3.2302156574,-0.8137205882
H,-3.8932871965,-2.111853209,-1.5901040516

TS (1N: 1aH$^{+}\cdot$CH$_2$O$\cdot$H$_2$O→ 2aH$^{+}\cdot$H$_2$O)
Electronic Energy= -571.302963240 Hartree
NIMAG= 1
C,-1.6526370836,0.8319819185,4.1740655283
C,-1.8516449061,1.762185831,5.2411734259
C,-0.7493083318,2.3580008271,5.8985428712
C,0.5106801255,2.0104827259,5.4766143561
C,0.7046345284,1.080600792,4.4175577499
C,-0.34842649,0.4840920218,3.7603109372
H,1.3792791272,2.4434342298,5.957078329
H,1.7183092644,0.8323339424,4.1253530391
H,-0.1767102345,-0.2287500953,2.9632308913
N,-2.8516366859,0.3843299551,3.6904713738
N,-3.7716452949,1.033354151,4.4596569407
C,-3.2412989187,1.8462926863,5.3839303901
H,-3.8616722386,2.4084919356,6.0647136219
C,-2.8947893741,0.7680530308,1.0850988386
H,-3.9370165714,0.6890263942,1.3858268888
H,-2.4462795056,1.7447684378,0.9045175676
O,-2.2204163163,-0.2431804231,0.8233796591
H,-2.6847876156,-1.1359879045,1.2630459736
H,-0.8978496642,3.0602873387,6.7095931158
H,-4.7542273539,0.8364403825,4.3314698883
O,-3.29033646,-1.9028077408,2.1995230237
H,-3.1639651119,-1.3513855599,3.0136260193
H,-3.0504385484,-2.8236757467,2.3603906806

1N: 1bH$^{+}\cdot$CH$_2$O$\cdot$H$_2$O
Total Energy= -775.895920661 Hartree
NIMAG= 0
C,0.6556957242,0.0945327076,0.8944806423
C,0.2512979734,0.9358882222,1.970180324
C,1.2589232671,1.5854806456,2.7231628132
C,2.5777144204,1.3947682138,2.407184808
C,2.944722587,0.5483874553,1.3264924
C,2.0096012215,-0.1079845065,0.5603925887
H,3.3358647158,1.8983500422,2.992615274
H,3.9974308877,0.4220090637,1.1058537293
H,2.2973243041,-0.7502756064,-0.2618190992
N,-0.4632483769,-0.4157197033,0.3245429733
N,-1.5247634473,0.0752151993,0.9989127662
C,-1.1517235085,0.8864836387,1.9904602309

H,-1.8660476123,1.3692486154,2.6356125983
C,-1.6796227608,-0.9172141216,-5.3037808377
H,-1.8608510565,-1.9051795955,-5.7599882298
H,-1.7234011837,-0.040826695,-5.9700619163
O,-1.4422347879,-0.7911222545,-4.123608242
H,-1.2547651872,-1.682849667,-2.603268544
H,-2.4634246131,-0.1870643,0.7222208105
O,-1.1359505418,-2.0058056794,-1.6845355681
H,-0.6175762379,-1.0675954018,-0.4893648032
H,-1.1320388469,-2.9682152449,-1.7084368142
N,0.8862141019,2.4680033488,3.8478649646
O,1.7818774427,3.0059970776,4.4689991385
O,-0.3148693149,2.5889927557,4.067788173

2bH$^+$·H_2O
Total Energy= -775.904147103 Hartree
NIMAG= 0
C,0.7071883484,0.432586911,0.0109923219
C,0.286475156,0.4044000958,1.3682471735
C,1.0917697789,1.0530897133,2.331819144
C,2.2441472568,1.6887035386,1.9478675891
C,2.6329322792,1.6997600548,0.5849313497
C,1.885451481,1.0849494867,-0.3963128047
H,2.8488815458,2.1814427388,2.6978406607
H,3.547145629,2.2122113425,0.3120168289
H,2.1997608853,1.1133819287,-1.4314991359
N,-0.2263069881,-0.2522719124,-0.7190399798
N,-1.1875001682,-0.6862114968,0.1359561965
C,-0.9189442538,-0.3255713932,1.3832933134
H,-1.5646997002,-0.5909232969,2.2034713765
C,-0.3300299992,-0.5505337772,-2.1606621536
H,-1.264077314,-0.1276023381,-2.5298440762
H,0.512857496,-0.0484479233,-2.6372277353
O,-0.3802330445,-1.923694708,-2.4031321356
H,0.5035440282,-2.3115768342,-2.4067591061
H,-1.970595432,-1.2751705518,-0.237483199
O,-3.031407726,-2.1870789425,-1.1285058117
H,-3.9487680599,-2.4509759165,-0.995476776
H,-2.6441812965,-2.7815333215,-1.78346584
N,0.6904759943,1.0482592547,3.7558182423
O,1.4120550167,1.6198188376,4.5490357877
O,-0.351316883,0.4595212897,4.0246084295

TS (1N: 1bH$^+$·CH_2O·H_2O→ 2bH$^+$·H_2O)
Total Energy= -775.854869350 Hartree
NIMAG= 1
C,-1.6436148695,0.8732359101,4.1811942152
C,-1.8579514983,1.7833872884,5.2612163212
C,-0.7340823871,2.3470732397,5.9106360113

C,0.5315933446,2.0146496569,5.5035749929
C,0.7226783049,1.1046501471,4.4307696002
C,-0.3360738195,0.52999419,3.7661326174
H,1.3800307557,2.4525137326,6.0125808835
H,1.7360068969,0.8590948432,4.1376663899
H,-0.1686745455,-0.1683716978,2.9554323281
N,-2.8331677998,0.4286314009,3.6796299841
N,-3.7583328806,1.0632266287,4.448597795
C,-3.2489797469,1.8645785476,5.3937938486
H,-3.8656694259,2.4182322952,6.0813853763
C,-2.9187209719,0.7408680915,1.0516566116
H,-3.954872371,0.6549511305,1.3705289827
H,-2.4838406377,1.7173876821,0.8396486877
O,-2.2337374588,-0.2676864604,0.8087804596
H,-2.6865219643,-1.1513689061,1.2454416562
H,-4.7406430357,0.8718540582,4.3049967925
O,-3.3015171599,-1.9445289757,2.1875023673
H,-3.1676002967,-1.4516288988,3.0265226609
H,-3.1298197695,-2.8867322528,2.30865297
N,-0.9219321852,3.2919518413,7.0294827889
O,0.0716402047,3.7540262841,7.5596336084
O,-2.0815566029,3.5439716235,7.3424924506

1N: 1cH^{+}·CH_2O·H_2O
Total Energy= -775.894674526 Hartree
NIMAG= 0
C,0.625802928,0.157675142,0.7739880842
C,0.2374777723,1.0289386618,1.8354775023
C,1.2118918406,1.7388066553,2.5688609388
C,2.515252501,1.5369656823,2.1985407609
C,2.9083704526,0.6730685827,1.1444191754
C,1.9752046803,-0.0275411318,0.4180291595
H,3.9645739858,0.5814713149,0.928254495
H,2.2665642668,-0.6878846325,-0.3879286746
N,-0.4998525104,-0.3856173184,0.2528173732
N,-1.5540954427,0.1112890079,0.9407797076
C,-1.1635250093,0.9578497278,1.8937269971
H,-1.8792296848,1.4452002147,2.5373437923
C,-1.7489169181,-1.0865481869,-5.3362445501
H,-1.8782453282,-2.0945725504,-5.7653859235
H,-1.8176523187,-0.2330420082,-6.0295425938
O,-1.5401703914,-0.9152348782,-4.1562927598
H,-1.3395445698,-1.7550264704,-2.6097904525
H,0.9648655934,2.4094256346,3.3809424972
H,-2.4945115322,-0.1750167436,0.6958790331
O,-1.214574896,-2.0463684886,-1.6811498868
H,-0.6691187722,-1.0657441697,-0.5354031632
H,-1.1825924336,-3.0086253841,-1.6763593986
N,3.5817433886,2.265154943,2.9430453423

O,3.2228680652,3.0024881898,3.8458142249
O,4.7329814724,2.0679202362,2.5920001992

2cH⁺·H₂O

Total Energy= -775.903324400 Hartree
NIMAG= 0
C,0.7164759167,0.4351229964,-0.0056809099
C,0.305630032,0.4154798903,1.3573932947
C,1.0811244764,1.0484307453,2.3480484748
C,2.2277056027,1.6685010425,1.9207832722
C,2.6414973524,1.6955712137,0.5684600956
C,1.895000133,1.0835480771,-0.4141972425
H,3.5605869641,2.2124293527,0.3254064156
H,2.214352463,1.1141511457,-1.4474425386
N,-0.2236443424,-0.250825184,-0.7229697381
N,-1.1839418805,-0.6790122783,0.1420038256
C,-0.9012937342,-0.3107535951,1.3828815715
H,-1.5504714308,-0.5776577341,2.2028244114
C,-0.3417008041,-0.5505066706,-2.163034328
H,-1.2759354441,-0.1223186403,-2.5257325703
H,0.5006690247,-0.0542744581,-2.6465184222
O,-0.4013924916,-1.9241555058,-2.4029054176
H,0.4807401184,-2.3154393225,-2.4214888941
H,0.8049786125,1.0572115662,3.3941386917
H,-1.9683638286,-1.2704915704,-0.22467235
O,-3.017825549,-2.1985234155,-1.1106064071
H,-3.9368683427,-2.4683694836,-1.0048163006
H,-2.6160727711,-2.7651012646,-1.7816573354
N,3.0829683151,2.3519505231,2.9322805256
O,4.0961150338,2.8929844961,2.5226996286
O,2.7043264646,2.3169555734,4.0908382072

TS (1N: 1cH⁺·CH₂O·H₂O→ 2cH⁺·H₂O)

Total Energy= -775.853862522 Hartree
NIMAG= 1
C,-1.6500893111,0.8712142214,4.169981952
C,-1.840524865,1.7786624902,5.259489078
C,-0.7409819104,2.3520509874,5.9315672804
C,0.5026410976,1.9914803926,5.4820206906
C,0.7151322863,1.0905543761,4.4068300291
C,-0.346552201,0.5231446986,3.7452647555
H,1.7349494066,0.861417951,4.1282397014
H,-0.1799470686,-0.1710344838,2.9314016528
N,-2.8486995185,0.4442669083,3.6803581653
N,-3.7620923067,1.0862065515,4.4645407743
C,-3.2300963517,1.8737738086,5.4064693921
H,-3.844223468,2.4267778102,6.1003768367
C,-2.9173850197,0.7392904364,1.0499830522

H,-3.9556548062,0.6437791971,1.3590138908
H,-2.4899577295,1.718738959,0.8364402322
O,-2.2188178004,-0.2630973476,0.821346417
H,-2.6668008785,-1.1488910222,1.2540957404
H,-0.8495419544,3.0388352923,6.7603516932
H,-4.7472726243,0.9060176919,4.3273512545
O,-3.2864864267,-1.9538008326,2.1882179943
H,-3.1714951305,-1.4663066633,3.031738921
H,-3.1026844835,-2.8941106106,2.3070842666
N,1.6944970403,2.5708910817,6.1569832

1N: 1dH^{+}·CH_2O·H_2O
Electronic Energy= -775.894132172 Hartree
NIMAG= 0
C,-0.0064548797,0.1710664231,0.6857759477
C,0.1453600146,0.7205532103,1.9912950299
C,1.3475777846,1.3785551262,2.3465633037
C,2.3383677411,1.4656222149,1.400322016
C,2.1390446233,0.9079738949,0.1120685733
C,0.9995877203,0.257495942,-0.2897173081
H,3.2788441953,1.9554405466,1.6112945537
H,0.8899850949,-0.1477914706,-1.2876893998
N,-1.234857862,-0.3962975612,0.604677449
N,-1.8427205985,-0.2187417922,1.7961753693
C,-1.0601027154,0.4412164936,2.6550838359
H,-1.3980765165,0.665662975,3.6546915672
C,0.6309575691,-1.0118816963,-4.2273644798
H,0.2186293336,-1.4336652251,-5.158433214
H,1.6407502342,-0.5745538129,-4.2679928634
O,0.0004895144,-1.0257787723,-3.1914477163
H,-1.5627912722,-1.4965405988,-2.3413321194
H,1.4820823703,1.8015385287,3.3337557751
H,-2.782431167,-0.5649251713,1.9468064252
O,-2.1876769016,-1.5656988791,-1.5934385851
H,-1.690954068,-0.8974245474,-0.2041205321
H,-2.8181759567,-2.2641953402,-1.7951010199
N,3.2440693067,1.034918845,-0.8874463015
O,3.0380415702,0.5831394319,-2.0046783202
O,4.267689265,1.5785661953,-0.5166730665

2dH^{+}·H_2O
Electronic Energy= -775.902362854 Hartree
NIMAG= 0
C,0.7065093061,0.4333293917,0.0006136568
C,0.2988973626,0.4171955276,1.3634867311
C,1.0832848643,1.0529167561,2.3502888856
C,2.2406962674,1.6803434268,1.953130768
C,2.6088321063,1.6731583632,0.5895983699

C,1.882281821,1.0732262264,-0.4133416159
H,2.8837889664,2.1855815369,2.6603540694
H,2.2292673237,1.1176404713,-1.4363005617
N,-0.2351892746,-0.2503079833,-0.721046106
N,-1.1925334935,-0.6750139689,0.1434952656
C,-0.909890494,-0.3074909056,1.385959817
H,-1.5616370287,-0.5749086337,2.2035500626
C,-0.3491684805,-0.5493358519,-2.1621067739
H,-1.2862200585,-0.1271070811,-2.5243662269
H,0.4898089522,-0.0457402847,-2.6438823569
O,-0.3978091936,-1.9231389442,-2.4039191592
H,0.4879359173,-2.3053342907,-2.4355322785
H,0.7819462989,1.0474878125,3.3900575756
H,-1.9765006868,-1.2673697657,-0.2230732078
O,-3.0088356094,-2.2042399274,-1.1205416736
H,-3.9267783254,-2.4829053798,-1.0296190167
H,-2.59203084,-2.7623338175,-1.7897343497
N,3.8762237292,2.3670416871,0.1968580481
O,4.5028960001,2.9136300613,1.085604146
O,4.1826006795,2.3297616738,-0.9837674691

TS (1N: 1dH^{+}·CH$_2$O·H$_2$O→ 2dH^{+}·H$_2$O)
Electronic Energy= -775.853885343 Hartree
NIMAG= 1
C,-1.6686951294,0.8958808987,4.1731859247
C,-1.8452487837,1.7909726669,5.273810193
C,-0.72924228,2.3364014793,5.9516334857
C,0.5259311363,1.9833196457,5.5222388235
C,0.6706850438,1.0940842289,4.428334219
C,-0.3771276332,0.5343693821,3.740343801
H,1.4171780928,2.3648264427,6.0003891695
H,-0.1875389701,-0.1431395972,2.9196013804
N,-2.8716715775,0.4856221442,3.6741211505
N,-3.7734848616,1.1280493245,4.4624209693
C,-3.2340628662,1.899693268,5.4169209868
H,-3.8461506128,2.4501308311,6.114421653
C,-2.9295837817,0.7264696073,1.0305173716
H,-3.9658999564,0.6034862043,1.3364091282
H,-2.5320283057,1.7142168138,0.7983153507
O,-2.1964925961,-0.256496479,0.8296645603
H,-2.6189814485,-1.1482309739,1.2682131258
H,-0.8585613187,3.0108863688,6.7887002102
H,-4.7608992095,0.9649918578,4.3191953005
O,-3.22682072,-1.9781222993,2.1964551937
H,-3.1493457634,-1.4996648056,3.0465749386
H,-3.007449896,-2.9114507472,2.3117464034
N,2.0522214233,0.7319614604,3.9939125684
O,2.9771738309,1.2186461946,4.6202266046
O,2.1569161134,-0.0254134972,3.0399736578

1N: $1eH^{+}\cdot CH_2O\cdot H_2O$
Electronic Energy= -775.902771046 Hartree
NIMAG= 0
C,1.2292259597,-0.0534580973,0.7051289372
C,0.345464628,0.8573846231,1.343477435
C,0.8307424495,1.7517558012,2.3221563682
C,2.1732916333,1.713791112,2.6348768727
C,3.0515260366,0.8068113702,1.9982852921
C,2.5941957828,-0.0730827294,1.0406028812
H,2.5740214595,2.3873224817,3.3812750666
H,4.1040611678,0.787727998,2.2533608786
N,0.5011162738,-0.777940792,-0.1738720406
N,-0.7871549382,-0.3691964096,-0.1200952255
C,-0.9205831617,0.6034588564,0.771404607
H,-1.8825996676,1.0572966141,0.9520615538
C,-3.2934475639,-0.4906284875,-5.2949292349
H,-3.9805832173,-1.3101420542,-5.5653362282
H,-3.0875897268,0.2678364079,-6.066958644
O,-2.7824497745,-0.4187521794,-4.1999868705
H,-2.6704735018,-1.2346459958,-2.6440672497
H,0.1633119324,2.4489262903,2.8133555004
H,-1.5153930672,-0.8277660151,-0.7481574706
O,-2.4745225929,-1.5609592127,-1.7389460319
H,0.8308793983,-1.5104765015,-0.7922245181
H,-3.076767532,-2.2853954765,-1.5415331219
N,3.495421414,-1.015925623,0.3746245543
O,4.668846157,-1.0139490394,0.6813602167
O,2.9746743712,-1.7552811818,-0.4668991877

$2eH^{+}\cdot H_2O$
Electronic Energy= -775.902387598 Hartree
NIMAG= 0
C,0.6717926268,0.4290088522,-0.0319813032
C,0.2755723517,0.471441355,1.3388969086
C,1.0586916567,1.1151330078,2.3193675793
C,2.2305400805,1.7185408159,1.9253638407
C,2.6545466599,1.6507455637,0.5817978809
C,1.9141501081,1.0038862203,-0.3878039832
H,2.8555832497,2.2277438507,2.6472113527
H,3.601481203,2.0858325023,0.2869538407
N,-0.3210888921,-0.2083347808,-0.7243516519
N,-1.2666921938,-0.5946203376,0.1766199122
C,-0.9498881403,-0.2182171641,1.40328481
H,-1.5850562736,-0.4541382965,2.2432719537
C,-0.6282194311,-0.4242159141,-2.1705594085
H,-1.6982162031,-0.251290384,-2.2750787237
H,-0.0741548685,0.3220751372,-2.7303800667
O,-0.3699752,-1.7273008238,-2.5654081787

H,0.5708211333,-1.8209408215,-2.7709825033
H,0.7386388043,1.1290703759,3.3539745023
H,-2.0251984515,-1.2300373996,-0.1632251739
O,-2.8466423803,-2.3596014953,-1.1085134407
H,-3.6978783623,-2.8068701164,-1.0485795785
H,-2.3085391459,-2.8313782541,-1.7596681816
N,2.514371812,0.8450809582,-1.722025781
O,3.385027928,1.620318012,-2.0545809766
O,2.1083430787,-0.1004824232,-2.4008463194

2N: 1aH^{+}·CH_2O·H_2O
Electronic Energy= -571.348437160 Hartree
NIMAG= 0
C,0.8518122992,1.4109762199,0.4363854123
C,1.7631582493,0.5378156822,-0.2198863536
C,2.9909364375,1.0374050302,-0.7085300086
C,3.2562053745,2.3741464013,-0.5246567432
C,2.3318646695,3.2280466109,0.1331614616
C,1.1243613915,2.7738224963,0.6228178117
H,4.1871634079,2.7928708635,-0.8856672722
H,2.58627433,4.2742992012,0.2541239618
H,0.4285029988,3.4352054133,1.1229370435
N,-0.2274365917,0.6574844541,0.794043385
N,-0.0298534983,-0.6210774219,0.3923305632
C,1.1460834103,-0.7287621003,-0.2145490327
H,1.4764972888,-1.6795624586,-0.602821214
C,-4.8277790623,-3.3082485563,-1.4244675062
H,-5.3134754184,-4.0789933156,-0.8020458606
H,-5.2752169869,-3.1044211558,-2.4106098584
O,-3.8465014333,-2.7076878972,-1.0494624408
H,-2.7083461363,-2.5282560613,0.3044483444
H,3.6954017034,0.3856599101,-1.2098647656
H,-1.0825515801,0.9171088912,1.2641232992
O,-2.0083876456,-2.2975610965,0.9511324732
H,-0.7678900715,-1.3509228061,0.5854333962
H,-2.0468661362,-2.9387293048,1.6681079032

3aH^{+}·H_2O
Electronic Energy= -571.350016100 Hartree
NIMAG= 0
C,0.3902440537,1.4383700444,0.044095201
C,1.1393074984,0.3617664136,-0.4997526632
C,2.5078750677,0.533887993,-0.7995898071
C,3.0699492006,1.7623182763,-0.5358662374
C,2.304961918,2.8204700062,0.0178580116
C,0.9626700383,2.6861371328,0.31771881
H,4.1169111649,1.9329445679,-0.752743216
H,2.791746201,3.7692907276,0.209653376

H,0.389311202,3.5039902979,0.7350639284
N,-0.8943078119,0.9877001213,0.204260239
N,-0.9474142235,-0.3242109043,-0.1580840146
C,0.2372014507,-0.7167407456,-0.6124802168
H,0.3561757669,-1.7177447741,-0.9960352068
C,-2.2602866085,-1.0799471631,-0.2257552058
H,-2.7833528835,-0.8652995676,0.7119146243
H,-2.8056928228,-0.6582438063,-1.0735383465
O,-2.0048845845,-2.4038211278,-0.4335648039
H,-2.0664690975,-2.9052894284,0.4097305609
H,3.0901194631,-0.2750158525,-1.2226635735
H,-1.64910368,1.4001558123,0.7344469763
O,-2.381097876,-3.6476588163,1.9453682992
H,-1.7119273381,-4.0617749227,2.5021641232
H,-3.1755960989,-4.1861942846,2.0407781416

TS (2N: 1aH^{+}·CH_2O·H_2O→ 3aH^{+}·H_2O)
Electronic Energy= -571.306636274 Hartree
NIMAG= 1
C,0.3240905269,1.90651947,0.9249087674
C,1.0141532524,0.9232498049,0.1720382913
C,2.2375253037,1.24564367,-0.4540359986
C,2.721876268,2.5259393412,-0.300368018
C,2.0175567408,3.4931330094,0.4608947356
C,0.8168726023,3.2091765232,1.0835490537
H,3.658723954,2.807400946,-0.7648578851
H,2.4369508417,4.487738939,0.5560211518
H,0.2889989782,3.9572046389,1.6618915612
N,-0.8121181622,1.3012204948,1.389496609
N,-0.8802291667,-0.0067386206,1.0118308688
C,0.2057928922,-0.2340679196,0.2671362623
H,0.3713021305,-1.2124103078,-0.1612156003
C,-2.8034095187,-0.9506388951,-0.4852476869
H,-3.2678647052,-0.2834972812,0.2364434913
H,-2.6033275962,-0.613799001,-1.5017955575
O,-2.5920304105,-2.1479166086,-0.2177526436
H,-2.6435695417,-2.3152390548,0.8445763653
H,2.7781580386,0.5070882732,-1.0332907746
H,-1.4845776294,1.6686549532,2.044501195
O,-2.4401342354,-2.1057124595,2.2063846085
H,-1.7142469068,-1.4481098555,2.2324401345
H,-2.2825390764,-2.8096333001,2.847704099

2N: 1bH^{+}·CH_2O·H_2O
Electronic Energy= -775.897760295 Hartree
NIMAG= 0
C,0.8517654584,1.3999669862,0.4374957355
C,1.7219324217,0.5188623851,-0.2569047273

C,2.9313728514,1.0462221529,-0.7637141353
C,3.2329417061,2.3713221498,-0.5781015816
C,2.3376586841,3.2198259446,0.1209233949
C,1.1452201307,2.7600167655,0.63651761
H,4.1631558226,2.7585190573,-0.9727049051
H,2.6062630816,4.2609602065,0.250016076
H,0.4689057602,3.4165393937,1.169104756
N,-0.2274794035,0.6626280047,0.8220832948
N,-0.0654415496,-0.6119449313,0.4007742388
C,1.0845873853,-0.7403464566,-0.2466331498
H,1.397334417,-1.6839312672,-0.6606390246
C,-4.7676154843,-3.297057988,-1.4452073404
H,-5.2012000752,-4.1336417199,-0.8716590544
H,-5.2242522204,-3.0667168475,-2.4209367828
O,-3.8303747457,-2.6536479381,-1.0292784059
H,-2.7207358249,-2.493353668,0.3282749247
H,-1.0607604939,0.9337648538,1.3252066868
O,-2.0267330174,-2.260285911,0.9830660433
H,-0.815011669,-1.3388209687,0.6098407331
H,-2.0319927792,-2.9312245052,1.6737207255
N,3.8711039075,0.1697194612,-1.4942334432
O,3.5340755934,-1.0030765342,-1.6206411307
O,4.898998423,0.6645746648,-1.9138840782

3bH^{+}·H$_2$O
Electronic Energy= -775.903212671 Hartree
NIMAG= 0
C,0.2777429242,1.2867515235,0.0012529646
C,1.111094559,0.263953096,-0.5233649553
C,2.4809337539,0.5542068143,-0.7171419691
C,2.9701470565,1.7935535982,-0.3935465655
C,2.1086449668,2.7889207962,0.1341704696
C,0.7649602698,2.5615690829,0.3391185543
H,4.0212356999,2.0002545615,-0.5469976011
H,2.5274296696,3.7570950095,0.3793673308
H,0.1163391631,3.3309437417,0.7379526841
N,-0.9837388317,0.7786152604,0.0791512225
N,-0.9566559444,-0.5129320341,-0.3387307544
C,0.274662836,-0.8528549972,-0.7136813195
H,0.5013412425,-1.8353421629,-1.0925257223
C,-2.2075302318,-1.3410280783,-0.352103576
H,-2.8940000942,-0.8593852174,-1.0543020588
H,-1.8964589095,-2.3098554583,-0.7400913388
O,-2.7252520205,-1.4955213279,0.9136221631
H,-3.3153437576,-0.7567123352,1.1251198241
H,-1.8248288483,1.1473867358,0.5361306162
O,-3.5225221703,1.290253128,1.3526822502
H,-3.5794222256,1.4627048501,2.3022210481
H,-4.2597962448,1.7746777731,0.9581179854

N,3.3837419679,-0.4784606579,-1.2680009102
O,2.8708747504,-1.5601934756,-1.5367440598
O,4.5546233992,-0.1884673164,-1.4142735117

TS (2N: 1bH$^+$·CH_2O·H_2O→ 3bH$^+$·H_2O)
Electronic Energy= -775.859996944 Hartree
NIMAG= 1
C,0.3310641742,1.9853447634,0.8794730916
C,0.9848394583,0.9666785748,0.1458929213
C,2.2444272737,1.2640427007,-0.4166311506
C,2.8049695035,2.50768109,-0.241242739
C,2.1221601267,3.4984777351,0.5018350252
C,0.8862743793,3.2609005708,1.0702237925
H,3.7720352422,2.7154223399,-0.6792765654
H,2.5873467022,4.4687979098,0.6234007722
H,0.3718826117,4.027616532,1.6363359594
N,-0.8515691153,1.4450217121,1.3015531122
N,-0.9870807621,0.1520759179,0.9054795304
C,0.1034665977,-0.1432522462,0.2010230824
H,0.2384494898,-1.1173862775,-0.2396315809
C,-2.9500350383,-1.0591370105,-0.4817418538
H,-3.4180745231,-0.4221701125,0.2655733401
H,-2.8825947886,-0.7421561229,-1.5225156136
O,-2.5479554595,-2.1983135542,-0.1982475185
H,-2.516794956,-2.3601128186,0.8661005991
H,-1.5480150969,1.8660903335,1.8965106271
O,-2.3248842278,-2.2299735674,2.2324917139
H,-1.647112899,-1.5498356784,2.3826057738
H,-2.170876005,-2.9804254977,2.8210945154
N,2.965038388,0.2401948183,-1.1973284212
O,2.4002818366,-0.8417100515,-1.3372577068
O,4.0583010879,0.5284909292,-1.6487390166

2N: 1cH$^+$·CH_2O·H_2O
Electronic Energy= -775.896659161 Hartree
NIMAG= 0
C,0.8411401901,1.4104546257,0.4428381523
C,1.742895682,0.5320626451,-0.2197606416
C,2.9661673072,1.0173843409,-0.7210779107
C,3.2154437832,2.3533182916,-0.5304093484
C,2.3210306819,3.2302027026,0.1282091732
C,1.1202657234,2.7744075857,0.6254244092
H,2.6060200546,4.268941308,0.2308042543
H,0.4324868095,3.4402613106,1.1302113126
N,-0.2350724052,0.6617889499,0.8088657411
N,-0.0456560572,-0.6192762634,0.4080226063
C,1.1216345689,-0.7346958243,-0.2079547467
H,1.4469273151,-1.6865069264,-0.5986310825

C,-4.7760875937,-3.2849150181,-1.438636963
H,-5.2674476156,-4.0574914119,-0.8234875631
H,-5.2109142841,-3.0798461547,-2.4297911051
O,-3.8002843937,-2.6825618892,-1.0504556588
H,-2.6970135109,-2.5147861683,0.3092844199
H,3.6838233291,0.3890794131,-1.2314350282
H,-1.0846555976,0.9246324635,1.2887445428
O,-2.0024709918,-2.2822091906,0.9638618236
H,-0.7916768457,-1.3513899375,0.6089138561
H,-2.0360525611,-2.9282126309,1.6770412478
N,4.4974583504,2.912954946,-1.0444989182
O,4.6936140687,4.1026443271,-0.8621147736
O,5.2528498225,2.1390065555,-1.6086158791

3cH$^{+}\cdot$H$_2$O
Electronic Energy= -775.901965597 Hartree
NIMAG= 0
C,0.271696942,1.3100149335,-0.0007812881
C,1.1145208906,0.289180454,-0.5222134054
C,2.487205437,0.5322442305,-0.7256565041
C,2.9386104408,1.7833583086,-0.3894059553
C,2.1058924922,2.8011085004,0.1341541909
C,0.7612426428,2.5823855563,0.3365571519
H,2.554904041,3.7570919802,0.3694980687
H,0.1198597807,3.3589614805,0.7320850274
N,-0.9864080211,0.7957368932,0.0688103031
N,-0.9535334417,-0.4988905921,-0.3507355125
C,0.2818748526,-0.8294255418,-0.7166810893
H,0.5018065125,-1.8168356874,-1.0927316339
C,-2.2029164985,-1.3288265224,-0.365117584
H,-2.8975687639,-0.8345086883,-1.0502218495
H,-1.8964744556,-2.2899451891,-0.7757355477
O,-2.7061381926,-1.5094200103,0.9025434765
H,-3.2926625907,-0.7739763465,1.1360339571
H,3.1651233188,-0.2117887553,-1.1222008259
H,-1.8314668237,1.1568751181,0.5246536363
O,-3.5224837048,1.2651883255,1.363989306
H,-3.5636327072,1.4454937237,2.3129663964
H,-4.2717507659,1.739386305,0.9795390847
N,4.3842127116,2.0895133957,-0.5849606253
O,4.7591287922,3.2064344681,-0.2704410662
O,5.0807728812,1.2000046801,-1.0439398718

TS (2N: 1cH$^{+}\cdot$CH$_2$O$\cdot$H$_2$O$\rightarrow$ 3cH$^{+}\cdot$H$_2$O)
Electronic Energy= -775.858196877 Hartree
NIMAG= 1
C,0.3273177268,1.967490535,0.8776285839
C,1.0091242197,0.9749541086,0.129321883

C,2.252492074,1.2627784637,-0.4596619847
C,2.7466596217,2.5315589545,-0.2637240177
C,2.0723404002,3.523089319,0.4845977443
C,0.8515999179,3.2561542651,1.0673175273
H,2.5377203833,4.4941527517,0.5870005571
H,0.3301422868,4.0116502198,1.6411377422
N,-0.8314857841,1.3862504725,1.3032116283
N,-0.9311897238,0.0869705252,0.8971068623
C,0.163163167,-0.1625872094,0.1830491293
H,0.3147664589,-1.1361641811,-0.2608000613
C,-2.9616203403,-1.0387293073,-0.4613473663
H,-3.3923497707,-0.3790076957,0.2885944851
H,-2.8914787507,-0.7265120668,-1.503375849
O,-2.6141313361,-2.1964844065,-0.1792988134
H,-2.5763293015,-2.3504742492,0.8873460732
H,2.8111479398,0.5405960932,-1.0396879766
H,-1.5335607742,1.7767658275,1.9123499478
O,-2.3514069054,-2.1925797438,2.243277585
H,-1.6391399489,-1.5389569052,2.3519338407
H,-2.2274470514,-2.9245515237,2.861308912
N,4.0590981132,2.8789059328,-0.869079572
O,4.4802355641,4.008370661,-0.676384865
O,4.6227208835,2.0098545693,-1.5164748655

2N: 1dH^{+}·CH_2O·H_2O
Electronic Energy= -775.895207702 Hartree
NIMAG= 0
C,0.8591595428,1.3826577231,0.4633573426
C,1.7693456699,0.5165960766,-0.2016048481
C,2.9950955565,1.0189522386,-0.6900405817
C,3.2683918369,2.3534968621,-0.5013543173
C,2.3331394983,3.1769667191,0.1650720988
C,1.1255407626,2.742807714,0.6616811701
H,4.1894408753,2.7980904593,-0.8519917356
H,0.4532856387,3.4255899294,1.1631537253
N,-0.2196181218,0.6285466961,0.8188476528
N,-0.0221944112,-0.6446340342,0.4081247132
C,1.1498244148,-0.7518209868,-0.2028398175
H,1.4778150421,-1.7003887741,-0.5988482287
C,-4.8067354032,-3.2237779273,-1.4311772009
H,-5.3102383867,-3.9880329446,-0.8154525945
H,-5.2498551888,-2.9982918512,-2.4141906845
O,-3.8094477784,-2.6515593535,-1.0521099322
H,-2.6859959792,-2.523741959,0.2950491202
H,3.6998520614,0.3733739474,-1.1983030363
H,-1.0724764001,0.8857376515,1.2962344016
O,-1.9808325939,-2.3109726964,0.9449945292
H,-0.7672182835,-1.3805599651,0.5989952102
H,-2.0185266855,-2.9639084015,1.6515945555

N,2.6724201941,4.623285882,0.3473635477
O,3.7442487121,4.997655478,-0.0932734627
O,1.8491245471,5.3152374463,0.9229578726

3dH⁺·H₂O

Electronic Energy= -775.900940823 Hartree
NIMAG= 0
C,0.2700651629,1.3037824606,-0.009466572
C,1.1152250611,0.286824424,-0.5313256525
C,2.4895760767,0.5401159998,-0.7355183671
C,2.9714761769,1.7859768644,-0.4092618424
C,2.0961818512,2.7655026531,0.1122994005
C,0.7506746158,2.5750422568,0.3278270915
H,4.0135903485,2.0423900165,-0.5408443038
H,0.1347691807,3.3693864459,0.7262253273
N,-0.9904864557,0.7906432914,0.0642373302
N,-0.9551398326,-0.5011012245,-0.352291142
C,0.2804358355,-0.8319376394,-0.7219736246
H,0.4972483114,-1.8202099198,-1.0972636834
C,-2.2044019565,-1.3329954739,-0.3611837041
H,-2.8982163179,-0.8449141939,-1.0515607488
H,-1.8964791857,-2.2975183671,-0.7624806676
O,-2.7084783463,-1.5022681305,0.9074676961
H,-3.2947441692,-0.7648206124,1.1356203007
H,3.1450872463,-0.2230368721,-1.135076645
H,-1.8331953264,1.1522130626,0.5256791495
O,-3.5089195385,1.2793423416,1.3673201288
H,-3.5396640415,1.4659326922,2.3155454047
H,-4.2497807779,1.7683232336,0.9850014902
N,2.6642284856,4.1072972753,0.4550330448
O,3.8534480668,4.2704855439,0.2518134882
O,1.8910662586,4.9332919918,0.911819811

TS (2N: 1dH⁺·CH₂O·H₂O→ 3dH⁺·H₂O)

Electronic Energy= -775.856669224 Hartree
NIMAG= 1
C,0.3257322101,1.9604282893,0.8649327176
C,1.0231886394,0.9686880754,0.1320976426
C,2.2752324999,1.2675774425,-0.4438792847
C,2.7879145932,2.5341478359,-0.2693806717
C,2.0590631638,3.4898924427,0.4706658925
C,0.8327305875,3.2508385933,1.053060929
H,3.7431174617,2.821333327,-0.6860236698
H,0.3242515994,4.0271663239,1.6082853475
N,-0.8383320524,1.3779066438,1.2841077539
N,-0.9239611089,0.0794178776,0.8874619051
C,0.1804884877,-0.171304687,0.1863126653
H,0.3387527027,-1.1483894852,-0.247216586

C,-2.9574985098,-1.0518230917,-0.467970964
H,-3.3845465687,-0.3785724614,0.2720367344
H,-2.8814487564,-0.7535299068,-1.5136717767
O,-2.6203660294,-2.2083724528,-0.1697630816
H,-2.5875648401,-2.3487943939,0.899225126
H,2.8235666584,0.5233290863,-1.0077223778
H,-1.5462230367,1.7698874241,1.8856384683
O,-2.3682466281,-2.1762765287,2.2534115706
H,-1.6492560145,-1.5299266654,2.3601440433
H,-2.2604262778,-2.9003895932,2.8835843041
N,2.6481199965,4.8510362184,0.63741175
O,3.7341456737,5.0535530937,0.1217081567
O,1.9993696789,5.663561002,1.2782405855

2N: 1eH$^+$·CH_2O·H_2O
Electronic Energy= -775.902771048 Hartree
NIMAG= 0
C,0.8515973271,1.3726557194,0.4652420412
C,1.7783236285,0.527350486,-0.2016952749
C,2.9977446721,1.0498327168,-0.6846351828
C,3.2575140738,2.3902508598,-0.4910071625
C,2.3338533854,3.2301893183,0.1726978028
C,1.1387047747,2.7362404319,0.6503608398
H,4.1840906332,2.8189866751,-0.8501512581
H,2.5504920293,4.2814939469,0.3179203578
N,-0.2119611216,0.6145547592,0.8142207601
N,-0.0007773488,-0.6560813551,0.4013033594
C,1.1733243788,-0.748795346,-0.2082264733
H,1.5141417265,-1.6914331976,-0.6074293402
C,-4.8588792535,-3.1774364751,-1.3649257382
H,-5.3862591765,-3.8953509967,-0.7142731747
H,-5.3233913677,-2.9445755809,-2.3363596885
O,-3.8144108145,-2.6621994985,-1.0350708453
H,-2.6386832732,-2.5772903668,0.2722445037
H,3.7093776156,0.4118457138,-1.1938900548
H,-1.0499938453,0.9135750232,1.3000264861
O,-1.9055692959,-2.3916213083,0.8985200742
H,-0.7370984307,-1.4031403466,0.5876626103
H,-1.9605893251,-3.0219882548,1.6238484394
N,0.174753136,3.597635615,1.3384412665
O,0.4453204507,4.7706597099,1.4862562472
O,-0.8619472991,3.0461483911,1.7219751346

3eH$^+$·H_2O
Electronic Energy= -775.908988837 Hartree
NIMAG= 0
C,0.254264525,1.3153700965,-0.002694368
C,1.1011982102,0.3039234885,-0.5332340894

C,2.4743927602,0.5549820918,-0.7444028586
C,2.9679917313,1.8008004631,-0.4206355199
C,2.1295380632,2.8047883681,0.1145901879
C,0.786347828,2.5753204795,0.3280689382
H,4.0162842128,2.0240512596,-0.5706416
H,2.5310419847,3.7773289302,0.3720748369
N,-0.9956731007,0.8057928317,0.0778826566
N,-0.9682262361,-0.4819019041,-0.3529758778
C,0.2649015796,-0.8142961479,-0.7252196356
H,0.48327136,-1.8028434815,-1.0986631491
C,-2.2185850377,-1.319719414,-0.3199886771
H,-2.9408018666,-0.8165072502,-0.9688944218
H,-1.9255464454,-2.2735647889,-0.7569175513
O,-2.6621194378,-1.5149612592,0.9637851055
H,-3.2287545497,-0.7733093129,1.2342110472
H,3.1209040528,-0.2140172259,-1.1487251956
H,-1.8483302572,1.2167723231,0.4792977141
O,-3.583372792,1.1843186763,1.2753091545
H,-3.5093706448,1.6130696968,2.1389275415
H,-4.3447788831,1.6016404429,0.8517089069
N,-0.0759309033,3.6089066903,0.902631892
O,0.4015323632,4.6957651561,1.1501304245
O,-1.2512482566,3.2804973402,1.0978332984

TS (2N: 1eH^{+}·CH_2O·H_2O→ 3eH^{+}·H_2O)
Electronic Energy= -775.864630293 Hartree
NIMAG= 1
C,0.3364525827,1.9199417306,0.8648357822
C,1.0685904774,0.9581310135,0.1258633624
C,2.3227209737,1.2905575386,-0.4227721952
C,2.8150532267,2.5666286746,-0.2206711429
C,2.08724661,3.5232347682,0.5172370476
C,0.8560273957,3.2112699787,1.0608379107
H,3.7766090359,2.8469971189,-0.6309265788
H,2.4815737809,4.5198443811,0.6710259503
N,-0.8126524483,1.3182440015,1.2578410004
N,-0.872982793,0.0237878056,0.8366518081
C,0.244466823,-0.1985267569,0.1492222031
H,0.427167182,-1.1652217822,-0.2975169219
C,-3.0062608337,-1.0114609612,-0.4376890094
H,-3.3672490997,-0.3089563861,0.3104412126
H,-2.9501344908,-0.7244524197,-1.4878536624
O,-2.7285201606,-2.1838738581,-0.1419128627
H,-2.6620390143,-2.3145157649,0.9282243318
H,2.8904337957,0.5621108856,-0.9890625794
H,-1.5224309702,1.7414541223,1.83961489
O,-2.3791890169,-2.1265699937,2.2652045308
H,-1.6250262151,-1.5142430221,2.3243278837
H,-2.2861287567,-2.8361001838,2.9137479555

N,0.0958864274,4.1957142852,1.828390395
O,0.5618227803,5.3076226204,1.9789292383
O,-0.9912979622,3.8135803937,2.2773415602

GIAO calculations (ppm)

The equations to transform the absolute chemical shieldings into chemical shifts are in the following references:

^{1}H: A. M. S. Silva, et al. Magn. Reson. Chem. 2008, 46, 859-864
^{13}C: F. Blanco, I. Alkorta, J. Elguero Magn. Reson. Chem. 2007, 45, 797-800
^{15}N: F. Blanco, I. Alkorta, J. Elguero Magn. Reson. Chem. 2007, 45, 797-800
^{17}O: D. Sanz, et al. Magn. Reson. Chem. 2012, 50, 246-255

2a
****

Atom	Abs.	Rel.
1C	37.40	139.69
2C	50.93	126.65
3C	57.27	120.55
4C	56.99	120.82
5C	51.58	126.03
6C	69.92	108.37
7H	24.58	7.16
8H	24.36	7.37
9H	24.25	7.48
10N	30.76	-181.10
11N	-96.55	-60.67
12C	43.25	134.05
13H	23.89	7.83
14C	107.28	72.39
15H	26.02	5.76
16H	26.26	5.53
17O	252.52	23.23
18H	30.32	1.59
19H	24.07	7.65

2b_4nitroindazol_ch2oh_giao
****

Atom	Abs.	Rel.
1C	36.35	140.69
2C	57.57	120.26
3C	34.85	142.14
4C	58.58	119.29
5C	53.36	124.32
6C	62.29	115.71
7H	23.44	8.26
8H	24.38	7.35
9H	23.95	7.77
10N	27.25	-177.78
11N	-107.74	-50.07
12C	41.86	135.38
13H	22.89	8.79
14C	106.64	73.01
15H	25.96	5.82

16H	26.22	5.57
17O	253.31	22.53
18H	30.16	1.74
19N	-144.00	-15.78
20O	-330.51	546.80

2c

****

Atom	Abs.	Rel.
1C	35.81	141.21
2C	52.13	125.50
3C	59.24	118.65
4C	32.39	144.51
5C	55.24	122.51
6C	70.51	107.80
7H	23.29	8.40
8H	24.33	7.40
9N	26.62	-177.18
10N	-105.29	-52.40
11C	40.53	136.67
12H	23.72	8.00
13C	106.83	72.82
14H	26.03	5.75
15H	26.28	5.51
16O	253.71	22.17
17H	30.11	1.79
18H	22.99	8.70
19N	-142.35	-17.34
20O	-310.08	528.45

2d

****

Atom	Abs.	Rel.
1C	39.11	138.04
2C	48.13	129.35
3C	57.71	120.12
4C	60.93	117.02
5C	29.02	147.75
6C	72.10	106.27
7H	24.08	7.64
8H	23.55	8.16
9H	23.14	8.55
10N	23.98	-174.69
11N	-112.72	-45.37
12C	43.18	134.12
13H	23.77	7.94
14C	106.92	72.73
15H	26.15	5.63
16H	25.97	5.81
17O	252.38	23.36

18H	30.11	1.80
19N	-142.97	-16.75
20O	-303.31	522.37
21O	-317.34	534.97

2e

****

Atom	Abs.	Rel.
1C	46.27	131.14
2C	46.21	131.20
3C	50.77	126.81
4C	58.34	119.52
5C	51.86	125.76
6C	38.33	138.79
7H	24.59	7.15
8H	23.53	8.17
9N	21.51	-172.35
10N	-109.51	-48.40
11C	42.58	134.70
12H	23.75	7.96
13C	103.05	76.47
14H	25.94	5.84
15H	25.26	6.49
16O	255.21	20.82
17H	30.50	1.42
18H	23.84	7.87
19N	-145.80	-14.08
20O	-337.81	553.35
21O	-339.83	555.17

3a

****

Atom	Abs.	Rel.
1C	25.85	150.81
2C	54.02	123.68
3C	57.67	120.17
4C	55.43	122.32
5C	51.54	126.07
6C	58.90	118.98
7H	24.68	7.06
8H	24.47	7.26
9H	24.00	7.72
10N	-62.09	-93.27
11N	-7.94	-144.49
12C	57.03	120.78
13H	23.90	7.82
14C	102.83	76.68
15H	26.64	5.16
16H	25.99	5.79
17O	249.75	25.72
18H	29.74	2.15

19H	24.10	7.62

3b

****

Atom	Abs.	Rel.
1C	25.49	151.16
2C	61.28	116.68
3C	34.35	142.63
4C	55.85	121.91
5C	53.81	123.88
6C	49.53	128.00
7H	23.45	8.25
8H	24.44	7.29
9H	23.68	8.03
10N	-64.11	-91.35
11N	-16.86	-136.05
12C	52.38	125.26
13H	22.99	8.70
14C	102.52	76.98
15H	26.50	5.30
16H	25.99	5.79
17O	250.99	24.62
18H	29.59	2.29
19N	-143.61	-16.14
20O	-324.04	540.99
21O	-302.70	521.83

3c

****

Atom	Abs.	Rel.
1C	25.31	151.32
2C	56.18	121.60
3C	57.86	119.98
4C	31.54	145.32
5C	56.03	121.74
6C	59.29	118.61
7H	23.47	8.23
8H	24.08	7.64
9N	-64.11	-91.35
10N	-16.68	-136.22
11C	51.85	125.77
12H	23.66	8.05
13C	102.50	76.99
14H	26.61	5.19
15H	26.01	5.77
16O	250.70	24.87
17H	29.55	2.33
18H	22.91	8.78
19N	-142.98	-16.74
20O	-314.32	532.26
21O	-295.53	515.39

3d

****

Atom	Abs.	Rel.
1C	28.04	148.69
2C	51.83	125.79
3C	57.58	120.25
4C	60.38	117.55
5C	28.36	148.39
6C	60.34	117.59
7H	24.12	7.61
8H	23.66	8.05
9H	22.86	8.82
10N	-74.11	-81.90
11N	-19.52	-133.54
12C	55.76	122.00
13H	23.83	7.88
14C	102.26	77.22
15H	25.97	5.81
16H	26.57	5.23
17O	250.22	25.31
18H	29.61	2.28
19N	-143.53	-16.22
20O	-302.25	521.42
21O	-317.00	534.67

3e

****

Atom	Abs.	Rel.
1C	34.34	142.63
2C	50.93	126.66
3C	48.73	128.77
4C	57.88	119.96
5C	51.20	126.40
6C	37.28	139.80
7H	24.66	7.08
8H	23.20	8.49
9N	-67.98	-87.69
10N	-16.61	-136.28
11C	54.82	122.91
12H	23.74	7.97
13C	102.33	77.15
14H	26.62	5.18
15H	25.89	5.89
16O	251.29	24.34
17H	29.29	2.59
18H	23.79	7.92
19N	-142.08	-17.59
20O	-360.47	573.70
21O	-300.26	519.64

Coupling Constants (Hz)

2a

Atom	1	2	3	4	5	6	7	8	9	10	11	12	13	14	15	16	17	18
1	0.00																	
2	58.83	0.00																
3	1.72	67.59	0.00															
4	8.50	-1.27	65.47	0.00														
5	0.44	7.13	-1.45	61.50	0.00													
6	70.27	1.76	6.10	-1.96	66.54	0.00												
7	-1.06	7.71	1.42	150.06	2.39	7.21	0.00											
8	9.06	-1.05	7.39	1.84	149.12	1.59	6.45	0.00										
9	-0.02	4.28	-1.31	6.49	0.16	152.32	0.30	7.74	0.00									
10	10.27	4.55	1.11	-0.19	1.27	0.71	0.10	0.48	0.36	0.00								
11	-1.12	-1.48	0.88	-0.31	0.66	-0.41	0.01	-0.14	0.14	-7.05	0.00							
12	3.67	57.69	2.67	4.56	-1.33	3.21	0.77	0.44	-0.12	0.14	0.21	0.00						
13	3.07	11.29	0.54	-0.45	0.40	-0.15	-0.08	-0.35	0.65	5.53	9.23	179.35	0.00					
14	3.53	1.22	0.01	0.11	-0.09	0.59	-0.02	-0.03	0.04	9.18	5.10	4.19	0.13	0.00				
15	4.54	0.23	0.24	-0.03	-0.08	0.01	-0.16	-0.16	-0.17	-0.16	1.28	-0.20	-0.20	150.64	0.00			
16	0.88	-0.28	-0.07	-0.04	0.00	0.29	-0.15	-0.03	0.29	1.10	2.71	0.69	0.17	147.12	-10.67	0.00		
17	0.30	0.15	0.04	-0.02	0.00	0.04	0.05	0.02	0.01	-0.74	0.03	-0.32	0.33	23.46	-1.94	-15.01	0.00	
18	-0.09	-0.09	-0.03	-0.06	-0.03	-0.04	-0.13	-0.15	-0.18	0.14	0.05	0.04	-0.08	-3.93	2.68	13.34	-65.60	0.00
19	6.53	2.41	150.68	0.78	7.04	-1.44	7.39	0.45	0.70	0.01	-0.04	2.89	-0.04	0.32	-0.06	-0.18	0.01	-0.12

2b

Atom	1	2	3	4	5	6	7	8	9	10	11	12	13	14	15	16	17	18	19	20
**1**	0.00																			
**2**	59.30	0.00																		
**3**	4.63	81.13	0.00																	
**4**	6.40	1.62	76.03	0.00																
**5**	1.00	4.58	0.79	62.02	0.00															
**6**	70.29	2.06	5.40	-2.21	65.59	0.00														
**7**	-0.71	4.89	-4.00	158.53	1.14	7.62	0.00													
**8**	9.30	-1.08	8.69	2.55	154.20	1.78	7.33	0.00												
**9**	0.19	4.44	-1.70	6.84	0.33	153.95	0.24	7.62	0.00											
**10**	10.36	5.06	1.44	-0.28	1.40	0.69	0.08	0.46	0.41	0.00										
**11**	-1.20	-1.58	0.92	-0.11	0.55	-0.25	-0.02	-0.13	0.13	-7.19	0.00									
**12**	3.13	59.58	-0.07	2.30	-1.04	2.99	1.33	0.27	-0.28	-0.34	-0.42	0.00								
**13**	2.84	10.03	0.24	-0.32	0.33	-0.15	-0.16	-0.35	0.64	5.77	9.93	194.05	0.00							
**14**	3.60	1.05	0.10	0.06	-0.11	0.55	0.00	-0.02	0.03	8.89	5.12	4.13	0.18	0.00						
**15**	4.75	0.30	0.35	-0.06	-0.07	0.01	-0.14	-0.16	-0.16	-0.12	1.27	-0.24	-0.16	151.73	0.00					
**16**	0.78	-0.27	-0.08	-0.04	0.03	0.29	-0.12	-0.04	0.32	1.17	2.61	0.60	0.17	148.08	-10.88	0.00				
**17**	0.26	0.16	0.03	0.00	-0.02	0.04	0.03	0.03	-0.02	-0.72	0.06	-0.28	0.32	23.55	-1.69	-15.20	0.00			
**18**	-0.08	-0.09	-0.02	-0.05	-0.03	-0.04	-0.13	-0.14	-0.18	0.00	0.04	0.04	-0.08	-3.94	3.28	13.52	-66.06	0.00		
**19**	1.62	1.54	9.93	1.20	1.55	-0.44	1.36	0.36	0.27	0.01	0.04	-0.17	0.09	0.05	0.04	-0.01	0.00	-0.01	0.00	
**20**	0.18	-0.10	-1.86	-2.07	-0.06	-0.39	0.72	-0.10	0.09	0.02	-0.17	-3.26	-1.24	-0.02	0.01	0.00	-0.01	0.00	31.16	0.00
**21**	0.06	-0.75	-0.95	-2.02	0.26	-0.84	-0.44	-0.04	0.13	-0.19	-0.08	0.15	0.23	0.03	0.03	0.03	0.02	0.03	31.32	-8.26

2c

Atom	1	2	3	4	5	6	7	8	9	10	11	12	13	14	15	16	17	18	19	20
**1**	0.00																			
**2**	58.45	0.00																		
**3**	1.82	68.93	0.00																	
**4**	7.31	1.77	76.69	0.00																
**5**	0.10	4.35	1.87	73.47	0.00															
**6**	69.86	3.00	3.75	0.89	67.94	0.00														
**7**	9.32	-0.62	4.33	-3.10	159.00	0.68	0.00													
**8**	0.30	4.66	-1.26	7.93	0.90	156.18	8.60	0.00												
**9**	10.43	4.84	1.11	-0.17	1.43	0.83	0.39	0.30	0.00											
**10**	-1.25	-1.46	0.96	-0.34	0.61	-0.38	-0.14	0.14	-7.25	0.00										
**11**	4.01	57.91	3.47	5.18	-1.21	3.31	0.28	-0.07	-0.21	0.01	0.00									
**12**	2.97	11.53	0.47	-0.53	0.39	-0.16	-0.32	0.65	5.63	9.28	183.03	0.00								
**13**	3.43	1.16	0.00	0.10	-0.08	0.61	-0.01	0.04	8.70	5.04	4.02	0.10	0.00							
**14**	4.61	0.24	0.21	-0.02	-0.07	0.01	-0.15	-0.16	-0.15	1.26	-0.22	-0.17	151.61	0.00						
**15**	0.86	-0.27	-0.09	-0.01	0.02	0.29	-0.02	0.31	1.23	2.69	0.68	0.19	148.24	-10.86	0.00					
**16**	0.29	0.14	0.04	-0.02	-0.02	0.05	0.02	-0.01	-0.73	0.04	-0.27	0.30	23.56	-1.70	-14.96	0.00				
**17**	-0.09	-0.10	-0.03	-0.04	-0.02	-0.04	-0.13	-0.18	-0.01	0.04	0.03	-0.10	-3.95	3.35	13.52	-66.06	0.00			
**18**	6.69	1.33	159.51	-4.24	3.94	-1.02	1.47	0.51	0.01	-0.04	3.30	-0.02	0.32	-0.02	-0.15	0.01	-0.11	0.00		
**19**	-0.32	1.76	1.33	9.75	1.37	1.62	0.96	0.32	0.03	0.00	0.10	0.00	0.00	-0.01	-0.01	0.01	0.00	1.27	0.00	
**20**	-0.50	-0.37	-2.06	-1.05	-1.66	0.13	-0.43	-0.05	-0.02	0.06	0.14	0.10	0.01	0.03	0.01	0.00	0.02	0.73	31.21	0.00
**21**	-0.40	0.09	-1.92	-0.89	-1.20	-0.35	0.72	-0.08	-0.12	0.07	-0.11	0.03	0.01	0.01	0.02	0.00	0.02	-0.42	31.25	-8.38

2d

Atom	1	2	3	4	5	6	7	8	9	10	11	12	13	14	15	16	17	18	19	20
**1**	0.00																			
**2**	58.62	0.00																		
**3**	3.07	67.35	0.00																	
**4**	5.49	-1.58	66.44	0.00																
**5**	4.21	6.24	1.36	73.20	0.00															
**6**	72.49	1.92	3.89	1.28	78.96	0.00														
**7**	7.13	2.64	154.51	1.64	8.65	-1.39	0.00													
**8**	-0.57	7.98	0.42	160.14	-2.86	4.14	8.24	0.00												
**9**	-1.06	4.39	-0.81	3.57	-4.77	160.49	0.54	1.33	0.00											
**10**	10.55	4.66	1.15	-0.08	1.46	1.00	0.03	-0.01	0.51	0.00										
**11**	-1.16	-1.47	0.94	-0.37	0.62	-0.28	-0.03	0.02	0.15	-7.17	0.00									
**12**	4.17	57.80	2.98	4.77	-1.51	3.32	2.75	0.59	-0.19	-0.13	-0.15	0.00								
**13**	3.07	11.37	0.54	-0.44	0.45	-0.18	-0.02	-0.04	0.75	5.41	9.18	182.41	0.00							
**14**	3.46	1.04	0.00	0.09	-0.11	0.59	0.31	-0.01	0.03	8.64	4.95	4.04	0.09	0.00						
**15**	0.88	-0.25	-0.07	-0.04	0.04	0.28	-0.17	-0.15	0.36	1.23	2.76	0.70	0.17	148.47	0.00					
**16**	4.71	0.28	0.25	-0.03	-0.05	-0.01	-0.04	-0.17	-0.11	-0.07	1.23	-0.23	-0.20	151.55	-10.87	0.00				
**17**	0.28	0.16	0.04	-0.02	-0.01	0.05	0.02	0.05	-0.01	-0.73	0.04	-0.28	0.32	23.69	-14.85	-1.69	0.00			
**18**	-0.09	-0.10	-0.03	-0.05	-0.01	-0.03	-0.12	-0.12	-0.16	-0.01	0.04	0.03	-0.09	-3.98	13.49	3.37	-65.96	0.00		
**19**	2.08	-0.32	1.69	1.27	9.23	1.43	0.39	0.99	1.38	0.05	-0.08	0.14	-0.04	0.00	-0.01	-0.01	0.00	-0.01	0.00	
**20**	0.15	-0.35	-0.14	-1.19	-1.06	-2.07	-0.08	0.70	-0.42	-0.06	-0.09	0.06	0.01	0.01	-0.01	0.01	0.00	0.01	31.36	0.00
**21**	-0.20	-0.53	0.13	-1.74	-1.34	-1.87	-0.04	-0.47	0.73	0.12	-0.03	0.17	0.03	0.02	0.07	0.05	-0.01	0.04	31.28	-8.06

2e

Atom	1	2	3	4	5	6	7	8	9	10	11	12	13	14	15	16	17	18	19	20
**1**	0.00																			
**2**	59.05	0.00																		
**3**	2.03	67.62	0.00																	
**4**	5.77	-1.11	63.46	0.00																
**5**	3.10	6.01	-1.64	62.56	0.00															
**6**	83.43	3.59	5.72	-0.20	76.21	0.00														
**7**	-1.12	7.56	1.43	155.12	3.10	8.43	0.00													
**8**	6.40	-0.76	7.99	0.55	156.72	-3.43	7.27	0.00												
**9**	10.61	4.30	0.86	-0.05	0.52	0.23	0.03	0.63	0.00											
**10**	-1.37	-1.45	0.96	-0.35	0.70	-0.46	0.02	-0.16	-7.42	0.00										
**11**	4.68	56.78	2.84	4.79	-1.30	3.96	0.67	0.34	-0.40	0.10	0.00									
**12**	2.89	12.00	0.62	-0.44	0.31	0.04	-0.07	-0.27	5.52	9.16	182.18	0.00								
**13**	2.50	0.79	-0.06	0.04	-0.04	-0.19	-0.05	-0.05	7.11	4.96	3.83	-0.04	0.00							
**14**	4.74	0.15	0.25	-0.07	0.00	-0.26	-0.15	-0.19	0.20	1.66	-0.12	-0.16	149.90	0.00						
**15**	0.08	-0.23	-0.17	-0.01	-0.06	-0.11	-0.20	-0.12	1.19	2.88	0.84	0.15	157.84	-10.26	0.00					
**16**	0.18	0.11	0.04	-0.03	0.01	-0.05	0.04	0.01	-0.60	0.00	-0.23	0.25	24.23	-1.32	-14.99	0.00				
**17**	-0.06	-0.07	-0.02	-0.04	-0.02	-0.02	-0.12	-0.16	-0.14	0.08	0.07	-0.09	-4.23	4.07	13.51	-64.32	0.00			
**18**	6.66	2.80	152.29	0.83	7.16	-1.75	7.21	0.48	-0.03	-0.05	3.25	-0.10	0.30	-0.06	-0.16	0.00	-0.11	0.00		
**19**	0.72	0.98	-0.39	1.43	1.14	10.73	0.28	1.60	-0.07	0.04	0.09	0.28	0.21	0.05	-0.01	-0.04	0.02	0.26	0.00	
**20**	-0.61	0.06	-0.56	0.14	-1.35	-0.79	-0.04	-0.38	-0.05	0.07	-0.15	0.05	-0.25	0.00	-0.12	0.05	-0.05	0.12	31.37	0.00
**21**	0.27	0.16	-0.30	-0.05	-0.90	-1.53	-0.11	0.48	0.01	-0.03	0.01	0.05	-4.98	-0.51	1.40	-0.03	-0.24	0.06	31.11	-8.47

3a

Atom	1	2	3	4	5	6	7	8	9	10	11	12	13	14	15	16	17	18
**1**	0.0																	
**2**	47.3	0.0																
**3**	0.4	66.8	0.0															
**4**	8.2	-1.0	68.9	0.0														
**5**	0.1	7.1	-1.8	59.2	0.0													
**6**	70.8	2.7	6.1	-2.3	68.6	0.0												
**7**	-1.0	8.5	1.2	149.2	2.3	6.6	0.0											
**8**	9.0	-1.0	7.1	2.0	148.4	0.8	6.2	0.0										
**9**	0.3	4.5	-1.5	7.3	0.6	153.3	0.3	8.1	0.0									
**10**	0.3	-1.6	0.2	-0.6	2.5	5.3	0.1	0.4	0.3	0.0								
**11**	0.8	3.6	2.2	0.0	0.4	2.9	0.0	0.0	-0.1	-7.1	0.0							
**12**	3.1	75.5	4.4	5.9	-1.5	2.1	0.9	0.3	-0.2	-0.7	9.5	0.0						
**13**	6.6	8.0	1.1	-0.7	0.5	-0.3	0.1	-0.5	0.8	-0.6	3.2	179.9	0.0					
**14**	4.6	1.8	0.0	0.2	0.1	0.0	0.1	0.1	0.0	5.0	8.3	4.8	1.3	0.0				
**15**	0.8	-0.2	0.0	-0.1	0.0	0.2	-0.1	-0.1	-0.2	2.7	0.9	1.9	0.3	147.0	0.0			
**16**	-0.2	0.5	0.2	-0.1	0.0	-0.1	-0.1	-0.2	0.0	1.0	0.0	4.0	-0.4	152.4	-9.3	0.0		
**17**	-0.2	0.1	0.0	0.0	-0.1	0.1	0.0	0.1	0.0	-0.1	-0.5	0.2	0.2	23.4	-13.4	-2.2	0.0	
**18**	0.1	-0.1	0.0	0.0	0.0	0.0	-0.1	-0.1	0.0	0.0	0.2	-0.1	-0.2	-4.2	13.4	2.6	-66.6	0.0
**19**	5.5	2.4	150.9	0.4	7.5	-1.6	7.8	0.5	0.9	-0.3	0.0	2.4	-0.2	0.0	-0.1	-0.2	0.0	-0.1

3b

Atom	1	2	3	4	5	6	7	8	9	10	11	12	13	14	15	16	17	18	19	20
**1**	0.0																			
**2**	47.5	0.0																		
**3**	2.4	80.4	0.0																	
**4**	6.4	2.2	78.6	0.0																
**5**	0.6	4.7	0.5	60.1	0.0															
**6**	71.2	3.1	5.2	-2.4	67.4	0.0														
**7**	-0.7	5.6	-4.3	157.3	0.9	6.9	0.0													
**8**	9.2	-1.0	8.4	2.7	153.7	1.1	7.2	0.0												
**9**	0.4	4.7	-1.9	7.6	0.9	154.8	0.2	7.9	0.0											
**10**	0.2	-1.5	0.3	-0.7	2.4	5.4	0.1	0.4	0.4	0.0										
**11**	0.8	3.9	2.1	0.2	0.3	3.0	-0.1	0.1	-0.1	-7.1	0.0									
**12**	2.6	79.6	1.2	3.3	-0.9	1.8	1.5	0.2	-0.3	-1.0	8.6	0.0								
**13**	6.4	6.7	0.8	-0.6	0.4	-0.3	0.0	-0.4	0.7	-0.5	3.7	193.1	0.0							
**14**	4.6	1.9	0.0	0.1	0.1	0.0	0.1	0.1	0.0	4.7	7.7	5.2	1.4	0.0						
**15**	0.8	-0.1	0.0	-0.1	0.0	0.2	-0.1	-0.1	-0.2	2.7	1.0	1.9	0.3	149.0	0.0					
**16**	-0.3	0.5	0.2	0.0	-0.1	-0.1	-0.1	-0.2	0.0	0.9	0.1	3.9	-0.4	152.8	-9.4	0.0				
**17**	-0.2	0.1	0.0	0.0	-0.1	0.1	0.0	0.1	0.0	0.0	-0.5	0.2	0.2	23.6	-13.1	-2.0	0.0			
**18**	0.1	-0.1	0.0	-0.1	0.0	0.0	-0.1	-0.1	0.1	0.0	0.1	-0.1	-0.2	-4.3	13.6	3.1	-66.8	0.0		
**19**	1.3	1.2	9.9	1.1	1.6	-0.5	1.5	0.4	0.3	0.0	0.1	-0.3	0.0	0.0	0.0	0.0	0.0	0.0	0.0	
**20**	0.1	0.0	-1.8	-2.3	-0.1	-0.4	0.7	-0.1	0.1	0.1	-0.1	-3.2	-1.1	0.0	0.0	0.0	0.0	0.0	31.0	0.0
**21**	0.2	-0.5	-0.4	-2.3	0.4	-1.0	-0.4	0.0	0.2	-0.5	0.0	0.2	0.2	0.0	0.1	0.1	0.0	0.0	31.3	-8.4

3c

Atom	1	2	3	4	5	6	7	8	9	10	11	12	13	14	15	16	17	18	19	20
**1**	0.0																			
**2**	47.1	0.0																		
**3**	0.6	68.4	0.0																	
**4**	7.3	2.4	79.7	0.0																
**5**	-0.3	4.3	1.6	70.8	0.0															
**6**	70.6	4.1	3.9	0.3	69.7	0.0														
**7**	9.4	-0.6	4.1	-2.8	159.0	0.1	0.0													
**8**	0.6	4.9	-1.4	8.9	1.6	157.0	8.9	0.0												
**9**	0.2	-1.5	0.2	-0.5	2.5	5.6	0.4	0.3	0.0											
**10**	0.6	3.6	2.3	0.0	0.3	2.8	0.1	-0.1	-7.3	0.0										
**11**	3.6	75.5	5.4	6.7	-1.4	2.1	0.2	-0.1	-1.0	9.5	0.0									
**12**	6.4	8.0	1.1	-0.8	0.5	-0.3	-0.4	0.7	-0.6	3.1	182.9	0.0								
**13**	4.3	1.8	0.0	0.2	0.0	0.0	0.1	0.0	4.8	7.7	4.7	1.4	0.0							
**14**	0.8	-0.2	0.0	-0.1	0.0	0.3	-0.1	-0.2	2.7	1.1	1.9	0.3	148.4	0.0						
**15**	-0.3	0.5	0.3	0.0	0.0	-0.1	-0.1	0.0	1.0	0.0	4.0	-0.4	153.4	-9.5	0.0					
**16**	-0.2	0.1	-0.1	0.0	-0.1	0.1	0.1	0.0	-0.1	-0.5	0.2	0.2	23.6	-13.3	-2.0	0.0				
**17**	0.1	-0.1	0.0	0.0	0.0	0.0	-0.1	0.0	0.0	0.1	-0.1	-0.2	-4.2	13.6	3.2	-67.0	0.0			
**18**	5.6	1.1	159.5	-4.7	4.2	-1.3	1.5	0.6	-0.2	0.0	2.9	-0.2	0.1	0.0	-0.2	0.0	-0.1	0.0		
**19**	-0.3	2.0	1.3	9.5	1.3	1.5	0.9	0.3	0.0	0.0	0.1	0.0	0.0	0.0	0.0	0.0	0.0	1.4	0.0	
**20**	-0.4	-0.4	-2.4	-1.1	-1.7	0.1	-0.4	-0.1	0.1	0.0	0.0	0.1	0.0	0.0	0.0	0.0	0.0	0.8	31.2	0.0
**21**	-0.3	0.1	-2.1	-0.7	-0.9	-0.3	0.7	-0.1	-0.1	0.0	-0.3	0.1	0.0	0.0	0.0	0.0	0.0	-0.4	31.3	-8.4

3d

Atom	1	2	3	4	5	6	7	8	9	10	11	12	13	14	15	16	17	18	19	20
**1**	0.0																			
**2**	47.6	0.0																		
**3**	1.6	66.8	0.0																	
**4**	5.3	-1.3	69.9	0.0																
**5**	3.8	6.2	1.1	70.9	0.0															
**6**	72.7	2.8	4.0	0.7	80.0	0.0														
**7**	6.1	2.6	154.5	1.4	9.2	-1.5	0.0													
**8**	-0.4	8.8	0.3	159.8	-2.7	3.8	8.6	0.0												
**9**	-0.9	4.6	-1.0	4.1	-4.7	161.7	0.7	1.4	0.0											
**10**	0.2	-1.5	0.2	-0.5	2.9	5.8	-0.2	0.0	0.5	0.0										
**11**	0.9	3.3	2.1	-0.1	0.5	3.0	0.0	0.1	-0.1	-7.2	0.0									
**12**	3.7	75.7	4.8	6.0	-1.7	2.1	2.3	0.8	-0.2	-0.9	9.0	0.0								
**13**	6.7	8.1	1.1	-0.7	0.6	-0.3	-0.2	0.1	0.8	-0.6	3.3	182.4	0.0							
**14**	4.6	1.7	0.0	0.2	0.1	0.0	0.0	0.1	0.0	5.0	7.7	4.7	1.3	0.0						
**15**	-0.3	0.5	0.2	-0.1	0.0	-0.1	-0.2	-0.1	0.0	1.0	0.0	4.0	-0.3	153.2	0.0					
**16**	0.8	-0.2	0.0	-0.1	0.0	0.3	-0.1	-0.1	-0.1	2.7	1.1	1.8	0.3	148.5	-9.5	0.0				
**17**	-0.2	0.1	-0.1	0.0	-0.1	0.1	0.0	0.0	0.0	0.0	-0.5	0.2	0.2	23.6	-1.9	-13.4	0.0			
**18**	0.1	-0.1	0.0	0.0	0.0	0.0	-0.1	-0.1	0.1	0.0	0.0	-0.1	-0.2	-4.2	3.3	13.6	-66.9	0.0		
**19**	2.2	-0.3	1.6	1.2	9.2	1.3	0.4	0.9	1.5	0.1	0.0	0.1	-0.1	0.0	0.0	0.0	0.0	0.0	0.0	
**20**	0.2	-0.3	-0.2	-0.9	-0.9	-2.2	-0.1	0.7	-0.4	-0.2	0.0	0.1	0.0	0.0	0.0	0.0	0.0	0.0	31.3	0.0
**21**	-0.2	-0.4	0.1	-1.7	-1.3	-2.4	-0.1	-0.5	0.7	0.0	-0.1	0.2	0.0	0.0	0.0	0.0	0.0	0.0	31.2	-8.2

3e

Atom	1	2	3	4	5	6	7	8	9	10	11	12	13	14	15	16	17	18	19	20
**1**	0.0																			
**2**	47.6	0.0																		
**3**	0.7	67.0	0.0																	
**4**	5.7	-0.6	66.6	0.0																
**5**	2.9	5.9	-1.9	60.2	0.0															
**6**	83.4	4.7	5.4	-0.3	78.3	0.0														
**7**	-1.0	8.4	1.4	154.4	2.9	7.8	0.0													
**8**	6.2	-0.7	7.7	0.8	156.1	-4.7	7.2	0.0												
**9**	0.0	-1.4	0.0	-0.4	1.2	3.3	0.0	0.7	0.0											
**10**	0.9	3.6	2.3	-0.1	0.5	2.6	0.0	0.0	-7.6	0.0										
**11**	4.1	74.8	4.7	6.3	-1.5	2.8	0.9	0.3	-1.2	9.2	0.0									
**12**	6.5	8.5	1.1	-0.7	0.4	0.1	0.1	-0.4	-0.7	3.0	182.1	0.0								
**13**	4.6	1.7	0.0	0.2	0.0	0.1	0.0	0.1	5.1	7.8	4.8	1.3	0.0							
**14**	1.0	-0.2	0.0	-0.1	0.0	0.2	-0.1	-0.1	2.8	1.1	1.8	0.3	147.5	0.0						
**15**	-0.3	0.4	0.2	-0.1	0.0	-0.1	-0.1	-0.2	0.9	0.0	3.8	-0.4	153.9	-9.3	0.0					
**16**	-0.2	0.1	-0.1	0.0	-0.1	0.1	0.0	0.1	-0.1	-0.5	0.3	0.2	23.6	-13.0	-2.0	0.0				
**17**	0.1	-0.1	0.0	0.0	0.0	0.0	-0.1	-0.1	0.0	0.1	-0.2	-0.2	-4.3	13.5	3.1	-67.5	0.0			
**18**	5.8	2.6	152.0	0.6	7.5	-1.8	7.6	0.4	-0.2	0.0	2.6	-0.3	0.0	-0.1	-0.2	0.0	-0.1	0.0		
**19**	0.9	1.2	-0.5	1.5	1.1	10.2	0.3	1.6	-0.3	0.0	0.0	0.2	0.0	0.0	0.0	0.0	0.0	0.3	0.0	
**20**	0.5	0.2	-0.4	-0.1	-2.2	-1.6	-0.1	0.8	-2.2	-0.2	-0.1	-0.1	0.0	0.0	0.0	0.0	0.0	0.1	31.4	0.0
**21**	-0.5	0.1	-0.8	0.2	-1.9	-0.7	0.0	-0.4	-0.1	0.1	-0.3	0.1	0.0	0.0	0.1	0.0	0.1	0.1	31.5	-8.8

IRC:

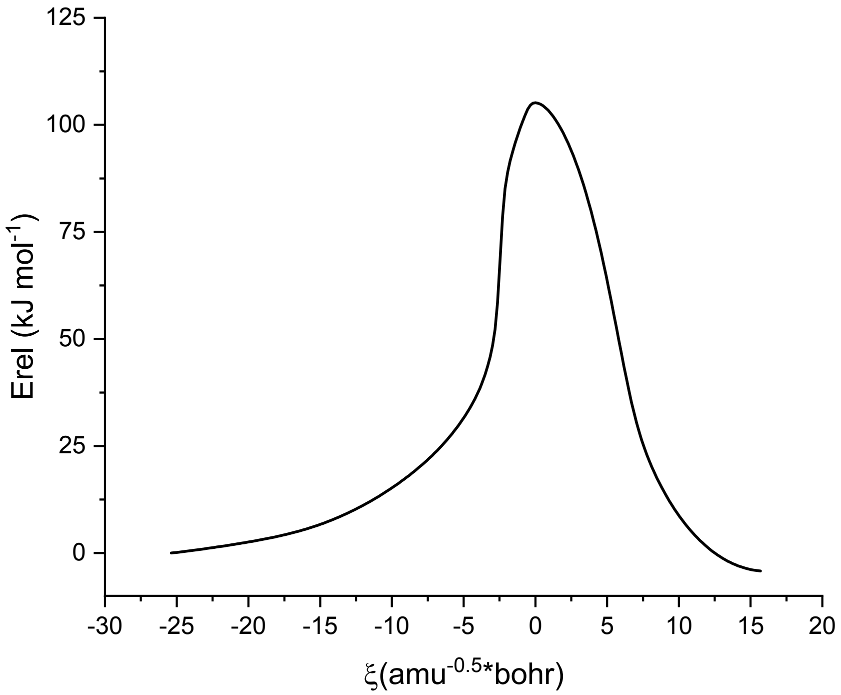

N1: **1aH$^+$·CH$_2$O·H$_2$O**	TS$_{N1}$	N1: **2aH$^+$·OH$_2$**

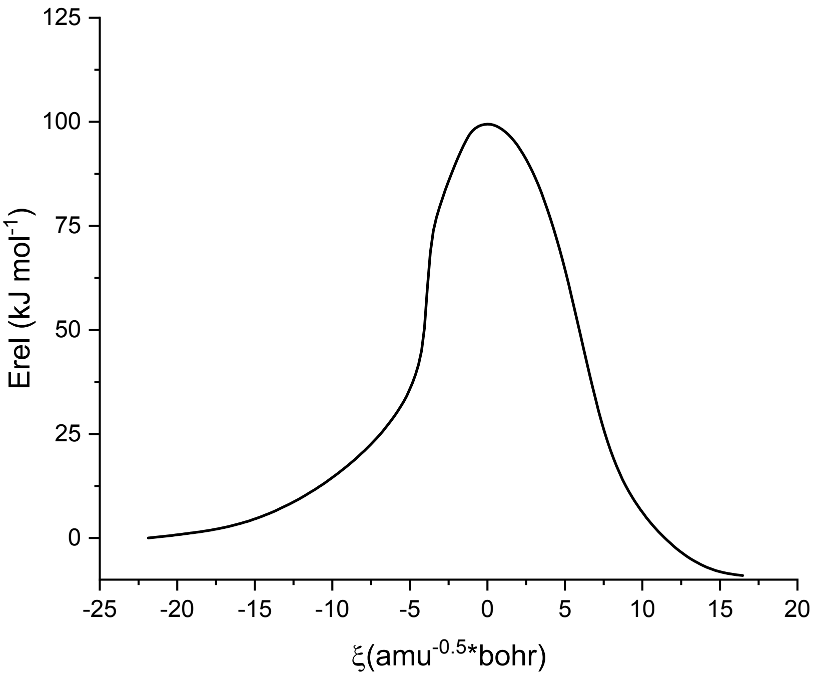

N2: **1aH$^+$·CH$_2$O·H$_2$O**	TS$_{N2}$	N2: **3aH$^+$·OH$_2$**

Table S2. Energies (kJ·mol^{-1}) corresponding to Scheme 6; x = **a**, **b**, **c**, **d**, **e**.

	x	B3LYP	B3LYP/ PCM	DLPNO (a)	DLPNO + PCM (b)	DLPNO- C-PCM (c)
N1-complex **1xH⁺·CH₂O·H₂O**	a	0.0	0.0	0.0	0.0	0.0
N1-TS	a	114.1	92.9	103.4	82.2	75.6
N1-adduct **2xH⁺·OH₂**	a	–17.1	–35.8	–35.8	–54.6	–55.7
N2-complex **1xH⁺·CH₂O·H₂O**	a	–5.3	–3.7	–3.9	–2.3	–2.6
N2-TS	a	104.5	83.0	96.1	74.6	68.2
N2-adduct **3xH⁺·OH₂**	a	–9.4	–23.3	–24.2	–38.1	–43.2
N1-complex **1xH⁺·CH₂O·H₂O**	b	0.0	0.0	0.0	0.0	
N1-TS	b	107.8	78.6	97.1	67.9	
N1-adduct **2xH⁺·OH₂**	b	–21.6	–29.8	–41.1	–49.3	
N2-complex **1xH⁺·CH₂O·H₂O**	b	–4.8	–3.2	–4.3	–2.7	
N2-TS	b	94.3	68.7	83.4	57.8	
N2-adduct **3xH⁺·OH₂**	b	–19.1	–37.1	–37.9	–55.9	
N1-complex **1xH⁺·CH₂O·H₂O**	c	0.0	0.0	0.0	0.0	
N1-TS	c	107.2	80.7	96.2	69.7	
N1-adduct **2xH⁺·OH₂**	c	–22.7	–30.8	–40.9	–48.9	
N2-complex **1xH⁺·CH₂O·H₂O**	c	–5.2	–3.4	–3.2	–1.4	
N2-TS	c	95.8	68.9	87.1	60.2	
N2-adduct **3xH⁺·OH₂**	c	–19.1	–37.0	–37.0	–54.9	
N1-complex **1xH⁺·CH₂O·H₂O**	d	0.0	0.0	0.0	0.0	
N1-TS	d	105.7	78.0	97.7	70.1	
N1-adduct **2xH⁺·OH₂**	d	–21.6	–31.4	–37.1	–46.9	
N2-complex **1xH⁺·CH₂O·H₂O**	d	–2.8	–4.3	1.5	0.1	
N2-TS	d	98.4	67.8	91.5	61.0	
N2-adduct **3xH⁺·OH₂**	d	–17.9	–38.0	–33.0	–53.1	
N1-complex **1xH⁺·CH₂O·H₂O**	e	0.0	0.0	0.0	0.0	
N1-TS	e					
N1-adduct **2xH⁺·OH₂**	e	1.0	–7.8	–20.8	–29.7	
N2-complex **1xH⁺·CH₂O·H₂O**	e	0.0	0.0	0.0	0.0	
N2-TS	e	100.1	71.6	90.3	61.8	
N2-adduct **3xH⁺·OH₂**	e	–16.3	–31.7	–36.9	–52.3	

(a) DLPNO/CCSD(T)/def2-TZVP//B3LYP/6-311++G(d,p)
(b) DLPNO/CCSD(T)/def2- TZVP//B3LYP/6-311++G(d,p) + solvation effect at B3LYP level
(c) DLPNO/CCSD(T)/def2- TZVP + C-PCM//B3LYP/6-311++G(d,p)

Correlation between columns (b) and (c): PCM(b) = (3.0±1.1) + (1.03±0.02) C-PCM(c), n = 6, R^2 = 0.998
